# Abstracts from the 8th International Conference on cGMP Generators, Effectors and Therapeutic Implications: Bamberg, Germany. 23-25 June, 2017

**DOI:** 10.1186/s40360-017-0170-5

**Published:** 2017-10-10

**Authors:** G. Todd Milne, on behalf of the Ironwood team, Peter Sandner, Kathleen A. Lincoln, Paul C. Harrison, Hongxing Chen, Hong Wang, Holly Clifford, Hu Sheng Qian, Diane Wong, Chris Sarko, Ryan Fryer, Jeremy Richman, Glenn A. Reinhart, Carine M. Boustany, Steven S. Pullen, Henriette Andresen, Lise Román Moltzau, Alessandro Cataliotti, Finn Olav Levy, Robert Lukowski, Sandra Frankenreiter, Andreas Friebe, Timothy Calamaras, Robert Baumgartner, Angela McLaughlin, Mark Aronovitz, Wendy Baur, Guang-Rong Wang, Navin Kapur, Richard Karas, Robert Blanton, Stefan Hell, Scott A. Waldman, Jieru E. Lin, Francheska Colon-Gonzalez, Gilbert W. Kim, Erik S. Blomain, Dante Merlino, Adam Snook, Jeanette Erdmann, Jana Wobst, Thorsten Kessler, Heribert Schunkert, Ulrich Walter, Oliver Pagel, Elena Walter, Stepan Gambaryan, Albert Smolenski, Kerstin Jurk, Rene Zahedi, James R. Klinger, Raymond L. Benza, Paul A. Corris, David Langleben, Robert Naeije, Gérald Simonneau, Christian Meier, Pablo Colorado, Mi Kyung Chang, Dennis Busse, Marius M. Hoeper, Jaime L. Masferrer, Sarah Jacobson, Guang Liu, Renee Sarno, Sylvie Bernier, Ping Zhang, G. Todd Milne, Roger Flores-Costa, Mark Currie, Katherine Hall, Dorit Möhrle, Katrin Reimann, Steffen Wolter, Markus Wolters, Evanthia Mergia, Nicole Eichert, Hyun-Soon Geisler, Peter Ruth, Andreas Friebe, Robert Feil, Ulrike Zimmermann, Doris Koesling, Marlies Knipper, Lukas Rüttiger, Yasutake Tanaka, Atsuko Okamoto, Takashi Nojiri, Motofumi Kumazoe, Takeshi Tokudome, Koichi Miura, Jun Hino, Hiroshi Hosoda, Mikiya Miyazato, Kenji Kangawa, Vikas Kapil, Amrita Ahluwalia, Nazareno Paolocci, Philip Eaton, James C. Campbell, Philipp Henning, Eugen Franz, Banumathi Sankaran, Friedrich W. Herberg, Choel Kim, M. Wittwer, Q. Luo, V. Kaila, S. A. Dames, Andrew Tobin, Mahmood Alam, Olena Rudyk, Susanne Krasemann, Kristin Hartmann, Oleksandra Prysyazhna, Min Zhang, Lan Zhao, Astrid Weiss, Ralph Schermuly, Philip Eaton, Amie J. Moyes, Sandy M. Chu, Reshma S. Baliga, Adrian J. Hobbs, Stylianos Michalakis, Regine Mühlfriedel, Christian Schön, Dominik M. Fischer, Barbara Wilhelm, Ditta Zobor, Susanne Kohl, Tobias Peters, Eberhart Zrenner, Karl Ulrich Bartz-Schmidt, Marius Ueffing, Bernd Wissinger, Mathias Seeliger, Martin Biel, RD-CURE consortium, Mark J. Ranek, Kristen M. Kokkonen, Dong I. Lee, Ronald J. Holewinski, Vineet Agrawal, Cornelia Virus, Donté A. Stevens, Masayuki Sasaki, Huaqun Zhang, Mathew M. Mannion, Peter P. Rainer, Richard C. Page, Jonathan C. Schisler, Jennifer E. Van Eyk, Monte S. Willis, David A. Kass, Manuela Zaccolo, Michael Russwurm, Jan Giesen, Corina Russwurm, Ernst-Martin Füchtbauer, Doris Koesling, Nadja I. Bork, Viacheslav O. Nikolaev, Luis Agulló, Martin Floor, Jordi Villà-Freixa, Ornella Manfra, Gaia Calamera, Nicoletta C. Surdo, Silja Meier, Alexander Froese, Viacheslav O. Nikolaev, Manuela Zaccolo, Finn Olav Levy, Kjetil Wessel Andressen, Annemarie Aue, Fabian Schwiering, Dieter Groneberg, Andreas Friebe, Gzona Bajraktari, Jürgen Burhenne, Walter E. Haefeli, Johanna Weiss, Katharina Beck, Barbara Voussen, Alexander Vincent, Sean P. Parsons, Jan D. Huizinga, Andreas Friebe, Fabiola Zakia Mónica, Edward Seto, Ferid Murad, Ka Bian, Joseph R. Burgoyne, Oleksandra Prysyazhna, Daniel Richards, Philip Eaton, Gaia Calamera, Marianne Bjørnerem, Andrea Hembre Ulsund, Jeong Joo Kim, Choel Kim, Finn Olav Levy, Kjetil Wessel Andressen, Sonia Donzelli, Mara Goetz, Kjestine Schmidt, Markus Wolters, Konstantina Stathopoulou, Oleksandra Prysyazhna, Jenna Scotcher, Christian Dees, Hariharan Subramanian, Elke Butt, Alisa Kamynina, S. Bruce King, Viacheslav O. Nikolaev, Cor de Witt, Lars I. Leichert, Robert Feil, Philip Eaton, Friederike Cuello, Hyazinth Dobrowinski, Moritz Lehners, Michael Paolillo Hannes Schmidt, Robert Feil, Susanne Feil, Lai Wen, Markus Wolters, Martin Thunemann, Kjestine Schmidt, Marcus Olbrich, Harald Langer, Meinrad Gawaz, Andreas Friebe, Cor de Wit, Robert Feil, Eugen Franz, Jeong Joo Kim, Daniela Bertinetti, Choel Kim, Friedrich W. Herberg, Hossein-Ardeschir Ghofrani, Friedrich Grimminger, Ekkehard Grünig, Yigao Huang, Pavel Jansa, Zhi Cheng Jing, David Kilpatrick, David Langleben, Stephan Rosenkranz, Flavia Menezes, Arno Fritsch, Sylvia Nikkho, Reiner Frey, Marc Humbert, Dieter Groneberg, Annemarie Aue, Fabian Schwiering, Andreas Friebe, Manuela Harloff, Joerg Reinders, Jens Schlossmann, Joon Jung, Jessica A. Wales, Cheng-Yu Chen, Linda Breci, Andrzej Weichsel, Sylvie G. Bernier, Robert Solinga, James E. Sheppeck, Paul A. Renhowe, William R. Montfort, Liying Qin, Ying-Ju Sung, Darren Casteel, Choel Kim, Alexander Kollau, Andrea Neubauer, Astrid Schrammel, Michael Russwurm, Doris Koesling, Bernd Mayer, Motofumi Kumazoe, Mika Takai, Chieri Takeuchi, Mai Kadomatsu, Shun Hiroi, Kanako Takamatsu, Takashi Nojiri, Kenji Kangawa, Hirofumi Tachibana, Marissa Opelt, Emrah Eroglu, Markus Waldeck-Weiermair, Michael Russwurm, Doris Koesling, Roland Malli, Wolfgang F. Graier, John T. Fassett, Astrid Schrammel, Bernd Mayer, Selene J. Sollie, Lise Román Moltzau, Maria Hernandez-Valladares, Frode Berven, Finn Olav Levy, Kjetil W. Andressen, Takashi Nojiri, Takeshi Tokudome, Motofumi Kumazoe, Miki Arai, Yutaka Suzuki, Koichi Miura, Jun Hino, Hiroshi Hosoda, Mikiya Miyazato, Meinoshin Okumura, Shinpei Kawaoka, Kenji Kangawa, Stefanie Peters, Hannes Schmidt, B. Selin Kenet, Sarah Helena Nies, Katharina Frank, Lai Wen, Fritz G. Rathjen, Robert Feil, Olga N. Petrova, Isabelle Lamarre, Michel Négrerie, Jerid W. Robinson, Jeremy R. Egbert, Julia Davydova, Laurinda A. Jaffe, Lincoln R. Potter, Jerid W. Robinson, Nicholas Blixt, Leia C. Shuhaibar, Gordon L. Warren, Kim C. Mansky, Laurinda A. Jaffe, Lincoln R. Potter, Simone Romoli, Tobias Bauch, Karoline Dröbner, Frank Eitner, Mihály Ruppert, Tamás Radovits, Sevil Korkmaz-Icöz, Shiliang Li, Péter Hegedűs, Sivakanan Loganathan, Balázs Tamás Németh, Attila Oláh, Csaba Mátyás, Kálmán Benke, Béla Merkely, Matthias Karck, Gábor Szabó, Ulrike Scheib, Matthias Broser, Shatanik Mukherjee, Katja Stehfest, Christine E. Gee, Heinz G. Körschen, Thomas G. Oertner, Peter Hegemann, Hannes Schmidt, Deborah M. Dickey, Alexandre Dumoulin, Ralf Kühn, Laurinda Jaffe, Lincoln R. Potter, Fritz G. Rathjen, Sophie Schobesberger, Peter Wright, Claire Poulet, Catherine Mansfield, Andreas Friebe, Sian E. Harding, Viacheslav O. Nikolaev, Julia Gorelik, Alexander Kollau, Marissa Opelt, Gerald Wölkart, Antonius C. F. Gorren, Michael Russwurm, Doris Koesling, Astrid Schrammel, Bernd Mayer, Gerburg K. Schwaerzer, Darren E. Casteel, Nancy D. Dalton, Yusu Gu, Shunhui Zhuang, Dianna M. Milewicz, Kirk L. Peterson, Renate Pilz, Fabian Schwiering, Annemarie Aue, Dieter Groneberg, Andreas Friebe, Aikaterini I. Argyriou, Garyfalia Makrynitsa, Ioannis I. Alexandropoulos, Andriana Stamopoulou, Marina Bantzi, Athanassios Giannis, Stavros Topouzis, Andreas Papapetropoulos, Georgios A. Spyroulias, Dennis J. Stuehr, Arnab Ghosh, Yue Dai, Saurav Misra, Boris Tchernychev, Joon Jung, Guang Liu, Inmaculada Silos-Santiago, Gerhard Hannig, Vu Thao-Vi Dao, Martin Deile, Pavel I. Nedvetsky, Andreas Güldner, César Ibarra-Alvarado, Axel Gödecke, Harald H. H. W. Schmidt, Angelos Vachaviolos, Andrea Gerling, Martin Thunemann, Stefan Z. Lutz, Hans-Ulrich Häring, Marcel A. Krüger, Bernd J. Pichler, Michael J. Shipston, Susanne Feil, Robert Feil, Sara Vandenwijngaert, Clara D. Ledsky, Obiajulu Agha, Dongjian Hu, Ibrahim J. Domian, Emmanuel S. Buys, Christopher Newton-Cheh, Donald B. Bloch, Barbara Voussen, Katharina Beck, Nadine Mauro, Jonas Keppler, Andreas Friebe, Wilson A. Ferreira, Hanan Chweih, Pamela L. Brito, Camila B. Almeida, Carla F. F. Penteado, Sara S. O. Saad, Fernando F. Costa, Paul S. Frenette, Damian Brockschnieder, Johannes-Peter Stasch, Peter Sandner, Nicola Conran, Daniel P. Zimmer, Jenny Tobin, Courtney Shea, Renee Sarno, Kimberly Long, Sarah Jacobson, Kim Tang, Peter Germano, James Wakefield, Ali Banijamali, G-Yoon Jamie Im, James E. Sheppeck, Albert T. Profy, G. Todd Milne, Mark G. Currie, Jaime L. Masferrer

**Affiliations:** 1 Ironwood Pharmaceuticals, Cambridge, MA 02142 USA; 2 Bayer AG, Drug Discovery - Cardiovascular Research, Wuppertal, Germany; 3 Department of Cardiometabolic Diseases Research, Boehringer Ingelheim Pharmaceuticals, Ridgefield, CT USA; 4 Department of Small Molecule Drug Research, Boehringer Ingelheim Pharmaceuticals, Ridgefield, CT USA; 5 Department of Pharmacology, University of Oslo and Oslo University Hospital, Oslo, Norway; 6 Institute of Experimental Medical Research, University of Oslo and Oslo University Hospital, Oslo, Norway; 7 Center for Heart Failure Research, University of Oslo and Oslo University Hospital, Oslo, Norway; 8 Department of Pharmacology, Toxicology and Clinical Pharmacy, Institute of Pharmacy, University of Tuebingen, Tuebingen, Germany; 9 Physiologisches Institut, Universität Würzburg, Würzburg, Germany; 10 Molecular Cardiology Research Institute, Tufts Medical Center, Boston, Massachusetts 02111 USA; 11 Present Affiliation: Division of Cardiology, University of Pittsburgh Medical Center, Pittsburgh, Pennsylvania ,15224 USA; 12 Present Affiliation: Department of Medicine, Brown University School of Medicine, Providence, Rhode Island ,02912 USA; 13 Division of Cardiology, Tufts Medical Center, Boston, Massachusetts 02111 USA; 14 Max Planck Institute for Biophysical Chemistry, Göttingen and German Cancer Research Center (DKFZ), Heidelberg, Germany; 15 Department of Pharmacology and Experimental Therapeutics, Thomas Jefferson University, Philadelphia, Pennsylvania 19107 USA; 16 Institut für Kardiogenetik and Universitäres Herzzentrum Lübeck, Universität zu Lübeck, Lübeck, Germany & DZHK e.V. (German Center for Cardiovascular Research), Partner Site Hamburg/Kiel/Lübeck, Lübeck, Germany; 17 Deutsches Herzzentrum München, Klinik für Herz- und Kreislauferkrankungen, Technische Universität München, Munich, Germany; 18 Center for Thrombosis & Hemostasis, Universitätsklinikum der Johannes Gutenberg-Universität Mainz, Mainz, Germany; 19 Leibniz-Institut für Analytische Wissenschaften – ISAS – e.V, Dortmund, Germany; 20 Sechenov Institute of Evolutionary Physiology and Biochemistry, Russian Academy of Sciences, St. Petersburg, Russia; 21 UCD Conway Institute, UCD School of Medicine and Medical Science, University College Dublin, Dublin, Ireland; 22 Division of Pulmonary, Sleep and Critical Care Medicine, Rhode Island Hospital, Alpert Medical School of Brown University, Providence, RI USA; 23 Cardiovascular Institute, Allegheny General Hospital, Pittsburgh, PA USA; 24 Institute of Cellular Medicine, Newcastle University, Newcastle, UK; 25 Center for Pulmonary Vascular Disease and Lady Davis Institute, Jewish General Hospital, McGill University, Montreal, QC Canada; 26 Department of Cardiology, Erasme University Hospital, Brussels, Belgium; 27 Assistance Publique–Hôpitaux de Paris, Service de Pneumologie, Hôpital Bicêtre, Université Paris-Sud, Laboratoire d’Excellence en Recherche sur le Médicament et Innovation Thérapeutique, and INSERM Unité 999, Le Kremlin–Bicêtre, France; 28 Global Clinical Development, Bayer AG, Berlin, Germany; 29 Global Clinical Development, Bayer HealthCare Pharmaceuticals, Barcelona, Spain; 30 Chrestos Concept GmbH & Co, Essen, Germany; 31 Clinic for Respiratory Medicine, Hannover Medical School, Hannover, Germany; 32 Ironwood Pharmaceuticals, Cambridge, MA 02142 USA; 33 Department of Biochemistry and Molecular Genetics, Hospital Clinic-University of Barcelona, Barcelona, Spain; 34 Department of Otolaryngology, Head and Neck Surgery, Hearing Research Centre Tübingen, Molecular Physiology of Hearing, University of Tübingen, Elfriede-Aulhorn-Straße 5, 72076 Tübingen, Germany; 35 Interfaculty Institute of Biochemistry, University of Tübingen, Hoppe-Seyler-Straße 4, 72076 Tübingen, Germany; 36 Department of Pharmacology and Toxicology, University of Bochum, Universitätsstr 150, 44780 Bochum, Germany; 37 Department of Pharmacology, Toxicology and Clinical Pharmacy, Institute of Pharmacy, University of Tübingen, Auf der Morgenstelle 8, 72076 Tübingen, Germany; 38 Department of Physiology, University of Würzburg, Röntgenring 9, 97070 Würzburg, Germany; 39 Department of Biochemistry, National Cerebral and Cardiovascular Center Research Institute, Suita, Osaka, Japan; 40 Department of General Thoracic Surgery, Osaka University Graduate School of Medicine, Suita, Osaka, Japan; 41 Department of Regenerative Medicine and Tissue Engineering, National Cerebral and Cardiovascular Center Research Institute, Suita, Osaka, Japan; 42 William Harvey Research Institute, Barts & The London School of Medicine, QMUL, London, UK; 43 Division of Cardiology, Department of Medicine, Johns Hopkins Medical Institutions, Baltimore, Maryland USA; 44 Department of Experimental Medicine, University of Perugia, 06123 Perugia, Italy; 45 King’s College London, Cardiovascular Division, The Rayne Institute, St Thomas’ Hospital, London, UK; 46 Structural and Computational Biology and Molecular Biophysics Program, Baylor College of Medicine, Houston, TX USA; 47 Department of Pharmacology, Baylor College of Medicine, Houston, TX USA; 48 Department of Biochemistry, University of Kassel, Kassel, Hesse Germany; 49 Berkeley Center for Structural Biology, Lawrence Berkeley National Laboratory, Berkeley, CA USA; 50 Verna and Marrs McLean Department of Biochemistry and Molecular Biology, Baylor College of Medicine, Houston, TX USA; 51 Technische Universität München, Department of Chemistry, Biomolecular NMR Spectroscopy, Garching, Germany; 52 Technische Universität München, Department of Chemistry, Computational Biocatalysis, Garching, Germany; 53 Institute of Structural Biology, Helmholtz Zentrum München, Neuherberg, Germany; 54 Institute of Molecular Cell and Systems Biology, University of Glasgow, Glasgow, UK; 55 BHF Centre of Research Excellence, King’s College London, London, SE1 7EH UK; 56 Core Facility for Mouse Pathology, University Medical Centre Hamburg-Eppendorf, 20246 Hamburg, Germany; 57 Faculty of Medicine, Department of Medicine, Imperial College London, London, SW7 2AZ UK; 58 Excellence Cluster Cardio-Pulmonary System, Universities of Giessen and Marburg Lung Centre Member of the German Lung Canter Justus-Liebig-University Giessen, Giessen, Germany; 59 William Harvey Research Institute, Barts & The London School of Medicine, QMUL, London, UK; 60 Center for Integrated Protein Science Munich CiPSM at the Department of Pharmacy – Center for Drug Research, Ludwig-Maximilians-Universität München, Munich, Germany; 61 Institute for Ophthalmic Research, Centre for OphthalmologyUniversity of Tübingen, Tübingen, Germany; 62 University Eye Hospital, Centre for Ophthalmology Tübingen, University of Tübingen, Tübingen, Germany; 63 STZ eyetrial at the Centre for Ophthalmology, University of Tübingen, Tübingen, Germany; 64 Division of Cardiology, Johns Hopkins Medical Institutions, Baltimore, MD 21205 USA; 65 Heart Institute and Advanced Clinical Biosystems Research Institute, Cedar Sinai Medical Center, 8700 Beverly Blvd, AHSP A9229, Los Angeles, California 90048 USA; 66 McAllister Heart Institute, Division of Cardiology, UNC, Chapel Hill, NC USA; 67 Department of Chemistry and Biochemistry, Miami University, Oxford, OH 45056 USA; 68 Department of Pyhisology, Anatomy and GeneticsUniversity of Oxford, Oxford, UK; 69 Institute for Pharmacology and Toxicology, Medical Faculty, Ruhr-University Bochum, 44780 Bochum, Germany; 70 Department of Molecular Biology and Genetics - Molecular Cell and Developmental Biology, Aarhus University, C.F. Møllers Allé 3, 8000 Aarhus C, Denmark; 71 Institute of Experimental Cardiovascular Research, Center for Experimental Medicine, University Medical Center Hamburg-Eppendorf, Hamburg, Germany; 72 Computational Biochemistry and Biophysics Laboratory (CBBL), U_ScienceTech (UST), University of Vic - Central University of Catalonia (UVIC-UCC), Vic, Barcelona Spain; 73 Department of Pharmacology, University of Oslo and Oslo University Hospital, Oslo, Norway; 74 Center for Heart Failure Research, University of Oslo, Oslo, Norway; 75 Department of Physiology, Anatomy and Genetics, Oxford University, Oxford, UK; 76 German Center for Cardiovascular Research, University Medical Center Hamburg-Eppendorf and Institute of Experimental Cardiovascular Research, Hamburg, Germany; 77 Physiologisches Institut, Universität Würzburg, Würzburg, Germany; 78 Department of Clinical Pharmacology and Pharmacoepidemiology, University of Heidelberg, Im Neuenheimer Feld 410, 69120 Heidelberg, Germany; 79 Physiologisches Institut, Universität Würzburg, Würzburg, Germany; 80 Farncombe Family Digestive Health Research Institute, McMaster University, Hamilton, Canada; 81 Department of Biochemistry and Molecular Medicine, George Washington University, School of Medicine, Washington, DC USA; 82 Department of Pharmacology, Faculty of Medical Sciences, State University of Campinas (UNICAMP), Campinas, Sao Paolo Brazil; 83 King’s College London, Cardiovascular Division, The British Heart Foundation Centre of Excellence, The Rayne Institute, St Thomas’ Hospital, London, SE1 7EH UK; 84 Department of Pharmacology, University of Oslo and Oslo University Hospital, Oslo, Norway; 85 Center for Heart Failure Research, University of Oslo, Oslo, Norway; 86 Department of Pharmacology, Baylor College of Medicine, Houston, Texas USA; 87 Department of Experimental Pharmacology and Toxicology, Cardiovascular Research Center, University, Medical Center Hamburg-Eppendorf, Martinistrasse 52, 20246 Hamburg, Germany; 88 DZHK (German Center for Cardiovascular Research), partner site Hamburg/Kiel/Lübeck, University Medical Center Hamburg-Eppendorf, Martinistrasse 52, 20246 Hamburg, Germany; 89 Department of Physiology, University of Lübeck, DZHK Partner Site Hamburg/Kiel/Lübeck, University Heart Centre Lübeck, Lübeck, Germany; 90 Interfakultäres Institut für Biochemie, University of Tübingen, Hoppe-Seyler-Str. 4, 72076 Tübingen, Germany; 91 King’s College London, Cardiovascular Division, British Heart Foundation Centre of Excellence, the Rayne Institute, St Thomas´ Hospital, SE17EH, London, UK; 92 Institute of Experimental Biomedicine II, University Medical Center Würzburg, Grombühlstraße 12, 97080 Würzburg, Germany; 93 Institute of Experimental Cardiovascular Research, University Medical Center Hamburg-Eppendorf, Martinistrasse 52, 20246 Hamburg, Germany; 94 Department of Chemistry, Wake Forest University, Winston-Salem, North Carolina 27109 USA; 95 Institute of Biochemistry and Pathobiochemistry - Microbial Biochemistry, Ruhr University Bochum, Universitätsstrasse 150, 44780 Bochum, Germany; 96 Interfakultäres Institut für Biochemie, University of Tübingen, Tübingen, Germany; 97 Interfakultäres Institut für Biochemie, Universität Tübingen, Tübingen, Germany; 98 Institut für Physiologie, Universität zu Lübeck, Lübeck, Germany; 99 Department of Cardiology & Cardiovascular Medicine, University of Tübingen, Tübingen, Germany; 100 Physiologisches Institut, Universität Würzburg, Würzburg, Germany; 101 Department of Biochemistry, University of Kassel, 34132 Kassel, Germany; 102 Department of Pharmacology, Baylor College of Medicine, One Baylor Plaza, Houston, Texas USA; 103 Department of Internal Medicine, University of Giessen and Marburg Lung Center (UGMLC), Giessen, Germany; 104 Centre for Pulmonary Hypertension at the Thoraxclinic of University Hospital Heidelberg, Heidelberg, Germany; 105 Department of Cardiology, Guangdong General Hospital and Guangdong Cardiovascular Institute, Guangzhou, Guangdong China; 106 Department of Cardiology and Angiology of the First Faculty of Medicine and General Teaching Hospital, Prague, Czech Republic; 107 State Key Laboratory of Cardiovascular Disease FuWai Hospital, National Center for Cardiovascular DiseasePeking Union Medical College and Chinese Academy of Medical Sciences, Beijing, China; 108 Discipline of Medicine, University of Tasmania, Hobart, Australia; 109 Center for Pulmonary Vascular Disease, McGill University, Montreal, QC Canada; 110 Department III of Internal Medicine and Cologne Cardiovascular Research Centre, Cologne University Heart Centre, Cologne, Germany; 111 Bayer HealthCare Pharmaceuticals, São Paulo, Brazil; 112 Global Clinical Development, Bayer AG, Wuppertal, Germany; 113 Global Clinical Development, Bayer AG, Berlin, Germany; 114 Université Paris-Sud, Université Paris-Saclay, Le Kremlin Bicêtre, France; 115 Physiologisches Institut, Universität Würzburg, Würzburg, Germany; 116 Department of Pharmacology and Toxicology, University of Regensburg, 93053 Regensburg, Germany; 117 Department Functional Genomics, University of Regensburg, 93053 Regensburg, Germany; 118 Ironwood Pharmaceuticals, Cambridge, MA 02142 USA; 119 Department of Chemistry and Biochemistry, University of Arizona, Tucson, AZ 8572 USA; 120 Verna and Marrs McLean Department of Biochemistry and Molecular Biology, Baylor College of Medicine, Houston, Texas USA; 121 Geisinger Commonwealth School of Medicine, Commonwealth Medical College, Scranton, PA 18509 USA; 122 Department of Medicine, University of California, San Diego, La Jolla, California, USA; 123 Department of Pharmacology, Baylor College of Medicine, Houston, Texas USA; 124 Department of Pharmacology and Toxicology, University of Graz, A 8010, Graz, Austria; 125 Department of Pharmacology and Toxicology, Ruhr University Bochum, D 44780 Bochum, Germany; 126 Division of Applied Biological Chemistry, Department of Bioscience and Biotechnology, Faculty of Agriculture, Kyushu University, 6-10-1 Hakozaki, Higashi-ku, Fukuoka, 812-8581 Japan; 127 Department of Biochemistry, National Cerebral and Cardiovascular Center Research Institute, 5-7-1 Fujishiro-dai, Suita-City, Osaka 565-8565 Japan; 128 Institute of Pharmaceutical Sciences, Department of Pharmacology and Toxicology, University of Graz, Graz, Austria; 129 Institute of Molecular Biology and Biochemistry, Center of Molecular Medicine, Medical University of Graz, Graz, Austria; 130 Department of Pharmacology and Toxicology, Ruhr University Bochum, Bochum, Germany; 131 Department of Pharmacology, Faculty of Medicine, University of Oslo and Oslo University Hospital, Oslo, Norway; 132 Center for Heart Failure Research, Faculty of Medicine, University of Oslo, Oslo, Norway; 133 Department of Biomedicine, Faculty of Medicine and Dentistry, University of Bergen, Bergen, Norway; 134 Department of Biochemistry, National Cerebral and Cardiovascular Center Research Institute, Suita, Osaka, Japan; 135 Department of General Thoracic Surgery, Osaka University Graduate School of Medicine, Suita, Osaka, Japan; 136 Advanced Telecommunications Research Institute International (ATR), The Thomas N. Sato BioMEC-X Laboratories, Kyoto, Japan; 137 The University of Tokyo, graduate school of frontier science, Kashiwa, Japan; 138 Department of Regenerative Medicine and Tissue Engineering, National Cerebral and Cardiovascular Center Research Institute, Suita, Osaka, Japan; 139 Interfakultäres Institut für Biochemie, University of Tübingen, Tübingen, Germany; 140 Max-Delbrück-Centrum für Molekulare Medizin, Berlin, Germany; 141 Present address: Institut für Zellbiologie des Nervensystems, München, TU Germany; 142 Present address: Institut für Immunologie, BioMedical Center, LMU, Planegg-Martinsried, Germany; 143 Present address: Interfakultäres Institut für Biochemie, University of Tübingen, Tübingen, Germany; 144 Laboratory for Optics and Biosciences, Ecole Polytechnique, Palaiseau, France; 145 Department of Biochemistry, Molecular Biology, and Biophysics, University of Minnesota, Minneapolis, MN USA; 146 Department of Cell Biology, University of Connecticut Health Center, Farmington, CT USA; 147 Department of Surgery, University of Minnesota, Minneapolis, MN USA; 148 Department of Pharmacology, University of Minnesota, Minneapolis, MN USA; 149 Department of Biochemistry, Molecular Biology, and Biophysics, University of Minnesota, Minneapolis, MN USA; 150 Department of Developmental and Surgical Sciences, University of Minnesota, Minneapolis, MN USA; 151 Department of Cell Biology, University of Connecticut Health Center, Farmington, CT USA; 152 Department of Physical Therapy, Georgia State University, Atlanta, GA USA; 153 Department of Pharmacology, University of Minnesota, Minneapolis, MN USA; 154 Cardiovascular Research II, Therapeutic Research Group, Drug Discovery, Bayer AG, Wuppertal, Germany; 155 Department of Cardiac Surgery, University of Heidelberg, Heidelberg, Germany; 156 Heart and Vascular Center, Semmelweis University, Budapest, Hungary; 157 Humboldt Universität zu Berlin, Institute for Biology, Experimental Biophysics, Berlin, Germany; 158 University Medical Center Hamburg-Eppendorf, Center for Molecular Neurobiology Hamburg, Institute for Synaptic Physiology, Hamburg, Germany; 159 Center of Advanced European Studies and Research (caesar) Bonn, Department: Molecular Sensory Systems, Bonn, Germany; 160 Interfaculty Institute of Biochemistry, University of Tübingen, Tübingen, Germany; 161 Max Delbrück Center for Molecular Medicine in the Helmholtz Association, Berlin, Germany; 162 Department of Biochemistry, University of Minnesota, Minneapolis, Minnesota USA; 163 Department of Cell Biology, University of Connecticut Health Center, Farmington, Connecticut USA; 164 Institute of Experimental Cardiovascular Research, University Medical Center Hamburg-Eppendorf, 20246 Hamburg, Germany; 165 Department of Myocardial Function, Imperial College London, Hammersmith Hospital, W12 0NN, London, England UK; 166 Institute of Physiology, Universität Würzburg, 97070 Würzburg, Germany; 167 Department of Pharmacology and Toxicology, University of Graz, A 8010 Graz, Austria; 168 Department of Pharmacology and Toxicology, Ruhr University Bochum, D 44780 Bochum, Germany; 169 Department of Medicine, University of California San Diego, La Jolla, California, 92093 USA; 170 Division of Medical Genetics and Cardiology, Department of Internal Medicine, The University of Texas Health Science Center at Houston, Houston, Texas 77030 USA; 171 Physiologisches Institut, Universität Würzburg, Würzburg, Germany; 172 Department of Pharmacy, University of Patras, GR-26504 Patras, Greece; 173 Institute of Organic Chemistry, Leipzig University, D-04103 Leipzig, Germany; 174 School of Health Sciences, Faculty of Pharmacy, University of Athens, GR-15 771 Athens, Greece; 175 Department of Pathobiology, Lerner Research Institute, Cleveland Clinic, Cleveland, Ohio 44195 USA; 176 Kansas State University, Manhattan, Kansas 66506 USA; 177 Ironwood Pharmaceuticals, Cambridge, MA 02142 USA; 178 Decibel Therapeutics, Cambridge, MA 02142 USA; 179 Department for Pharmacology and Personalized Medicine, CARIM, FHML, Maastricht University, Maastricht, The Netherlands; 180 Department of Pharmacology, Johannes Gutenberg University, Mainz, Germany; 181 Asklepios-Klinik Radeberg, Dresden, Germany; 182 Vesalius Research Center, Katholieke Universiteit Leuven, Leuven, Belgium; 183 Residency Anaesthesiology, Department of Anaesthesiology und Critical Care Medicine, Technische Universität, Dresden, Germany; 184 Facultad de Química, Universidad Autónoma de Querétaro, Santiago de Querétaro, Mexico; 185 Institut für Herz- und Kreislaufphysiologie Heinrich-Heine-Universität, Düsseldorf, Germany; 186 Interfakultäres Institut für Biochemie, University of Tübingen, Tübingen, Germany; 187 Department of Internal Medicine, Division of Endocrinology, Diabetology, Vascular Disease, Nephrology and Clinical Chemistry, University of Tübingen, Tübingen, Germany; 188 Werner Siemens Imaging Center, Department of Preclinical Imaging and Radiopharmacy, University of Tübingen, Tübingen, Germany; 189 Centre for Integrative Physiology, School of Biomedical Science, Hugh Robson Building, University of Edinburgh, Edinburgh, Scotland EH8 9XD UK; 190 Department of Anesthesia, Critical Care, and Pain Medicine, Massachusetts General Hospital and Harvard Medical School, Boston, Massachusetts USA; 191 Cardiovascular Research Center, Department of Medicine, Massachusetts General Hospital and Harvard Medical School, Boston, Massachusetts USA; 192 Harvard Stem Cell Institute, Cambridge, Massachusetts USA; 193 Program in Medical and Population Genetics, Broad Institute, Cambridge, Massachusetts USA; 194 Center for Genomic Medicine, Massachusetts General Hospital and Harvard Medical School, Boston, Massachusetts USA; 195 Division of Rheumatology, Allergy and Immunology, Department of Medicine, Massachusetts General Hospital and Harvard Medical School, Boston, Massachusetts USA; 196 Physiologisches Institut, Universität Würzburg, Würzburg, Germany; 197 Hematology Centre, School of Medical Sciences, University of Campinas (UNICAMP), Sao Paolo, Brazil; 198 Bayer AG, Pharmaceuticals - Drug Discovery, Wuppertal, Germany; 199 Ruth L. and David S. Gottesman Institute for Stem Cell and Regenerative Medicine Research, Albert Einstein College of Medicine, Bronx, NY USA; 200 Ironwood Pharmaceuticals, Cambridge, MA 02142 USA

## A1 Characterization and development of next-generation sGC stimulators

### G. Todd Milne^1^ (tmilne@ironwoodpharma.com), on behalf of the Ironwood team

#### ^1^Ironwood Pharmaceuticals, Cambridge, MA 02142, USA

The nitric oxide (NO)-soluble guanylate cyclase (sGC)-cyclic guanosine monophosphate (cGMP) signalling pathway plays a fundamental role in modulating diverse physiological processes including blood flow, fibrosis, inflammation, and metabolism. sGC stimulators are small-molecule, heme-dependent agonists of sGC that synergize with and enhance endogenous NO signaling. As such, sGC stimulators may provide therapeutic benefits both in diseases associated with impaired NO signaling and in diseases where stimulation of this pathway will restore functional homeostasis. Data from our recent preclinical studies add to the growing body of evidence that sGC stimulators have direct effects on systemic and vascular inflammation, fibrosis, and metabolism. Ironwood is developing IW-1973 and IW-1701 as oral, once-daily sGC stimulators for both cardiovascular and non-cardiovascular systemic disease indications. Phase 1 studies in healthy human subjects demonstrated clear evidence of target engagement, attractive pharmacokinetic properties, and predicted hemodynamic effects, at well-tolerated doses. Phase 2 studies are currently ongoing in patients with achalasia, an esophageal motility disorder, and in patients with diabetes and hypertension. Preclinical characterization of IW-1973 and IW-1701 support the broad therapeutic potential and multi-faceted pharmacology of these compounds. Based on preclinical studies, IW-1973 has extensive distribution into organs including liver, heart, kidney, and lung, which may maximize effects on target organs while limiting systemic hemodynamic effects. The pharmacokinetic profile of IW-1701 has a narrow peak-to-trough ratio, which may provide more consistent pharmacological effect throughout the dosing interval. Ironwood is also developing IW-6463, a novel, CNS-penetrant sGC stimulator that shows target engagement and effects on regional blood flow in the brain. Preclinical data suggest that IW-6463 may be useful in treating CNS disorders including vascular dementia and Alzheimer’s disease. We believe that sGC stimulation, alone or in combination with other mechanisms, may afford therapeutic benefit in multiple diseases. Furthermore, there may be an opportunity to provide targeted treatments by selecting compounds that are well-suited for specific diseases based on pharmacological profile, tissue distribution, pharmacokinetics, and route of administration**.**

**Competing interest**

*Todd Milne and Ironwood team are employees and shareholders of Ironwood Pharmaceuticals and are developing sGC stimulators for therapeutic applications.*

## A2 sGC stimulators and the potential impact for the treatment of fibrotic diseases and systemic sclerosis

### Peter Sandner^1^ (peter.sandner@bayer.com)

#### ^1^Bayer AG, Drug Discovery - Cardiovascular Research, Wuppertal, Germany

The NO/cGMP signaling cascade plays a pivotal role in regulation of the cardiovascular system. The relaxation of vascular smooth muscle cells is one key mechanism by which NO-driven cGMP elevation is reducing blood vessel tone. Therefore, stimulators of the soluble guanylate cyclase (sGC stimulators), which significantly stimulate cGMP production, can cause a dose-dependent relaxation of blood vessels with impact e.g. on pulmonary hemodynamics. The sGC stimulator riociguat can e.g. reduce pulmonary artery pressure and is approved for the treatment of pulmonary hypertension (PAH/CTEPH). In recent years, it became obvious that the second messenger molecule cGMP does not only induce smooth muscle cell relaxation but might also target a variety of other cells and tissues and could also influence tissue remodeling and fibrosis. Therefore, the application of cGMP-increasing sGC stimulators and sGC activators might have a substantially broader treatment potential with additional therapeutic applications also for the treatment of fibrotic diseases.

Given the potential antifibrotic mode of action of sGC stimulators, we investigated if sGC stimulators could become a potential treatment option for Systemic Sclerosis (SSc). SSc is a connective tissue disease, characterized by excessive skin fibrosis, but also fibrosis of internal organs as lungs and kidneys, causing a high morbidity and increased mortality. There is still a significant unmet medical, especially for approved antifibrotic treatments since only symptomatic treatment options and treatment recommendations are currently available.

The sGC stimulators BAY 41-2272 and BAY 63-2521 (riociguat) were profiled in vitro and in vivo in preclinical models of SSc and skin fibrosis. In vitro, the sGC stimulators reduced collagen production in human dermal fibroblasts, reduced fibroblast-to-myofibroblast differentiation of human dermal fibroblasts, reduced established myofibroblasts and inhibited transforming growth factor beta (TGF-ß) signaling. In vivo, the sGC stimulators prevented TGFß-induced skin fibrosis, prevented skin fibrosis in the bleomycin model, reduced skin fibrosis in the genetic tight skin (TSK-1) mouse model, reduced skin and intestinal fibrosis in the chronic graft versus host disease (cGvHD) mouse model and promoted wound healing in TSK-1 mice. These in vivo effects were seen at dosages which do not cause prohibitive blood pressure lowering effects.

In summary, these preclinical results demonstrated in a broad set of animal models for SSc and skin fibrosis with various etiologies, a dose-dependent regression of skin fibrosis and improvement of wound healing. These data therefore, suggested to further explore the treatment potential of the sGC stimulator riociguat in SSc patients. Currently a randomized, double-blind, placebo-controlled phase II study to investigate the efficacy and safety of riociguat in patients with Systemic Sclerosis (dcSSc), RISE-SSc, is ongoing (NCT02283762).

## A3 A soluble guanylate cyclase activator is superior to a phosphodiesterase type 5 inhibitor and a soluble guanylate cyclase stimulator in protecting from diabetic nephropathy in the ZSF1 rat

### Kathleen A. Lincoln^1^, Paul C. Harrison^1^, Hongxing Chen^1^, Hong Wang^1^, Holly Clifford^1^, Hu Sheng Qian^1^, Diane Wong^2^, Chris Sarko^2^, Ryan Fryer^1^, Jeremy Richman^1^, Glenn A. Reinhart^1^, Carine M. Boustany^1^, Steven S. Pullen^1^

#### ^1^Department of Cardiometabolic Diseases Research, Boehringer Ingelheim Pharmaceuticals, Ridgefield, CT, USA; ^2^Department of Small Molecule Drug Research, Boehringer Ingelheim Pharmaceuticals, Ridgefield, CT, USA

##### **Correspondence:** Steven S. Pullen**(**steven.pullen@boehringer-ingelheim.com**)**

**Background:**

Therapies which restore cyclic GMP (cGMP) levels within the kidney are hypothesized to slow disease progression. We evaluated the effects of BI703704, a soluble guanylate cyclase (sGC) activator, EX76637, a sGC stimulator, and EX77619, a phosphodiesterase type 5 (PDE5) inhibitor on the progression of diabetic nephropathy in obese ZSF1 rats.

**Methods:**

Male ZSF1 rats, implanted with telemetry devices, were treated with either BI703704 at 2 mg/kg, EX76637 at 1 or 3 mg/kg, or EX77619 at 5 or 15 mg/kg for 10 weeks, during which mean arterial pressure (MAP) and urinary protein excretion (UPE) were determined. At study end, glomerular and renal interstitial lesions were assessed. Alpha smooth muscle actin (α-SMA, a marker of myofibroblast activation) and p57 (a marker of podocyte health) were determined by immunohistochemistry. Renal cGMP levels were quantified as a measure of target engagement.

**Results:**

By Week 10, similar reductions in MAP were achieved (~8 mmHg) across treatment groups vs. vehicle. In parallel, sGC activation resulted in significant reductions in UPE (-31% vs veh), while there was no significant effect of EX76637 (+4% at 1 mg/kg; -16 % at 3 mg/kg vs veh), or EX77619 (-15% at 5 mg/kg; -9% at 15 mg/kg vs veh). Importantly, the effects of BI703704 on UPE were accompanied by reductions in the incidence of glomerulosclerosis (-21% vs veh), while neither EX76637 nor EX77619 was effective. In addition, interstitial lesions were modestly reduced by BI703704 (-13 % vs veh) and EX77619 (-18% vs veh). α-SMA was reduced by BI703704 (-27% vs veh) and EX76637 (-8% vs veh), while p57 was significantly increased by BI703704 (+11% vs veh) but not by EX76637 or EX77619. Importantly, target engagement was confirmed for EX77619.

**Conclusions:**

Despite similar effects on MAP, BI703704 was superior to EX76637 or EX77619 in reducing proteinuria and preventing renal damage in kidneys of ZSF1 rats.

**Competing interest**

*Steven Pullen is an employee of Boehringer Ingelheim Pharmaceuticals.*

## A4 Identification and characterization of positive allosteric modulators of the natriuretic peptide receptor-A

### Henriette Andresen^1,2^, Lise Román Moltzau^1,3^, Alessandro Cataliotti^2,3^, Finn Olav Levy^1,3^

#### ^1^Department of Pharmacology, University of Oslo and Oslo University Hospital, Oslo, Norway; ^2^Institute of Experimental Medical Research, University of Oslo and Oslo University Hospital, Oslo, Norway; ^3^Center for Heart Failure Research, University of Oslo and Oslo University Hospital, Oslo, Norway

##### **Correspondence:** Henriette Andresen **(**henriette.andresen@studmed.uio.no**)**

**Background:**

Natriuretic peptides play an important role in the regulation of blood pressure. Hypertension is associated with an impaired natriuretic peptide system and a reduced natriuretic peptide receptor-A (NPR-A) activation. Atrial (ANP) and brain natriuretic peptide (BNP) activate NPR-A, causing production of cyclic GMP (cGMP). Administration of recombinant BNP has shown effective blood pressure reduction in uncontrolled hypertension, suggesting BNP as a potential treatment of uncontrolled hypertension [1]. However, as a potential antihypertensive drug, small molecular compounds could offer better pharmaceutical properties, such as oral administration and longer half-life than peptides. Our aim was therefore to identify small molecular NPR-A agonists and characterize their pharmacological properties.

**Results and Conclusion:**

After performing a high throughput screening of about 30,000 small molecular compounds, we identified one compound that enhanced the cGMP production in NPR-A expressing cells. Through hit-to-lead optimization, we identified compounds with higher potency. All of these compounds were characterized as positive allosteric modulators. In the presence of a small concentration of BNP, these compounds increased the cGMP production in a concentration-dependent manner, and were also able to increase the maximum BNP-mediated cGMP production. The compounds were selective towards NPR-A, with little or no effect on cGMP production in NPR-B expressing cells. Although further optimization and characterization of these compounds are needed, positive allosteric modulators of NPR-A can provide a novel mechanism of action in the treatment of uncontrolled hypertension.

**Reference:**

[1]. Cataliotti A, Costello-Boerrigter LC, Chen HH, Textor SC, Burnett JC, Jr: **Sustained blood pressure-lowering actions of subcutaneous B-type natriuretic peptide (nesiritide) in a patient with uncontrolled hypertension.** *Mayo Clinic proceedings* 2010. **87**:413-5

**Competing interest**

*No conflicts of interest.*

## A5 Cardioprotective actions of the cGMP pathway in ischemia and reperfusion injury

### Robert Lukowski^1^ and Sandra Frankenreiter^1^

#### ^1^Department of Pharmacology, Toxicology and Clinical Pharmacy, Institute of Pharmacy, University of Tuebingen, Tuebingen, Germany

##### **Correspondence:** Robert Lukowski (robert.lukowski@uni-tuebingen.de)

Experimentally used as well approved agents that act via nitric oxide-sensitive guanylyl cyclase (NO-GC) and cyclic guanosine-3',5'-monophosphate (cGMP)-degrading phosphodiesterase 5 (PDE5) reportedly exhibited protection against cardiac ischemia and reperfusion (I/R) injury. As NO-GC/cGMP, PDE5 and their major effector cGMP-dependent protein kinase type I (cGKI) are ubiquitously present across different cells types in the heart and circulation*,* neither the exact cells nor their precise involvement in the cardioprotective mechanisms are clear. We herein assessed whether beneficial effects of the cGMP pathway in the cardiomyocyte require voltage and Ca^2+^-activated K^+^ channels of the BK-type to oppose the myocardial damage during *in vivo* I/R injury.

*The authors declare no conflict of interest.*

Acknowledgements

Robert Lukowski and Sandra Frankenreiter are members of the DFG Research Unit 2060 “cGMP signalling in cell growth and survival”.

## A6 NO/cGMP signaling in cells ‘off the beaten track’

### Annemarie Aue, Katharina Beck, Dieter Groneberg, Fabian Schwiering, Barbara Voussen, Andreas Friebe

#### Physiologisches Institut, Universität Würzburg, Würzburg, Germany

##### **Correspondence:** Andreas Friebe (andreas.friebe@uni-wuerzburg.de)

NO-sensitive guanylyl cyclase (NO-GC) is accepted to be the major receptor for the signaling molecule NO. NO-GC is strongly expressed in platelets and vascular smooth muscle cells. Accordingly, the functions of NO-GC regarding hemostasis and vascular tone have been investigated intensively. However, the role of NO/cGMP signaling in many organs involves NO-GC in cells that have not been in broad focus. Using cell-specific knockout strains for NO-GC in combination with fluorescent reporter mice, e.g. tdTomato, we are able to investigate NO-GC function in cells that have received less attention over the years.

In all organs investigated so far, NO-GC is strongly expressed in pericytes. Pericytes are a heterogeneous group of cells as they express different markers depending on age, development or pathophysiological condition. Two of the best markers, PDGFRβ and desmin, are colocalized with NO-GC in murine lung. Pericytes in culture show αSMA expression after stimulation with TGFβ; accordingly, αSMA, a marker of fibrosis, is strongly upregulated in situ after bleomycin treatment to induce fibrosis. A potential modulatory role of NO/cGMP signaling is indicated by the fact that absence of NO-GC leads to an increased collagen and αSMA expression upon fibrosis induction.

In the gastrointestinal tract, NO-GC is expressed in smooth muscle cells but also in interstitial cells of Cajal (ICC) and so-called fibroblast like cells (FLC). Whereas the function of NO/cGMP in FLC still awaits clarification, we can show that NO-GC in ICC serves to guarantee nitrergic neurotransmission as well as development of spontaneous contractions.

In addition, we have identified NO-GC in colonic myofibroblasts. These myofibroblasts differ from those in lung by the fact that they are thought to constitute the stem cell niche for epithelial proliferation. Deletion of NO-GC using the SMMHC promotor leads to loss of the enzyme in SMC but also in colonic myofibroblasts. SMC-GCKO mice develop severe adenomas between 4-6 months of age. The role of NO-GC in these cells regarding hyperplastic colon polyps is currently under investigation.

***Competing interest***

*The authors declare no conflict of interest.*

## A7 Mixed lineage kinase 3 (MLK3), a novel PKGIα substrate, prevents pressure overload-induced cardiac remodeling

### Timothy Calamaras^1,^ Robert Baumgartner^1,2^, Angela McLaughlin^1,3^, Mark Aronovitz^1^, Wendy Baur^1^, Guang-Rong Wang^1^, Navin Kapur^1,4^, Richard Karas^1,4^, Robert Blanton^1,4^

#### ^1^Molecular Cardiology Research Institute, Tufts Medical Center, Boston, Massachusetts, 02111, USA; ^2^Current Affiliation: Division of Cardiology, University of Pittsburgh Medical Center, Pittsburgh, Pennsylvania,15224, USA; ^3^Current Affiliation: Department of Medicine, Brown University School of Medicine, Providence, Rhode Island,02912, USA; ^4^Division of Cardiology, Tufts Medical Center, Boston, Massachusetts, 02111, USA

##### **Correspondence:** Timothy Calamaras (tcalamaras@tuftsmedicalcenter.org)

The cGMP-dependent protein kinase G I (PKGI) opposes cardiac hypertrophy and dysfunction, and PKGI-activating drugs remain under investigation for the treatment of heart failure. The downstream mechanisms through which PKGI promotes cardioprotection remain incompletely understood, suggesting that identifying PKGI substrates may reveal novel therapeutic targets in the treatment of cardiovascular disease. We previously identified the PKGI leucine zipper (LZ) binding domain to be a critical regulator of cardiac remodeling in response to cardiac pressure overload, and therefore sought to explore PKGI LZ binding substrates as novel cardioprotective molecules.

Here we investigated one candidate PKGIα substrate in the myocardium, Mixed Lineage Kinase 3 (MLK3), an upstream regulator of stress-responsive JNK signaling. We first observed protein-protein interaction of endogenous PKGIα and MLK3 in myocardium by co-immunoprecipitation (n=5). Direct PKGIα-MLK3 interaction was confirmed with affinity purified proteins (n=4). In primary cardiomyocytes MLK3 mediated cGMP-stimulated JNK phosphorylation (n=3), and pharmacological MLK3 kinase inhibition induced cardiomyocyte hypertrophy (n=4, +27.0%). MLK3 protein expression was detected in human hearts, increased in myocardium from patients with both nonischemic and hypertrophic cardiomyopathy (MLK3/GAPDH: non-ischemic: 6.26 ADU ± 0.85, n=9, hypertrophic: 6.97 ADU ± 1.42, n=8), and in hearts of mice subjected to cardiac pressure overload (n=8, +138.0%).

MLK3 knockout mice (MLK3^-/-^) exhibited baseline cardiac hypertrophy with preserved cardiac function and structure. In response to pressure overload, MLK3^-/-^ mice developed accelerated cardiac dysfunction as measured by invasive hemodynamics (n=5-8, LV ejection fraction, dP/dT max, dP/dT min, and LV end diastolic pressure) compared to MLK3^+/+^ littermate controls. Pressure overloaded MLK3^-/-^ hearts had increased hypertrophic (ANP) and fibrotic (Col1a1) gene expression changes in the heart (n=3-6) suggesting accelerated cardiac remodeling. Mechanistically, pressure overloaded MLK3^-/-^ mice exhibited selective impairments in myocardial JNK activation, with no change in activation of other MAPK proteins.

Together these data demonstrate MLK3 is a novel PKGIα substrate, MLK3 inhibits adverse cardiac remodeling *in vivo*, and suggests regulation of myocardial JNK signaling may underlie the cardioprotective actions of MLK3. This study further supports the approach of exploring myocardial PKGI-substrates to identify novel cardioprotective molecules.

***Competing interest***

*The authors have declared that no conflict of interest exists.*

## A8 Optical microscopy: the resolution revolution

### Stefan Hell (hell@nanoscopy.de)

#### Max Planck Institute for Biophysical Chemistry, Göttingen and German Cancer Research Center (DKFZ), Heidelberg, Germany

Throughout the 20^th^ century it was widely accepted that a light microscope relying on conventional optical lenses cannot discern details that are much finer than about half the wavelength of light (200-400 nm), due to diffraction. However, in the 1990s, the viability to overcome the diffraction barrier was realized and microscopy concepts defined, that can resolve fluorescent features down to molecular dimensions. In this lecture, I will discuss the simple yet powerful principles that allow neutralizing the limiting role of diffraction^1,2^. In a nutshell, feature molecules residing closer than the diffraction barrier are transferred to different (quantum) states, usually a bright fluorescent state and a dark state, so that they become discernible for a brief period of detection. Thus, the resolution-limiting role of diffraction is overcome, and the interior of transparent samples, such as living cells and tissues, can be imaged at the nanoscale.

**References:**

1. Hell, S.W. Far-Field Optical Nanoscopy. *Science* **316**, 1153-1158 (2007).

2. Hell, S.W. Microscopy and its focal switch. *Nature Methods* **6**, 24-32 (2009).

## A9 Caloric silencing of GUCY2C-hormone axes at the nexus of obesity and colorectal cancer

### Scott A. Waldman, Jieru E. Lin, Francheska Colon-Gonzalez, Gilbert W. Kim, Erik S. Blomain, Dante Merlino, Adam Snook

#### Department of Pharmacology and Experimental Therapeutics, Thomas Jefferson University, Philadelphia, Pennsylvania, 19107, USA

##### **Correspondence:** Scott A. Waldman (scott.waldman@jefferson.edu)

Obesity is a global pandemic and >1.5 billion adults are overweight (BMI >25 kg/m^2^), including 500 million who are obese (BMI >30 kg/m^2^). Annual US healthcare costs of obesity exceed $150 billion, and by 2030 will exceed 20% of those costs. Obesity reflects over-nutrition, in which calories eaten exceed those expended metabolically, in part reflecting dysregulated satiety responses controlling appetite. Beyond the established cardiovascular and metabolic sequelae contributing to this morbidity and mortality, there is an established relationship between body mass and the risk of cancer, including colorectal cancer. Indeed, obese patients have ~20-60% greater risk of, and ~2-fold higher death rate from, colorectal cancer. Although the epidemiology of this relationship is well-established, the precise molecular mechanisms connecting obesity and colorectal cancer remain to be defined. GUCY2C is the receptor for the paracrine hormones guanylin in the colorectum and uroguanylin in small intestine. An emerging paradigm suggests that guanylin loss disrupting the GUCY2C paracrine signaling axis, and epithelial cell homeostasis, is a required step in colorectal cancer initiation. Separately, secretion of uroguanylin into the circulation by the small intestine forms a gut-brain endocrine axis controlling hypothalamic GUCY2C regulating satiety and appetite linked to body mass and metabolic homeostasis. Here, we reveal that over-nutrition and consumption of excess calories suppresses guanylin and uroguanylin expression, simultaneously disrupting GUCY2C paracrine and endocrine signaling axes at the nexus of obesity and colorectal cancer. Expression of uroguanylin and guanylin, but not GUCY2C, is reduced in small and large intestine, respectively, by diet-induced obesity in mice and humans. Hormone expression appears to be reversibly suppressed by ingested calories through endoplasmic reticulum stress. In that context, transgenic replacement of guanylin in intestine eliminates tumorigenesis induced by obesity. Further, transgenic expression of uroguanylin in brain improves satiety responses dysregulated in obesity. These observations suggest a novel pathophysiological model in which caloric suppression of guanylin and uroguanylin silencing GUCY2C is at the intersection of molecular mechanisms underlying obesity and its associated risk of colorectal cancer. Moreover, they reveal a correlative therapeutic paradigm which leverages the preservation of GUCY2C expression in over-nutrition, in which hormone supplementation reconstitutes endocrine and paracrine axes to restore appetite control opposing obesity and intestinal epithelial cell homeostasis preventing tumorigenesis, respectively.

**References:**

[1]. JE Lin, F Colon-Gonzalez, E Blomain, GW Kim, A Aing, B Stoecker, J Rock, AE Snook, T Zhan, T Hyslop, M Tomczak, RS Blumberg, SA Waldman: **Calories suppress guanylin silencing the GUCY2C tumor suppressor in colorectal cancer in obesity.** *Can. Res.* 2016, **76**:339-346

[2]. GW Kim, JE Lin, AE Snook, A Aing, DJ Merlino, P Li, SA Waldman: **Calorie-induced ER stress suppresses uroguanylin satiety signaling in diet-induced obesity.** *Nutr Diabetes* 2016, **23**;6:e211. doi: 10.1038/nutd.2016.18

## A10 sGC and atherosclerosis: a genomic approach

### Jeanette Erdmann^1^, Jana Wobst^*2*^, Thorsten Kessler^*2*^, Heribert Schunkert^*2*^

#### ^1^Institut für Kardiogenetik and Universitäres Herzzentrum Lübeck, Universität zu Lübeck, Lübeck, Germany & DZHK e.V. (German Center for Cardiovascular Research), Partner Site Hamburg/Kiel/Lübeck, Lübeck, Germany; ^2^Deutsches Herzzentrum München, Klinik für Herz- und Kreislauferkrankungen, Technische Universität München, Munich, Germany

##### **Correspondence:** Jeanette Erdmann (jeanette.erdmann@uni-luebeck.de)

Soluble guanylate cyclase (sGC), a key enzyme of the nitric oxide signaling pathway, is formed as a heterodimer by various isoforms of its α and β subunit. sGC is the physiological receptor for nitric oxide (NO) and NO-releasing drugs. Its activation induces the synthesis of the second messenger cGMP. cGMP regulates the activity of various downstream proteins, leading to vascular relaxation, inhibition of platelet aggregation, and modified neurotransmission.

Recently, genomic studies - genome-wide association studies (GWAS) in large samples of unrelated probands and exome-sequencing studies (WES) in extended families presenting with myocardial infarction - revealed the importance of rare and common genetic variation of sGC and other genes involved in NO-signalling, like NOS3, encoding the endothelial NO-synthase, and PDE5A, on cardiovascular risk.

Understanding the full spectrum of phenotypic consequences of rare and common variants may provide insight into the benefits and risks of pharmacologic manipulation of nitric oxide signalling.

## A11 The human platelet phosphoproteome after sGC stimulation by Riociguat

### Ulrich Walter^1^, Oliver Pagel^2^, Elena Walter^1^, Stepan Gambaryan^1,3^, Albert Smolenski^4^, Kerstin Jurk^1^, Rene Zahedi^2^

#### ^1^Center for Thrombosis & Hemostasis, Universitätsklinikum der Johannes Gutenberg-Universität Mainz, Mainz, Germany; ^2^Leibniz-Institut für Analytische Wissenschaften – ISAS – e.V., Dortmund, Germany; ^3^Sechenov Institute of Evolutionary Physiology and Biochemistry, Russian Academy of Sciences, St. Petersburg, Russia; ^4^UCD Conway Institute, UCD School of Medicine and Medical Science, University College Dublin, Dublin, Ireland

##### **Correspondence:** Ulrich Walter **(**ulrich.walter@uni-mainz.de**);** Kerstin Jurk^1^; Rene Zahedi^2^

**Background:**

Platelets are circulating sentinels of vascular integrity and are activated, inhibited or modulated by multiple hormones, vasoactive substances or drugs. Endothelium- or drug-derived NO strongly inhibits platelet activation via activation of the soluble guanylyl cyclase (sGC) and cGMP elevation, often in synergy with the cAMP- elevating prostacyclin. However, the molecular mechanisms and diversity of cGMP effects in platelets are poorly understood and sometimes controversial. Recently, we established the quantitative human platelet proteome^1^, the Iloprost/prostacyclin/cAMP/PKA affected phosphoproteome^2^, the interaction of the ADP and Iloprost/prostacyclin-affected phosphoproteome^3^ and the effects of Riociguat on human platelets^4^. Here, the aim was to establish and analyze the phosphoproteome after selective stimulation of the sGC/cGMP pathway by Riociguat in human platelets.

**Methods:**

Phosphorylation and functional platelet effects of various NO donors (Sodium nitroprusside, DEA-NO, Sodium S-Nitrosocysteine) and the sGC stimulator Riociguat were compared. For the full phosphoproteomic study the effect of Riociguat (10 μM, 5 min) was investigated as the most robust and cAMP-independent cGMP-effector reagent in platelets and compared to the phosphoproteome of Iloprost (5 nM, 2 min)measured in parallel as described^2, 3^.

**Results:**

In total we quantified 8181 phosphorylation sites from 2249 proteins across three biological replicates. Riociguat increased (>1.5-fold up) and decreased (>1.5-fold down) phosphorylation levels in 345 and 94 proteins, respectively. The spectrum of proteins covers many platelet functions including membrane proteins/receptors, signaling molecules and granule proteins including 24 protein kinases (e.g. MYLK, CAMKK1/2, CDK16/17/18, BRAF) with increased and 4 protein kinases (KALRN, KSR2, PAK2, WNK1) with reduced levels. Comparison of the Riociguat phosphoproteome with the Iloprost (cAMP/PKA) phosphoproteome showed that there is a significant overlap of cGMP and cAMP responses at the level of protein phosphorylation but there are a number of proteins more strongly up-regulated by Riociguat. The top-list of Riociguat affected phosphoproteins include established PKG substrates (VASP, MYLK, ITPR1, PDE5A, MRVI1 and others) but also others previously not described as PKG substrates.

**Conclusion:**

This is the first description of the human platelet phosphoproteome affected by selective stimulation of the sGC/cGMP pathway. The magnitude and diversity of the Riociguat/cGMP phosphoproteome is extensive and significantly overlaps with the Iloprost/cAMP phosphoproteome. However, there are number of novel PKG substrates and some distinct differences between cAMP and cGMP pathway. The regulated phosphorylation of multiple protein kinases and signalling molecules by the cGMP/PKG system indicates a substantial network.

**References:**

[1]. Burkhart JM, Vaudel M, Gambaryan S, Radau S, Walter U, Martens L, Geiger J, Sickmann A and Zahedi RP. **The first comprehensive and quantitative analysis of human platelet protein composition allows the comparative analysis of structural and functional pathways.** *Blood*. 2012;120:E73-E82.

[2]. Beck F, Geiger J, Gambaryan S, Veit J, Vaudel M, Nollau P, Kohlbacher O, Martens L, Walter U, Sickmann A and Zahedi RP. **Time-resolved characterization of cAMP/PKA-dependent signaling reveals that platelet inhibition is a concerted process involving multiple signaling pathways.** *Blood*. 2014;123:E1-E10.

[3]. Beck F, Geiger J, Gambaryan S, Solari FA, Dell'Aica M, Loroch S, Mattheij NJ, Mindukshev I, Potz O, Jurk K, Burkhart JM, Fufezan C, Heemskerk JW, Walter U, Zahedi RP and Sickmann A. **Temporal quantitative phosphoproteomics of ADP stimulation reveals novel central nodes in platelet activation and inhibition.** *Blood*. 2017;129:e1-e12.

[4]. Reiss C, Mindukshev I, Bischoff V, Subramanian H, Kehrer L, Friebe A, Stasch JP, Gambaryan S and Walter U. **The sGC stimulator riociguat inhibits platelet function in washed platelets but not in whole blood.** *British Journal of Pharmacology*. 2015;172:5199-5210.

## A12 The relationship between NO pathway biomarkers and response to riociguat in the RESPITE study of patients with PAH not reaching treatment goals with phosphodiesterase 5 inhibitors

### James R Klinger^1^, Raymond L Benza^2^, Paul A Corris^3^, David Langleben^4^, Robert Naeije^5^, Gérald Simonneau^6^, Christian Meier^7^, Pablo Colorado^8^, MiKyung Chang^7^, Dennis Busse^9^, Marius M Hoeper^10^

#### ^1^Division of Pulmonary, Sleep and Critical Care Medicine, Rhode Island Hospital, Alpert Medical School of Brown University, Providence, RI, USA; ^2^Cardiovascular Institute, Allegheny General Hospital, Pittsburgh, PA, USA; ^3^Institute of Cellular Medicine, Newcastle University, Newcastle, UK; ^4^Center for Pulmonary Vascular Disease and Lady Davis Institute, Jewish General Hospital, McGill University, Montreal, QC, Canada; ^5^Department of Cardiology, Erasme University Hospital, Brussels, Belgium; ^6^Assistance Publique–Hôpitaux de Paris, Service de Pneumologie, Hôpital Bicêtre, Université Paris-Sud, Laboratoire d’Excellence en Recherche sur le Médicament et Innovation Thérapeutique, and INSERM Unité 999, Le Kremlin–Bicêtre, France; ^7^Global Clinical Development, Bayer AG, Berlin, Germany; ^8^Global Clinical Development, Bayer HealthCare Pharmaceuticals, Barcelona, Spain; ^9^Chrestos Concept GmbH & Co., Essen, Germany; ^10^Clinic for Respiratory Medicine, Hannover Medical School, Hannover, Germany

##### **Correspondence:** James R Klinger **(**james_klinger@brown.edu**)**

**Background:**

A proportion of pulmonary arterial hypertension (PAH) patients fail to reach/maintain treatment goals with phosphodiesterase 5 inhibitors (PDE5i). RESPITE investigated whether it is safe, feasible, and beneficial to replace PDE5i with riociguat in PAH patients with an inadequate response to PDE5i. This analysis explored the relationship between the nitric oxide (NO) signaling biomarkers including cyclic guanosine monophosphate (cGMP, a 2nd messenger that mediates smooth muscle relaxation via protein kinase G) and asymmetric dimethylarginine (ADMA, an inhibitor of NO synthesis), and response to riociguat.

**Methods**

RESPITE (NCT02007629) was a 24-week, open-label, single-arm, Phase IIIb trial in PAH patients in World Health Organization (WHO) functional class (FC) III, with 6-minute walking distance (6MWD) 165–440 m, cardiac index <3.0 L/min/m^2^, pulmonary vascular resistance >400 dyn·s·cm^−5^, and mean pulmonary arterial pressure >30 mmHg, despite PDE5i treatment for ≥90 days. Patients underwent a 1–3-day PDE5i treatment-free period before receiving riociguat individually adjusted up to a maximum of 2.5 mg three times daily. Concomitant endothelin receptor antagonists (ERAs) were allowed. Exploratory endpoints included change from baseline to Week 24 in 6MWD, WHO FC, *N*-terminal prohormone of brain natriuretic peptide (NT-proBNP), and NO signaling-related and other biomarkers.

**Results:**

Sixty-one patients (mean±SD age 54±14 years; 74% female) were enrolled and 51 (84%) completed the study. Patients were pretreated with sildenafil (n=40 [66%]) or tadalafil (n=21 [34%]); 50 (82%) were taking concomitant ERAs at baseline. At Week 24, 6MWD and WHO FC were improved, plasma cGMP had increased, and NT-proBNP decreased compared with baseline (Table 1). Levels of cGMP and NT-proBNP correlated with PAH severity at baseline and Week 24. Sixteen patients (34% [n=47]) achieved the composite endpoint of no clinical worsening, WHO FC I/II, and ≥30 m increase in 6MWD. These patients had lower NT-proBNP and cGMP at baseline compared with those who did not achieve the composite endpoint. Serious adverse events occurred in 10 patients (16%), 2 of which (3%) were study drug-related.

**Table 1:** **(Abstract A12).** Change from baseline in biomarkers at Week 24 in RESPITE

Mean (SD) biomarker	Baseline		Week 24		
	n	Value at baseline	n	Change from baseline	p-value for change from baseline to Week 24
cGMP (plasma), pmol/mL	53	16.25 (11.24)	51	+2.61 (8.00)	0.0240
ADMA (plasma), μmol/mL	52	0.56 (0.13)	49	–0.02 (0.12)	0.2909
NT-proBNP, pg/mL	60	1190 (1828)	51	–347 (1235)	0.0170^a^
GDF-15, pg/mL	53	4633 (4525)	51	–669 (2977)	0.1150
ST-2, ng/mL	53	21.08 (15.17)	51	–2.34 (9.80)	0.0947

^a^p-value is for the relative change from baseline in NT-proBNP

Baseline = the last documented value while still receiving PDE5i

ADMA, asymmetric dimethylarginine; cGMP, cyclic guanosine monophosphate; GDF-15, growth differentiation factor 15; NT-proBNP, *N*-terminal prohormone of brain natriuretic peptide; ST-2, suppression of tumorigenicity 2

**Conclusions:**

RESPITE demonstrated that riociguat improved WHO FC and 6MWD as well as decreasing NT-proBNP and increasing plasma cGMP levels among PAH patients who had an inadequate response to PDE5i. No new safety signals were observed. The study provides preliminary evidence that switching from PDE5i to riociguat may be beneficial in PAH patients who are not at treatment goal with PDE5i. Further studies are needed to confirm if biomarker levels may help identify patients who would benefit from switching to riociguat.

Conflict of interest:

*James Klinger has received research support for Actelion, Bayer AG, Gilead Sciences, Ikaria, Lung Biotechnology, NH-NHLBI, Pfizer and United Therapeutics and personal fees for United Therapeutics and Bayer AG.*

*Raymond L. Benza has received grant fees from Bayer AG.*

*Paul A. Corris has received grant fees from Bayer AG (University Research Fund) and personal fees from Bayer AG, Actelion and GSK.*

*David Langleben has received personal fees and non-financial support from Bayer Healthcare Pharmaceuticals, Actelion, Gilead, GSK and Ikaria.*

*Robert Naeije has received personal fees from Actelion and GSK, grant fees from Reata and advisory board member fees from Actelion, Bayer AG and Lung Biotechnology Corporation.*

*Gérald Simonneau has received grant fees and personal fees from Actelion, Bayer AG, GSK, Novartis and Lilly and non-financial support from Pfizer.*

*Christian Meier is an employee of Bayer AG.*

*Pablo Colorado is an employee of Bayer Healthcare Pharmaceuticals.*

*MiKyung Chang is an employee of Bayer AG.*

*Dennis Busse is an employee of Chrestos Concept GmbH & Co. KG.*

*Marius M Hoeper has received personal fees (lectures and consultations) from Actelion, Bayer AG, Gilead, GSK, Pfizer and MSD.*

Acknowledgements

Marius M Hoeper is a member of the German Center for Lung Research (DZL).

## A13 The soluble guanylate cyclase stimulator, IW-1973, is efficacious in models of NASH and liver fibrosis

### Jaime L Masferrer^1^, Sarah Jacobson^1^, Guang Liu^1^, Renee Sarno^1^, Sylvie Bernier^1^, Ping Zhang, G. Todd Milne^1^, Roger Flores-Costa^2^, Mark Currie^1^ and Katherine Hall^1^

#### ^1^Ironwood Pharmaceuticals, Cambridge, MA, 02142, USA; ^2^Department of Biochemistry and Molecular Genetics, Hospital Clinic-University of Barcelona, Barcelona, Spain

##### **Correspondence: (**Jaime L Masferrer**)** jmasferrer@ironwoodpharma.com

**Introduction:**

Non-alcoholic steatohepatitis (NASH), characterized by liver fibrosis, inflammation, and steatosis, increases a patient’s risk for developing cirrhosis. IW-1973, a novel soluble guanylate cyclase stimulator enhances signalling through the nitric oxide-sGC-cGMP pathway and has demonstrated effects on blood flow, inflammation and fibrosis in a variety of tissues in multiple animal models. The aim of this study was to assess the effects of IW-1973 in rodent models of NASH and fibrosis.

**Methods:**

IW-1973 effects on TGFβ induced α-SMA expression were measured in rat liver stellate cells. The effects of acute and chronic dosing of IW-1973 on liver cGMP production and pVASP were determined in normal rats. To test the effects of IW-1973 in NASH and fibrosis, two murine models were used. In the MCD model, mice were fed a methionine and choline deficient high-fat diet and continually dosed for 9-weeks with IW-1973 at 1 and 3 mg/kg/day. In the STAM model, IW-1973 was administered therapeutically at 1.5,3 and 10 mg/kg/day. The Thioacetamide (TAA)-induced liver fibrosis model was used with rats; IW-1973 was dosed therapeutically at 1,3 and 10 mg/kg. Fibrosis was analysed using histological and immunohistochemical staining. Inflammation and fibrosis gene expression profiles were performed in livers using b-DNA technology.

**Results:**

IW-1973 (10 μM) increased cGMP production in rat stellate cells from 12.7 +/- 0.1 to 74.7 +/- 1.1 nM. When IW-1973 was added to the stellate cells 4-days after TGFβ (2.5 ng/mL for 7 days) it inhibited the profibrogenic activity by > 75%. Oral dosing with IW-1973 in healthy animals resulted in increased liver cGMP and pVASP compared to vehicle controls (p<0.01) indicating activation of the NO-sGC-cGMP pathway. IW-1973 reduced inflammation, steatosis and fibrosis in the MCD model at plasma levels that have not produced hemodynamic effects in normal mice. Similarly, in the STAM model, IW-1973 reduced COL1A1 mRNA, hydroxyproline and Sirius red markers of fibrosis at the 10 mg/kg dose (P<0.05). IW-1973 was also effective in reducing liver fibrosis in the TAA rat model. The antifibrotic effect was observed at all doses (P<0.05).

**Conclusions:**

IW-1973 is a novel sGC stimulator and enhancer of NO signaling. Increases in cGMP by IW-1973 stimulation in rat stellate cells reversed TGFβ induction of α-SMA production. IW-1973 stimulated the sGC-cGMP pathway in rat livers and dose-dependently reduced liver fibrosis and NASH in animal models at doses that do not affect blood pressure.

***Competing interests***

*Ironwood authors are employees of and own stock or stock options of Ironwood Pharmaceuticals. Ironwood Pharmaceuticals funded the research.*

## A14 NO-sensitive guanylate cyclase isoforms NO-GC1 and NO-GC2 contribute to noise-induced inner hair cell synaptopathy

### Dorit Möhrle^1^, Katrin Reimann^1^, Steffen Wolter^1^, Markus Wolters^2^, Evanthia Mergia^3^, Nicole Eichert^1^, Hyun-Soon Geisler^1^, Peter Ruth^4^, Andreas Friebe^5^, Robert Feil^2^, Ulrike Zimmermann^1^, Doris Koesling^3^, Marlies Knipper^1^, Lukas Rüttiger^1^

#### ^1^Department of Otolaryngology, Head and Neck Surgery, Hearing Research Centre Tübingen, Molecular Physiology of Hearing, University of Tübingen, Elfriede-Aulhorn-Straße 5, 72076, Tübingen, Germany; ^2^Interfaculty Institute of Biochemistry, University of Tübingen, Hoppe-Seyler-Straße 4, 72076, Tübingen, Germany; ^3^Department of Pharmacology and Toxicology, University of Bochum, Universitätsstr 150, 44780, Bochum, Germany; ^4^Department of Pharmacology, Toxicology and Clinical Pharmacy, Institute of Pharmacy, University of Tübingen, Auf der Morgenstelle 8, 72076, Tübingen, Germany; ^5^Department of Physiology, University of Würzburg, Röntgenring 9, 97070, Würzburg, Germany

##### **Correspondence: (**Dorit Möhrle**)** dorit.moehrle@uni-tuebingen.de; Katrin Reimann

**Background:**

Studies over the last decade have investigated the function of nitric oxide (NO) in the cochlea. NO activates the NO-sensitive guanylate cyclase and triggers intracellular signal transduction pathways involving cGMP. For cochlear hair cells, the role of NO-mediated cascades is controversial, with studies predicting a protective or detrimental potential. We examine here the cochlear function of mice lacking one of the two NO-sensitive guanylate cyclase isoforms (NO-GC1 KO or NO-GC2 KO). The deletion of NO-GC1 or NO-GC2 did not influence electromechanical outer hair cell (OHC) properties, measured by distortion product otoacoustic emissions (DPOAEs), neither before nor after noise exposure, nor were click or noise burst-evoked auditory brainstem responses (ABR) thresholds different from controls. Yet, inner hair cell (IHC) ribbons and auditory nerve responses were significantly less deteriorated in NO-GC1 KO and NO-GC2 KO mice after noise exposure. Consistent with a selective role of NO-GC in IHCs, NO-GC β1 mRNA is present in isolated IHCs but not in OHCs. Using transgenic mice expressing the FRET-based cGMP biosensor cGi500, NO-induced elevation of cGMP was detected in real-time in IHCs but not in OHCs. Importantly, long-term treatment with NO-GC stimulants led to a decline of auditory nerve response without change in OHC function in older but not younger animals.

**Conclusion:**

We conclude that cGMP signaling via NO-GC participates in the development of dysfunctional connections between auditory nerve fibers and sensory cells (synaptopathy) after noise exposure. Cochlear synaptopathy is thought to cause perceptual hearing deficits in a significant number of patients who have clinically normal hearing thresholds (hidden hearing loss). NO-GC isoforms may thus be future pharmacological targets for early treatment of noise trauma and prevention of hidden hearing loss.

Conflict of interest

*none.*

Funding

*This work was supported by the Deutsche Forschungsgemeinschaft [Grants FOR 2060 project FE 438/6-1, FR 1725/3-1, RU 713/3-2]; and University of Tübingen, Tübingen, Germany [Fortüne 2339-0-0].*

## A15 ANP-GC-A signaling protects against acute exacerbation of pulmonary fibrosis in mice

### Yasutake Tanaka^1^, Atsuko Okamoto^1^, Takashi Nojiri^1*, 2^, Motofumi Kumazoe^1^, Takeshi Tokudome^1^, Koichi Miura^1^, Jun Hino^1^, Hiroshi Hosoda^3^, Mikiya Miyazato^1^, Kenji Kangawa^1^

#### ^1^Department of Biochemistry, National Cerebral and Cardiovascular Center Research Institute, Suita, Osaka, Japan; ^2^Department of General Thoracic Surgery, Osaka University Graduate School of Medicine, Suita, Osaka, Japan; ^3^Department of Regenerative Medicine and Tissue Engineering, National Cerebral and Cardiovascular Center Research Institute, Suita, Osaka, Japan

##### **Correspondence:** Yasutake Tanaka **(**nojiri@ri.ncvc.go.jp**)**

**Clinical background:**

Idiopathic pulmonary fibrosis (IPF) is a form of interstitial lung disease that results in fibrosis of the lungs. Acute exacerbation of IPF (AE-IPF) is defined as a sudden acceleration of the disease or an idiopathic acute injury superimposed on diseased lung that leads to a significant decline in lung function. AE-IPF is associated with a high mortality rate among IPF patients. Since no prophylactic treatments for AE-IPF have been established, the novel therapeutic strategies are strongly demanded.

We have previously reported that atrial natriuretic peptide (ANP), an endogenous peptide produced by the heart, has protective effects on bleomycin-induced pulmonary fibrosis through vascular endothelial cells in mice. Motivated by the previous findings, we examined the effects of ANP on AE-IPF models in mice.

In AE-IPF models, we administered LPS (acute exacerbation model) 21 days after bleomycin administration (IPF model) via oropharyngeal aspiration. ANP (0.5 μg/kg/min) or vehicle was subcutaneously infused via an osmotic mini-pump two days before LPS challenge, and the infusion continued until the mice were euthanized one day after LPS challenge. In bronchoalveolar lavage (BAL) analysis, both total and macrophage cell counts were significantly elevated in the vehicle-treated group compared to the normal control group. ANP significantly decreased total and macrophage cell counts compared to vehicle. Quantitative assessment of the number of inflamed cells, as determined by Mac3 staining, demonstrated that ANP significantly attenuated the number of inflammatory cells in the lungs compared to vehicle. Consistently, ANP reduced the concentrations of IL-1β, IL-6, KC, MIP-1β, and MCP-1 in BAL fluid compared to vehicle. In summary, ANP can attenuate acute exacerbation in the lung induced by LPS in bleomycin-induced pulmonary fibrosis.

**Conclusion:**

Altogether, we concluded that ANP-GC-A signaling as a promising target for controlling IPF and AE-IPF.

## A16 Inorganic nitrate and the enterosalivary circuit: an alternative pathway for NO delivery in cardiovascular disease

### Vikas Kapil & Amrita Ahluwalia

#### William Harvey Research Institute, Barts & The London School of Medicine, QMUL, London, United Kingdom

##### **Correspondence:** Vikas Kapil **(**v.kapil@qmul.ac.uk**)**

It is now accepted that inorganic nitrate, through its sequential chemical reduction to nitrite and then to nitric oxide (NO), provides a source of NO in the body that exerts a number of important actions upon the cardiovascular system including blood pressure lowering; effects that are mediated by cyclic GMP (cGMP). Whilst the second step of this process is due to the activity of mammalian nitrite reductases, including xanthine oxidoreductase, the former (i.e. the chemical reduction of nitrate to nitrite) is critically dependent upon the activity of commensal bacteria, particularly residing within the oral cavity.

We and others have speculated that the entero-salivary circuit of inorganic nitrate offers an opportunity to target NO/cGMP signalling in the cardiovascular system in disease via a pathway that circumvents the classical, but dysfunctional, L-arginine/NO synthase (NOS) pathway. This proposal stems from observations indicating that supplementation of inorganic nitrate via dietary means (vegetables, particularly green leafy vegetables have a high inorganic nitrate content) or through nitrate salt administration exerts blood pressure lowering, anti-inflammatory and anti-thrombotic effects in patients with cardiovascular disease. In addition, evidence suggests that dietary inorganic nitrate not only provides beneficial effects acutely but that these positive actions are sustained with chronic administration and do not suffer tachyphylaxis. This difference in pharmacokinetics intimates that unlike the organic nitrates, inorganic nitrate provides sustained NO delivery with persistent administration and thus provides an approach to NO delivery that might prove useful in the therapeutics of chronic disease. In this presentation, the circuit for bioactivation of inorganic nitrate in the body and the key role that bacteria play in this process will be addressed. In addition, clinical studies supporting a role for nitrate bioactivation in setting blood pressure, and the potential for use of this circuit in the therapeutics of cardiovascular disease will be described.

## A17 From Heaven to Heart: Nitroxyl (HNO) Actions in the Cardiovascular System

### Nazareno Paolocci^1,2^ (npaoloc1@jhmi.edu)

#### ^1^Division of Cardiology, Department of Medicine, Johns Hopkins Medical Institutions, Baltimore, Maryland, USA; ^2^Department of Experimental Medicine, University of Perugia, Perugia, 06123, Italy

Reactions based on the transfer of one or more electrons from a donor (reductant) to an acceptor (oxidant) account for many physiologically relevant cellular processes. Both reactive oxygen (ROS) and nitrogen species (RNS) can signal through these reduction/oxidation (redox) reactions, particularly via reversible interaction with reactive thiols, namely cysteines. Nitroxyl (HNO) - the one-electron reduction product of nitric oxide (NO^.^) - has garnered a lot of attention owing to its pharmacological properties that are quite dissimilar from those exhibited by its sibling NO^.^ or other RNS such as nitrite/nitrate. HNO uniqueness stands out especially in the cardiovascular system, where it exerts positive effects on contractility and relaxation, while inducing venous and arterial dilation. These benefits are preserved in failing hearts that harbour altered redox conditions and perturbed signalling pathways, such as cAMP/PKA. In fact, there are, at least, four biochemical properties that single HNO out from other modulators of myocardial function, justifying its potential both as a signalling molecule and therapeutic option. They are its elective, and likely selective thiophilic nature, inertness towards ROS, modest reactivity with molecular O_2_, and likely the fact that HNO does not interfere with other post-translational modifications, such as phosphorylation. Accordingly, HNO donors are currently evaluated for safety and efficacy in patients with acute decompensated heart failure (ADHF). Notwithstanding, many key questions still surround HNO biology and pharmacology. First and foremost, it is still unclear under which conditions, and where HNO is formed in the human body (although it is a major gas in the space!). Equally unknown is what mechanisms eventually cease its signalling, along with the exact chemical nature of HNO-induced modifications that pave the way to either signalling or detrimental effects. Finally, much remains to be discovered in terms of additional HNO biological actions that may dovetail nicely with already established effects in the circulation as well as in other compartments. Here, major “knowns” and “unknowns” about HNO biology and HNO donor pharmacology will be discussed, and the HNO therapeutic portfolio for ADHF will be compared to current mainstay therapies.

***Competing interest***

*Dr. Nazareno Paolocci is scientific founder and stock-holder at Cardioxyl Pharmaceuticals, Inc./Bristol-Myers Squibb.*

## A18 Redox regulation of G-kinase

### Philip Eaton^1^ (philip.eaton@kcl.ac.uk)

#### ^1^King’s College London, Cardiovascular Division, The Rayne Institute, St Thomas’ Hospital, London, UK

Protein kinase G (PKG) is subject to complex redox regulation involving several modes of oxidation, including interprotein disulfide formation at C42. The C42 disulfide homodimer is associated with targeting and activation of PKG Iα, which significantly contributes to vasodilation and blood pressure lowering in response to exogenously applied or endogenously generated oxidants. Interdisulfide-PKG Iα levels were found to increase in the mouse heart during diastolic stretch, consistent with evidence that oxidants are produced during myocardial relaxation at this time. Interdisulfide-PKG Iα selectively targets and phosphorylates phospholamban at S16, which activates the sarcoplasmic reticulum (SR) Ca^2+^ ATPase 2a (SERCA2a) to enhance sequestration of calcium into the sarcoplasmic reticulum. Consistent with this C42S PKGIα knock-in (KI) mice that are resistant to kinase oxidation to the interdisulfide state are hypertensive and have diastolic dysfunction as evidenced by echocardiography and left ventricular Pressure-Volume catheter analysis. Thus, it is evident that PKG Iα interprotein disulfide formation occurs during myocardial stretch, and without this mechanism the hearts have impaired relaxation during diastole. Given the role for interdisulfide-PKG Iα in blood pressure-lowering, we assembled and screened a ‘soft-electrophile’ compound library for molecules that would mimic or induce oxidation of the kinase. We identified a compound which we have called G1 which induces vasodilation of isolated arterial vessels from wild type mice, whereas the impact on vessels from KI was significantly deficient. G1 also lowered blood pressure in an angiotensin II-induced hypertension model in wild type, but not KI, mice. This provides proof-of-principle for a new class of anti-hypertensive drugs, which conceptionally may also have value in the treatment of diastolic heart failure.

## A19 Structural Basis of Analog Specificity in PKG I and II

### James C. Campbell^1,2^, Philipp Henning^3^, Eugen Franz^3^, Banumathi Sankaran^4^, Friedrich W. Herberg^4^, Choel Kim^1,2,5,^

#### ^1^Structural and Computational Biology and Molecular Biophysics Program, Baylor College of Medicine, Houston, TX, USA; ^2^Department of Pharmacology, Baylor College of Medicine, Houston, TX, USA; ^3^Department of Biochemistry, University of Kassel, Kassel, Hesse, Germany; ^4^Berkeley Center for Structural Biology, Lawrence Berkeley National Laboratory, Berkeley, CA, USA; ^5^Verna and Marrs McLean Department of Biochemistry and Molecular Biology, Baylor College of Medicine, Houston, TX, USA

##### **Correspondence:** Choel Kim (ckim@bcm.edu)

**Background:**

As a primary receptor of cGMP in mammalian cells, PKG is a central mediator of the NO-cGMP signaling pathway that regulates crucial physiological processes [1-3]. These include smooth muscle tone, bone growth, nociception, and memory formation. Two types of PKGs exist, PKG I and II. They display distinct subcellular localization, tissue expression, and substrates suggesting their non-redundant cellular functions [4].

Cyclic GMP analogs, 8-Br-cGMP, 8-pCPT-cGMP, and PET-cGMP, have been widely used for characterizing cellular functions of PKG I and II isotypes [5, 6]. However, interpreting results obtained using these analogs has been difficult due to their low isotype specificity. Additionally, the regulatory (R)-domain of each isotype has two binding sites with different cGMP and analog binding characteristics [7], making understanding the molecular basis for isotype specificity of these compounds even more challenging.

**Results:**

To determine isotype specificity of cGMP analogs and their structural basis, we generated the full-length regulatory domains of PKG I and II with each binding site disabled, determined their affinities for these analogs, and obtain co-crystal structures of both isotypes bound with cGMP analogs. To disable each site for cGMP binding, we replaced a conserved glycine within each cGMP binding pocket (G182 and G306 in PKG I and G232 and G356 in PKG II, respectively) with a glutamate. These mutations are predicted to cause steric hindrance and charge repulsion with the negatively charged cyclic phosphate moiety.

Our affinity and activation measurements using competitive surface plasmon resonance and microfluidic mobility-shift assay show that PET-cGMP and 8-pCPT-cGMP are ~40-50 fold selective in binding and ~10 fold in activating against each isotype, whereas 8-Br-cGMP is only ~10 fold selective in binding and ~4 fold in activating PKG II. 8-Br-cGMP and 8-pCPT-cGMP similarly bind and activate PKG I compared to cGMP (Tables 2). In contrast, 8-Br-cGMP and 8-pCPT-cGMP show gradual increases in both EC_50_ and *K*_A_ values for PKG II. Unexpectedly, PET-cGMP binds and activates PKG II with similar potencies as cGMP, providing little selectivity against PKG II (Tables 2)

**Table 2:** **(Abstract A19).** Affinity and activation constants measurements of PKG I and II for cGMP and analogs

Affinity Measurements of PKG I and II Regulatory Dimers				
PKG Iβ				
*h*PKG Iβ 1-351	cGMP	8-Br-cGMP	8-pCPT-cGMP	PET-cGMP
WT	163±6 nM (2)	222±4 nM (2)	212±20 nM (3)	3.8±0.4 nM (2)
G182E	328±6 nM (2)	765±25 nM (2)	206±18 nM (2)	5.8±0.4 nM (2)
G306E	4.2±0.1 nM (3)	< 2 nM (2)	7.7±0.4 nM (2)	<< 2 nM (2)
**PKG II**				
*h*PKG II 41-418	cGMP	8-Br-cGMP	8-pCPT-cGMP	PET-cGMP
WT	96±5 nM (3)	20±2 nM (3)	5±1 nM (3)	193±10 nM (3)
G232E	184±0.1 nM (2)	11±1 nM (2)	116±8 nM (2)	1963±12 nM (2)
G356E	50±3 nM (3)	30±2 nM (2)	≤ 1.1±0.3 nM (2)	5.3±0.3 nM (2)
Activation Constant Measurements of PKG I and II Full Lengths				
	cGMP	8-Br-cGMP	8-pCPT-cGMP	PET-cGMP
*h*PKG Iβ 5-678	370±13 nM (4)	206±13 nM (3)	249±17 nM (3)	18±2 nM (3)
*h*PKG II 40-762	257±11 nM (3)	58±5 nM (3)	22±3 nM (4)	225±13 nM (3)

Footnote:

EC_50_ and *K*_A_ values were measured using competitive surface plasmon resonance and microfluidic mobility shift assay. (n) = Number of measurements.

**Fig. 1:**
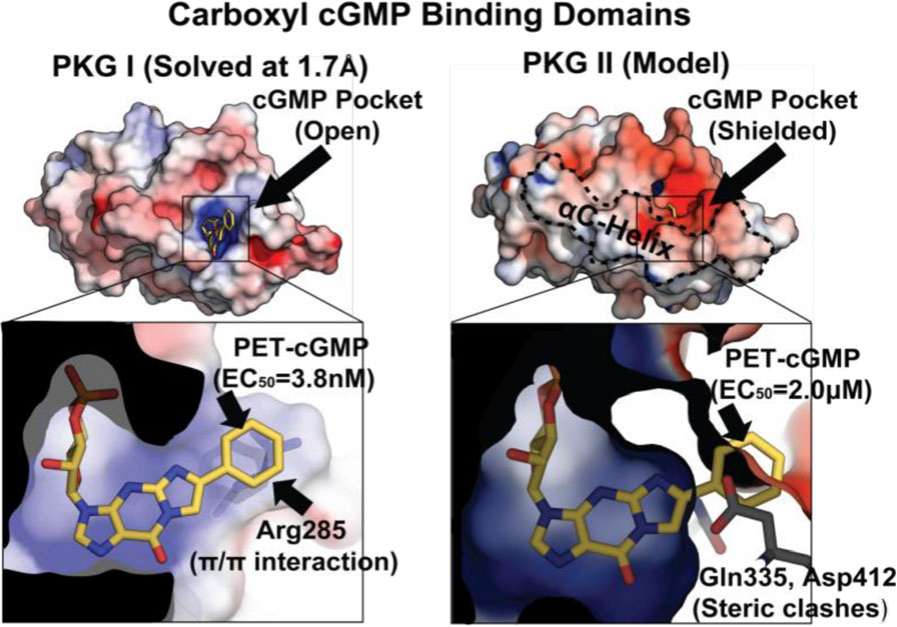
**(Abstract A19).** The crystal structures of carboxyl domains of PKG I and II explain high selectivity of PET-cGMP for PKG I isotype. The crystal structure of the PKG I CNB-B bound with PET-cGMP and the model of the PKG II CNB-B domain docked with PET-cGMP are shown on the top. PET-cGMP was docked onto the PKG II CNB-B domain by superimposing the structures of PKG I CNB-B:PET-cGMP complex and the PKG II CNB-B:cGMP complex (PDB code: 5BV6). Zoomed-in views of the cGMP pockets are shown at the bottom. PKG I shows a unique π/π interaction between Arg285 and the PET-moiety (left) explaining its high selectivity for PET-cGMP. In contrast, Gln335 and Asp412 of PKG II cause steric clashes with the PET-moiety explaining its low affinity (right). The surface is colored according to the contact electrostatic potential calculated with APBS [10]. Positively charged areas are shown in blue and negatively charged areas are in red. The surface corresponding to the αC-helix of PKG II CNB-B is marked with the dotted line

The site-specific mutant data using competitive surface plasmon resonance suggest that the B-sites provide the high preference of PET-cGMP in PKG Iβ and the structures of the PKG I and II CNB-B domains explain why. The crystal structure of PKG I CNB-B domain bound with PET-cGMP shows that PET-cGMP interacts with its more open pocket and forms a unique π/π interaction with Arg285 at β4 (Fig. 1). On the other hand, our structures of PKG II CNB-B show a more shielded pocket due to the C-terminal helix (αC-helix) that provides the most of the cGMP specific contacts (Fig. 1). In particular, our model of the PKG II CNB-B docked with PET-cGMP shows that Gln335 at the analogous position to Arg285 of PKG I and Asp412 at the αC-helix causes steric clashes with the PET moiety reducing its affinity for the B-site [8].

Lastly, the site-specific mutant data also suggest that the A-sites are mainly responsible for the high preference of 8-pCPT-cGMP in PKG II and the crystal structures of the CNB-A domains explain why. Our structures of the CNB-A domains of PKG I and II show that PKG II has a larger β5/β6 hydrophobic pocket that can better accommodate a bulky functional group compared to PKG I, which explains its lower EC_50_ value [8, 9].

**Conclusion:**

Our structural and functional results explain selectivity of these analogs for PKG I and II and provide a starting point for the rational design of isotype selective activators.

*Competing interest*

*The authors declare no conflict of interest.*

**References:**

[1]. Francis SH, Busch JL, Corbin JD, Sibley D: **cGMP-dependent protein kinases and cGMP phosphodiesterases in nitric oxide and cGMP action.** *Pharmacol Rev* 2010, **62:**525-563.

[2]. Hofmann F, Wegener JW: **cGMP-Dependent Protein Kinases (cGK).** In *Guanylate Cyclase and Cyclic GMP: Methods and Protocols.* Edited by Krieg T, Lukowski R. Totowa, NJ: Humana Press; 2013: 17-50

[3]. Schlossmann J, Desch M: **cGK Substrates.** In *cGMP: Generators, Effectors and Therapeutic Implications.* Edited by Schmidt HHHW, Hofmann F, Stasch J-P. Berlin, Heidelberg: Springer Berlin Heidelberg; 2009: 163-193

[4]. Hofmann F, Feil R, Kleppisch T, Schlossmann J: *Function of cGMP-Dependent Protein Kinases as Revealed by Gene Deletion.* 2006.

[5]. Poppe H, Rybalkin SD, Rehmann H, Hinds TR, Tang X-B, Christensen AE, Schwede F, Genieser H-G, Bos JL, Doskeland SO, et al: **Cyclic nucleotide analogs as probes of signaling pathways.** *Nat Meth* 2008, **5:**277-278.

[6]. Schwede F, Maronde E, Genieser H, Jastorff B: **Cyclic nucleotide analogs as biochemical tools and prospective drugs.** *Pharmacol Ther* 2000, **87:**199-226.

[7]. Corbin JD, Ogreid D, Miller JP, Suva RH, Jastorff B, Doskeland SO: **Studies of cGMP analog specificity and function of the two intrasubunit binding sites of cGMP-dependent protein kinase.** *J Biol Chem* 1986, **261:**1208-1214.

[8]. Campbell JC, Kim JJ, Li KY, Huang GY, Reger AS, Matsuda S, Sankaran B, Link TM, Yuasa K, Ladbury JE, et al: **Structural Basis of Cyclic Nucleotide Selectivity in cGMP-Dependent Protein Kinase II.** *Journal of Biological Chemistry* 2016.

[9]. Kim JJ, Casteel DE, Huang G, Kwon TH, Ren RK, Zwart P, Headd JJ, Brown NG, Chow DC, Palzkill T, Kim C: **Co-crystal structures of PKG Ibeta (92-227) with cGMP and cAMP reveal the molecular details of cyclic-nucleotide binding.** *PLoS One* 2011, **6:**e18413.

[10]. Baker NA, Sept D, Joseph S, Holst MJ, McCammon JA: **Electrostatics of nanosystems: application to microtubules and the ribosome.** *Proc Natl Acad Sci U S A* 2001, **98:**10037-10041.

## A20 Insights in the regulation of Mycobacterial protein kinase G by redox changes, phophsporylation and membrane interactions by NMR

### M. Wittwer^1^, Q. Luo^2^, V. Kaila^2^, S. A. Dames^1,3^

#### ^1^Technische Universität München, Department of Chemistry, Biomolecular NMR Spectroscopy, Garching, Germany; ^2^Technische Universität München, Department of Chemistry, Computational Biocatalysis, Garching, Germany; ^3^Institute of Structural Biology, Helmholtz Zentrum München, Neuherberg, Germany

##### **Correspondence:** S. A. Dames (sonja.dames@tum.de)

**Biological background:**

**Fig. 2:**
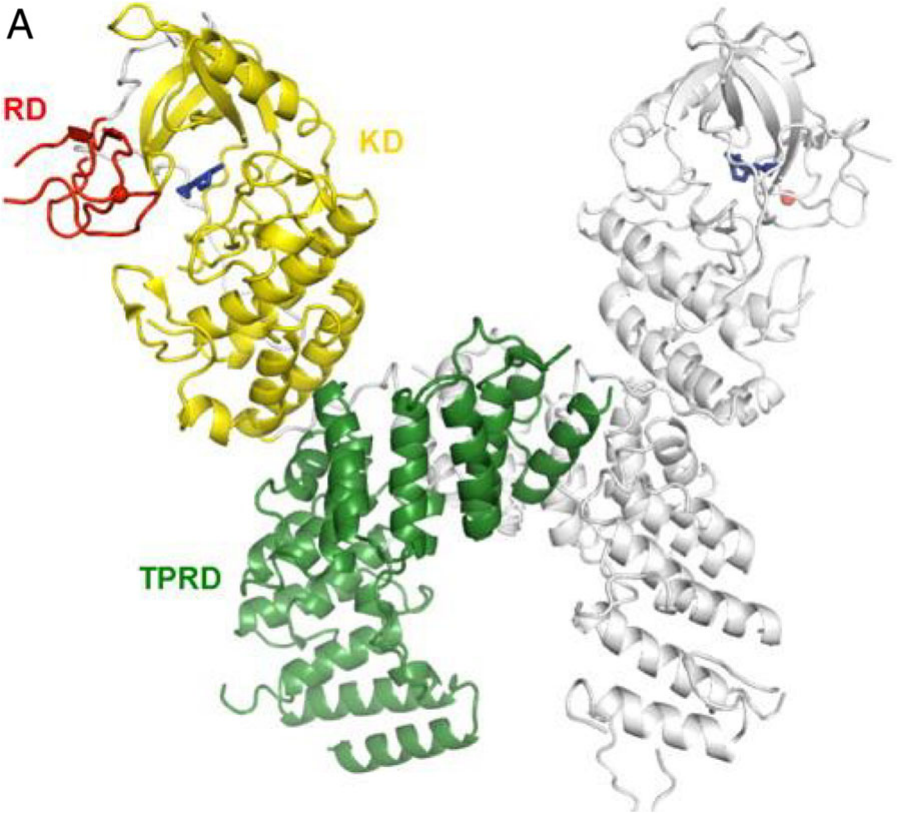
**(Abstract A20).** The crystal structure of *Mycobacterium tuberculosis* protein kinase G from residues 74-750 [1]. The redox-sensitive metal binding motif (RD) is shown in red, the catalytic serine/threonine kinase domain (KD) in complex with a small molecule inhibitor in yellow and blue, respectively, and the C-terminal tetratricopeptide repeat domain (TPRD) in green

*Mycobacterium tuberculosis* escapes killing in human macrophages by secreting protein kinase G (PknG), which intercepts host signaling to prevent the fusion of the phagosome engulfing the mycobacteria with the lysosome. The N-terminal ~75 residues were predicted to show no regulatory secondary structure (NORS, not present in the crystal structure shown in Fig. 2) but to harbor the major *in vivo* phosphorylation site (T63) and to play a role for PknG regulation by autophosphorylation *in trans*. The following rubredoxin-like metal-binding motif (RD, ~74–147, Fig. 2) makes tight interactions with the catalytic domain (~148–420) and mediates PknG redox regulation. Deletions or mutations in the NORS or the redox-sensitive RD significantly decrease PknG survival function.

**Results and Conclusion:**

Here, we present nuclear magnetic resonance (NMR) spectroscopy, *in vitro* kinase assay, and molecular dynamics (MD) simulation data that provide novel insights in the regulatory roles of the NORS and the RD. The NORS region is rather dynamic and appears indeed to be natively disordered. In agreement with published data, we observe autophosphorylation only if the NORS region is present and thus in the NORS region. Phosphorylation in the NORS results only in local conformational changes and does not induce interactions with the following RD. In the crystal structure (Fig. 2), the reduced, metal bound RD makes tight interactions with the catalytic domain. Based on NMR structural data, it can also fold in its absence. The combined NMR-, MD-, and kinase assay data further suggest that oxidation-induced unfolding of the RD regulates substrate access to the catalytic domain and thereby PknG function under different redox conditions, e.g. if exposed to increased levels of reactive oxidative species (ROS) in host macrophages [2]. We further analyzed the interactions of the RD with membrane mimetics. Both, the reduced, metal bound and the oxidized unfolded RD, can interact with micelles and bicelles, but not liposomes. This may play a role for the observed membrane localization of PknG.

**Competing interest**

*The authors declare that they have no conflicts of interest with the contents of this article.*

**Funding**

*This work was supported by grants from the German Research Foundation (DFG) to SAD (SFB1035, project B04) and VRIK (SFB1035, project B12). SAD acknowledges further financial support from the TUM diversity and talent management office and the Helmholtz portfolio theme ‘metabolic dysfunction and common disease’ of the Helmholtz Zentrum München. QL acknowledges the China Scholarship Council (CSC) for funding. Computer resources were provided in part by the Gauss Centre for Supercomputing/Leibniz Supercomputing Centre (grant: pr84pa to VRIK).*

**References:**

[1]. N Scherr, S Honnappa, G Kunz, P Mueller, R Jayachandran, F Winkler, J Pieters, MO Steinmetz: **Structural basis for the specific inhibition of protein kinase G, a virulence factor of Mycobacterium tuberculosis**. *Proceedings of the National Academy of Sciences of the United States of America 2007,* **104**: 12151-12156

[2]. M Wittwer, Q Luo, VR Kaila VR, SA Dames SA: **Oxidative Unfolding of the Rubredoxin Domain and the Natively Disordered N-terminal Region Regulate the Catalytic Activity of Mycobacterium tuberculosis Protein Kinase G**, J Biol Chem. 2016, **291(53)**:27062-27072

## A21 Employing chemical genetics to determine the cellular targets for G-kinase in malaria

### Andrew Tobin, Mahmood Alam

#### Institute of Molecular Cell and Systems Biology, University of Glasgow, Glasgow, UK

##### **Correspondence:** Andrew Tobin **(**Andrew.Tobin@glasgow.ac.uk**)**

Our laboratory have determined that of the 80-90 eukaryotic protein kinases expressed in the malaria parasite kinome nearly half are essential for the blood stage survival of the most virulent strain of human malaria, P. falciparum. One of these essential protein kinases is the cGMP-dependent protein kinase, PfPKG. To determine the cellular substrates for PfPKG we employed a novel chemical genetic approach that takes advantage of a change in the gate keeper residue of PfPKG that renders the kinase insensitive to a inhibitor called compound 1. Using this approach in combination with quantitative global phosphoproteomics, the phosphorylation sites on 69 proteins that are direct or indirect cellular targets for PfPKG were identified. These PfPKG targets included proteins involved in cell signalling, proteolysis, gene regulation, protein export and ion and protein transport, indicating that cGMP/PfPKG signalling plays a central role in a number of core parasite processes. We also show that PfPKG activity is required for parasite invasion. This correlates with the finding that the calcium-dependent protein kinase, PfCDPK1, is phosphorylated by PfPKG, as are components of the actomyosin complex, in a manner that provides a mechanistic insight into the essential role of PfPKG in parasite egress and invasion.

## A22 Oxidation of cGMP-dependent protein kinase Iα in the lung during chronic hypoxia mediates an endogenous adaptation to pulmonary hypertension

### Olena Rudyk^1^, Susanne Krasemann^2^, Kristin Hartmann^2^, Oleksandra Prysyazhna^1^, Min Zhang^1^, Lan Zhao^3^, Astrid Weiss^4^, Ralph Schermuly^4^, Philip Eaton^1^

#### ^1^BHF Centre of Research Excellence, King’s College London, London, SE1 7EH, UK; ^2^Core Facility for Mouse Pathology, University Medical Centre Hamburg-Eppendorf, Hamburg 20246, Germany; ^3^Faculty of Medicine, Department of Medicine, Imperial College London, London, SW7 2AZ, UK; ^4^Excellence Cluster Cardio-Pulmonary System, Universities of Giessen and Marburg Lung Centre Member of the German Lung Canter Justus-Liebig-University Giessen, Giessen, Germany

##### **Correspondence:** Olena Rudyk **(**olena.rudyk@kcl.ac.uk**)**

**Background:**

Pulmonary hypertension (PH) is a disease of the vasculature of the airways resulting in vasoconstriction and arterial remodelling, eventually leading to right ventricle failure and death. cGMP-dependent protein kinase (PKG) I knock-out mice develop spontaneous PH, while PKG I protein expression is paradoxically upregulated during hypoxic PH. Previous work from this laboratory has shown PKG Iα is susceptible to oxidation, forming a disulfide homodimer associated with targeting and activation of the kinase that mediates vasodilation and blood pressure lowering. During acute hypoxia, pulmonary cells become pro-reducing – which may be anticipated to reduce the amount of disulfide-PKG Iα and so contribute to acute hypoxic pulmonary vasoconstriction. In the present study, we investigated the redox state of pulmonary PKG Iα during chronic hypoxia and its potential role in pathogenesis of hypoxic PH.

Results and Discussion:

Mice were subjected to hypoxia (10% O_2_), which caused oxidation of lung PKG Iα as determined at 3 or 28 day time points compared to basal normoxic levels. The H_2_O_2_-producing enzymes NADPH oxidase 4 (NOX4) and extracellular superoxide dismutase (SOD3) were also increased in the lungs as measured at the 28 day time point, thus potentially contributing to PKG Iα oxidation. In addition, the vascular isoform of cystathionine gamma-lyase (CSE), which produces the vasorelaxant H_2_S, was also upregulated at both time points of hypoxia. These observations are consistent with PKG Iα oxidation being mediated by H_2_O_2_-induced oxidation of H_2_S, which generates pro-oxidising polysulfide species. Interestingly, we observed increased PKG Iα oxidation, as well as NOX4 and CSE upregulation in lungs from patients with pulmonary arterial hypertension. This suggests that kinase oxidation pathway may be important in the etiology of PH in humans.

To dissect the potential role of oxidised PKG Iα in hypoxic PH we utilised redox dead Cys42Ser PKG Iα knock-in (KI) mice which are resistant to oxidation. We found that the KI mice had increased pulmonary vascular resistance, elevated pulmonary myosin light chain phosphorylation, as well as potentiated right ventricular hypertrophy and higher right ventricular pressure after 4 weeks of chronic exposure to hypoxia, as compared to their WT littermates. These observations were consistent with U-46619-constricted pulmonary vessels from KI mice having impaired vasodilatory responses to H_2_O_2_ compared to WT under normoxic condition, despite equal maximal constriction to the pressor agonist. Our finding suggests that chronic PKG Iα oxidation, as well as perhaps increased PKG expression, serves an important novel intrinsic adaptive mechanism to offset increased pulmonary pressure in PH and thus reduce RV after-load and so limit progression to right heart failure. Chronic pharmacological inhibition of CSE with propargylglycine (50 mg/kg/day) potentiated RV pressure and hypertrophy, as well as attenuated PKG Iα oxidation in the lung after 2 weeks of chronic hypoxic exposure. In contrast, chronic treatment with the H_2_S donor sodium hydrosulfide (23 mg/kg/day) or potassium polysulfide (2 mg/kg/day) prevented excessive RV pressure and hypertrophy after 2 weeks of hypoxia. Preliminary data from ongoing experiments suggest that this protection provided to WT mice by H_2_S or its oxidised variants was not afforded to the Cys42Ser PKG Iα KI.

An unbiased microarray transcriptomic screen revealed an upregulation of pro-growth, extracellular matrix remodelling and endothelial to mesenchymal transition cellular signalling pathways in response to early hypoxia (day 3) in the lungs of the redox-dead KI mice compared to WT. Consequently, the KI mice showed excessive muscularisation of small pulmonary vessels and enhanced transition of pulmonary endothelial cells to myofibroblasts after 4 weeks of chronic hypoxia. Given the important role of PKG I in cell growth and cancer, these data suggest that hypoxia-induced PKG Iα oxidation may prevent pulmonary vascular smooth muscle cell proliferation and/or endothelial to mesenchymal cell transition, in addition to its pressure-lowering role.

**Conclusion:**

We conclude that hypoxia-induced NOX4, SOD3 and CSE upregulation may underlie PKG Iα oxidation in this scenario and thus could mediate endogenous adaptation to PH by inducing phosphorylation of target proteins involved in lowering pulmonary pressure, resulting in reduced RV afterload in the setting of PH. This mechanism could also be relevant to other situations in which occurs PH, including PAH in human. The redox dead PKG Iα KI mice lack this protective mechanism and therefore have an exacerbated hypoxic PH phenotype. Interventions inducing PKG Iα oxidation in the lung, such as H_2_S donors or drugs that induce the selective oxidation of PKG Iα, could be beneficial in the treatment and management of PAH.

***Competing interest***

*The authors declare no competing financial interests or potential conflict of interests.*

## A23 C-type natriuretic peptide regulates cardiac structure & function

### Amie J Moyes^1^, Sandy M Chu^1^, Reshma S Baliga^1^ and Adrian J Hobbs^1^

#### ^1^William Harvey Research Institute, Barts & The London School of Medicine, QMUL, London, United Kingdom

##### **Correspondence:** Amie J Moyes (a.j.moyes@qmul.ac.uk)

**Background:**

Endothelium-derived C-type natriuretic peptide (CNP) plays a fundamental role in regulating vascular homeostasis by controlling arterial tone, blood pressure, leukocyte flux, platelet reactivity and the integrity of the vessel wall [1]. However, a physiological role for endogenous CNP in the heart remains to be established. Therefore, we have utilised two novel mouse strains with endothelium or cardiomyocyte -specific deletion of CNP to determine if the peptide modulates heart function under basal conditions and during cardiac stress.

**Materials & Methods:**

Blood pressure and electrocardiogram (ECG) were assessed by radiotelemetry. A Langendorff heart model was used to study coronary vascular reactivity and ischemia-reperfusion (I/R) injury *ex vivo*. Echocardiography was performed to determine cardiac function at baseline and following pressure overload (abdominal aortic constriction [AAC]; 6 weeks) -induced left ventricular hypertrophy/heart failure. Following AAC, cardiomyocyte hypertrophy and interstitial fibrosis were determined using immunohistochemistry and qPCR. A subset of experiments were also repeated in mice with global deletion of natriuretic peptide receptor C (NPR-C) to delineate the signalling pathway involved in mediating any cardiac effects of CNP.

**Results:**

Hearts from endothelium-specific CNP knockout (ecCNP KO) mice exhibited attenuated responses to the vasodilators bradykinin and acetylcholine compared to wildtype (WT) littermates. Shear-stress induced coronary dilatation (i.e. reactive hyperaemia) was also blunted in ecCNP KO. Larger myocardial infarct sizes and poorer recovery of left ventricular contractility were observed in hearts from mice lacking cardiomyocyte-derived CNP (cmCNP KO) following IR injury. Similar results were found in NPR-C KO mice but not ecCNP KO hearts. Under basal conditions heart rate, blood pressure and ECG parameters in cmCNP KO mice were not altered. However, during pressure overload-induced heart failure cmCNP KO mice exhibited greater cardiac dysfunction and fibrosis than WT littermates; a similar phenotype was apparent in NPR-C KO, but not ecCNP KO, animals subjected to AAC. Furthermore, infusion of CNP via an osmotic mini-pump reversed cardiac dysfunction following AAC in WT animals, but had no effect in NPR-C KO mice.

**Conclusion:**

These data suggest that both endothelial and cardiomyocyte -derived CNP play distinct but important roles in the heart, governing coronary vascular tone and the response to pressure overload. These protective functions are mediated, at least in part, via activation of NPR-C.

**Competing interest**

*The authors declare no conflicts of interest.*

**Reference:**

[1] Moyes AJ et al. 2014. **Endothelial C-type natriuretic peptide maintains vascular homeostasis.** *J Clin Invest.* 124:4039-51.

## A24 Gene therapy for *CNGA3*-linked achromatospia: from mouse models to clinical trials

### Stylianos Michalakis^1^, Regine Mühlfriedel^2^, Christian Schön^1^, Dominik M Fischer^3^, Barbara Wilhelm^4^, Ditta Zobor^3^, Susanne Kohl^2^, Tobias Peters^4^, Eberhart Zrenner^2,3^, Karl Ulrich Bartz-Schmidt^3^, Marius Ueffing^2^, Bernd Wissinger^2^, Mathias Seeliger^2^, Martin Biel^1^, RD-CURE consortium

#### ^1^Center for Integrated Protein Science Munich CiPSM at the Department of Pharmacy – Center for Drug Research, Ludwig-Maximilians-Universität München, Munich, Germany; ^2^Institute for Ophthalmic Research, Centre for Ophthalmology, University of Tübingen, Tübingen, Germany; ^3^University Eye Hospital, Centre for Ophthalmology Tübingen, University of Tübingen, Tübingen, Germany; ^4^STZ eyetrial at the Centre for Ophthalmology, University of Tübingen, Tübingen, Germany

##### **Correspondence:** Stylianos Michalakis (michalakis@lmu.de)

**Clinical background:**

**Fig. 3:**
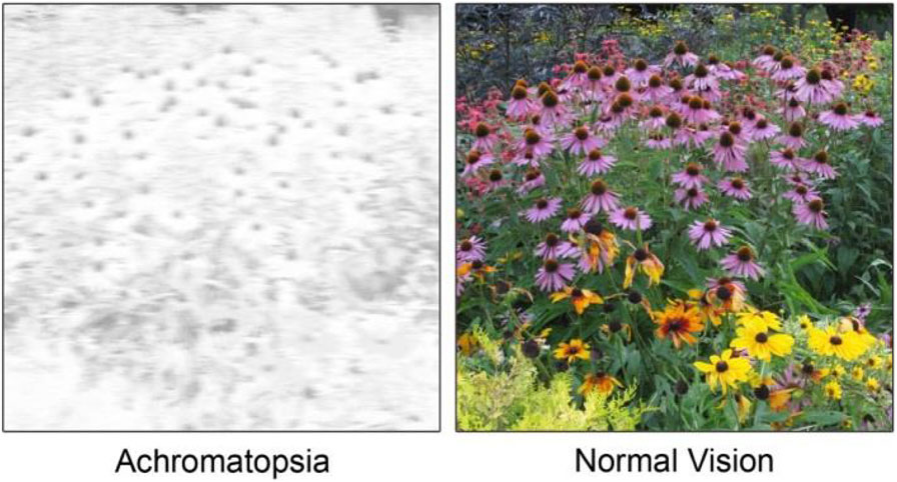
**(Abstract A24).** Simulation of daylight vision in Achromatopsia patients. Patients with Achromatopsia are unable to distinguish colors, have markedly reduced visual acuity and are hypersensitive to ambient light (left panel). The same image of a flower meadow as seen with normal vision at daylight (left panel)

Achromatopsia (ACHM) is a genetically and clinically well-defined inherited retinal disorder. Patients with ACHM suffer from severely impaired daylight vision, characterized by poor visual acuity, photophobia, nystagmus (involuntary rapid eye movements), and lack of the ability to discriminate colors (Fig. 3). Currently, six disease genes have been identified including the two genes encoding the cone-specific cyclic nucleotide-gated channel subunits CNGA3 and CNGB3.

**Results:**

We developed a recombinant adeno-associated virus (AAV) vector-mediated gene supplementation therapy for the treatment of CNGA3-linked ACHM (ACHM2). The vector expresses full length human CNGA3 under control of the human, cone-specific cone arrestin promoter and was packaged with AAV8 capsid. The resulting vector (rAAV8.CNGA3) was tested for efficacy in the Cnga3 knockout (KO) mouse model of ACHM2. Toxicity and biodistribution was assessed in non-human primates (NHP). rAAV8.CNGA3 delivered into the subretinal space of Cnga3 KO led to efficient and stable CNGA3 transgene expression and biological activity as determined by immunohistochemistry and electroretinography, respectively. Up to 1x10^12^ total vector genomes (vg) have proven save when delivered into the subretinal space of NHPs with only limited biodistribution and shadding and minimal signs of inflammation. An interventional phase I/II clinical trial (NCT02610582) was initiated focusing on safety and efficacy of a single subretinal injection of rAAV8.CNGA3 in patients with ACHM2 at three different doses: 1x10^10^, 5x10^10^, and 1x10^11^ total vg.

**Conclusions:**

Although the approach was targeting the central retina and involved temporal detachment of the fovea/macula, the treatment has proven to be safe, was well tolerated and did not results in any clinically apparent inflammation or test item related events. Preliminary clinical data will be discussed.

**Funding**

*This work was supported by the Tistou and Charlotte Kerstan Foundation.*

***Competing interest***

*MB, SM and MS are inventors on related patents. MB and SM are founders of ViGeneron GmbH. EZ is founder of EyeServ GmbH.*

## A25 Novel role of PKG in Protein Quality Control by Regulating CHIP

### Mark J. Ranek^1^, Kristen M. Kokkonen^1^, Dong I. Lee^1^, Ronald J. Holewinski^2^, Vineet Agrawal^1^, Cornelia Virus^3^, Donté A. Stevens^3^, Masayuki Sasaki^1^, Huaqun Zhang^4^, Mathew M. Mannion^4^, Peter P. Rainer^1^, Richard C. Page^4^, Jonathan C. Schisler^3^, Jennifer E. Van Eyk^2^, Monte S. Willis^3^, and David A. Kass^1^

#### ^1^Division of Cardiology, Johns Hopkins Medical Institutions, Baltimore, MD 21205, USA; ^2^Heart Institute and Advanced Clinical Biosystems Research Institute, Cedar Sinai Medical Center, 8700 Beverly Blvd, AHSP A9229 Los Angeles, California 90048, USA; ^3^McAllister Heart Institute, Division of Cardiology, UNC, Chapel Hill, NC, USA; ^4^Department of Chemistry and Biochemistry, Miami University, Oxford, OH 45056, USA

##### **Correspondence:** David A. Kass (dkass@jhmi.edu)

The accumulation of damaged/misfolded and consequently ubiquitinated proteins that are insufficiently cleared by the ubiquitin proteasome system (UPS) contributes to many diseases. However, therapeutic methods to enhance the UPS and counter proteotoxicity are lacking. Cardiac infarction is an example of a disorder where ubiquinated protein accumulation (UPA) is thought to play a pathophysiological role. In studies employing a model in which PKG activation is suppressed in vivo or in vitro, we found this accumulation and attendant myocardial or myocyte toxicity worsened. Activating PKG in a similar setting was beneficial. An unbiased cGMP-stimulated phosphoproteome analysis in adult myocytes revealed Ser20 on carboxyl terminus of the Hsc70-interacting protein (CHIP) was a novel target of PKG activation. We found suppressing PKG activation results in reduced CHIP expression in the myocardium and in myocytes subjected to simulated ischemia. Using gain (S20E) and loss (S20A) of function phospho-mutants, we found S20E-CHIP mimicked PKG protection, reducing UPA and cytotoxicity with ischemia. The opposite occurred with S20A-CHIP. Intriguing, if S20A was expressed, PKG activation was unable to rescue the worsened phenotype, even though it can still act to blunt other signalling pathways including enhancement of proteasome protease activity. The molecular consequence of S20 phosphorylation was an increase of CHIP binding to its cognate chaperone Hsc70, and stabilization of post-transcriptional CHIP protein levels. Depressing PKG activity (or using the S20A mutant) dissociated CHIP from Hsc70, reduced its protein expression levels, and compromised its capacity to enhance protein quality control. These data identify PKG-CHIP interactions that control UPS activity, and provide a new therapeutic avenue to treat diseases with compromised protein quality control.

## A26 PDE/cAMP regulation of cardiac hypertrophy

### Manuela Zaccolo (manuela.zaccolo@dpag.ox.ac.uk)

#### Department of Pyhisology, Anatomy and Genetics, University of Oxford, Oxford, UK

In addition to its role as regulator of the chronotropic, inotropic and lusitropic response to catecholamines, cAMP affects multiple other functions including, among others, cell growth, metabolism and death. This complex functional role is achieved via modulation of ion fluxes at membranes and of myofilament sensitivity to Ca^2+^ as well as via regulation of transcription factors and a variety of enzymes and other targets. A key question remains how coordination is achieved among the complex cAMP signalling networks. In recent years we[1-5] and others[6-8] have demonstrated that cAMP signalling is compartmentalised. Compartmentalised signalling allows individual GPCRs to generate distinct cAMP pools that, in turn, activate defined subsets of localized PKA that are tethered in proximity to specific targets via binding to A kinase anchoring proteins (AKAPs). Posphodiesterases (PDEs), a superfamily of enzymes that degrade cAMP and that includes more than 50 isoforms presenting unique regulation and subcellular localisation features, play a key role in the spatial regulation of cAMP propagation, and regulate cAMP levels within individual compartments. Thus, displacement of individual PDE isoforms from their subcellular anchor site results in local elevation of cAMP[5].

Compartmentalisation of cAMP signalling has important implications for cardiac physiology and pathophysiology[6]. We have recently demonstrated, for example, that inhibition of PDE2A, but not inhibition of PDE3 or PDE4, results in anti-hypertrophic effects both *in vitro* and *in vivo*[9]*. The c*ompartmentalised nature of cAMP signalling prompts the idea that with a detailed understanding of the organization, regulation and function of individual cAMP compartments it may be possible to target individual cAMP pools, rather than global intracellular cAMP levels, in order to achieve greater therapeutic efficacy and specificity[7].

**References:**

[1]. Zaccolo, M. *et al.* **A genetically encoded, fluorescent indicator for cyclic AMP in living cells.** *Nature cell biology* **2**, 25-29 (2000).

[2]. Zaccolo, M. & Pozzan, T. **Discrete microdomains with high concentration of cAMP in stimulated rat neonatal cardiac myocytes.** *Science* **295**, 1711-1715 (2002).

[3]. Mongillo, M. *et al.* **Fluorescence resonance energy transfer-based analysis of cAMP dynamics in live neonatal rat cardiac myocytes reveals distinct functions of compartmentalized phosphodiesterases.** *Circ Res* **95**, 67-75 (2004).

[4]. Mongillo, M. *et al.* **Compartmentalized phosphodiesterase-2 activity blunts beta-adrenergic cardiac inotropy via an NO/cGMP-dependent pathway.** *Circ Res* **98**, 226-234 (2006).

[5]. Di Benedetto, G. *et al.* **Protein kinase A type I and type II define distinct intracellular signaling compartments.** *Circ Res* **103**, 836-844 (2008).

[6]. Jurevicius, J. & Fischmeister, R. **cAMP compartmentation is responsible for a local activation of cardiac Ca2+ channels by beta-adrenergic agonists.** *Proc Natl Acad Sci U S A* **93**, 295-299 (1996).

[7]. Nikolaev, V. O. *et al.* **Beta2-adrenergic receptor redistribution in heart failure changes cAMP compartmentation.** *Science* **327**, 1653-1657 (2010).

[8]. Dodge-Kafka, K. L. *et al.* **The protein kinase A anchoring protein mAKAP coordinates two integrated cAMP effector pathways.** *Nature* **437**, 574-578 (2005).

[9]. Zoccarato, A. *et al.* **Cardiac Hypertrophy Is Inhibited by a Local Pool of cAMP Regulated by Phosphodiesterase 2.** *Circ Res* **117**, 707-719, doi:10.1161/CIRCRESAHA.114.305892 (2015).

## A27 Real-time measurements of cGMP in cardiac, neuronal and renal cells

### Michael Russwurm^1^, Jan Giesen^1^, Corina Russwurm^1^ Ernst-Martin Füchtbauer^2^, Doris Koesling^1^

#### ^1^Institute for Pharmacology and Toxicology, Medical Faculty, Ruhr-University Bochum, 44780 Bochum, Germany; ^2^Department of Molecular Biology and Genetics - Molecular Cell and Developmental Biology, Aarhus University, C.F. Møllers Allé 3, 8000 Aarhus C, Denmark

##### **Correspondence:** Michael Russwurm (michael.russwurm@ruhr-uni-bochum.de)

The second messenger cGMP serves diverse functions in the cardiovascular and neuronal systems. FRET-based cGMP indicators derived from the cGMP-binding domains of the cGMP-dependent protein kinase I can be used to visualize cGMP signals in primary cells. However, expression of FRET-based cGMP indicators in primary cells is challenging. Therefore, we generated a knock-in mouse line with stable and ubiquitous expression of a FRET-based cGMP indicator (cGi500). This indicator with an EC50 for cGMP of 500 nM allows for the detection of cGMP concentrations between 100 nM and 3 μM.

Using these mice, we analysed cGMP signals in primary neurons, cardiac cells (myocytes and fibroblasts) and renal glomeruli. In cardiac cells and renal glomeruli, nitric oxide- and natriuretic peptide-induced signals were compared and the phosphodiesterases responsible for cGMP degradation were identified. In hippocampal and cortical neurons, analysis of cGMP signals induced by glutamatergic agonists revealed that not only NMDA but also AMPA is able to increase cGMP via endogenous nitric oxide formation.

## A28 Changes in Cardiomyocyte cGMP Dynamics during Hypoxia/Reoxygenation

### Nadja I. Bork^1^, Viacheslav O. Nikolaev^1^

#### ^1^Institute of Experimental Cardiovascular Research, Center for Experimental Medicine, University Medical Center Hamburg-Eppendorf, Hamburg, Germany

##### **Correspondence:** Nadja I. Bork **(**n.bork@uke.de**)**

**Introduction:**

Cyclic guanosine 3´5´-monophosphate (cGMP) plays a crucial role in the regulation of multiple physiological processes including the cardiovascular system [1]. As a consequence of hypoxia in the heart, maladaptive signaling cascades are activated that can result in cardiac damage and finally lead to heart failure [2, 3]. However, until now, cellular responses to hypoxia/reoxygenation (H/R) are still incompletely understood and little is known about cGMP dynamics in the context of H/R.

**Aims and methods:**

It was the aim of this project to study the effects of H/R on cGMP dynamics in mammalian cardiomyocytes. Therefore transgenic mice with cardiomyocyte-specific expression of the cytosolic Förster-resonance energy transfer (FRET)-based cGMP sensor red cGES-DE5 [4] were used. We performed FRET measurements in single adult cardiomyocytes exposed to H/R and additionally used a Langendorff system for FRET measurements in whole heart.

**Results:**

In single adult cardiomyocytes exposed to H/R, basal cGMP levels were increased. This increase was generated during hypoxia and was maintained during reoxygenation. After H/R PDE3 protein expression as well as activity were significantly downregulated, whereas RNA expression level of PDE3 was not significantly changed. At the same time, protein levels of soluble guanylyl cyclase β-subunit showed a tendency towards downregulation during H/R.

Our whole heart measurements indicate that there is also an influence of other cell-types on cGMP dynamics or on cGMP cell-cell transfer since we could show that cGMP levels increase during anoxia and decrease during reoxygenation.

**Conclusion:**

In conclusion, we found an increase of intracellular cGMP levels in hypoxia.

The development of FRET-based cGMP measurements in single cardiomyocytes and whole hearts in the context of H/R should help to distinguish between direct protective effects on cardiomyocytes and indirect mechanisms such as cell-cell interactions in cGMP signaling during and after H/R. This offers great opportunities to dissect the molecular mechanism of cGMP signaling regulation during ischemic injury.

***Competing interest***

*We have no conflict of interest to declare. We have no commercial associations that might pose a conflict of interest with the submitted abstract.*

**Funding**

*DFG FOR 2060; Gertraud und Heinz Rose-Stiftung.*

**References:**

[1] EJ Tsai and DA Kass: **Cyclic GMP signaling in cardiovascular pathophysiology and therapeutics.** *Pharmacol Ther* 2009; 122(3):216-38

[2] MF Essop: **Cardiac metabolic adaptions in response to chronic hypoxia.** *J Physiol* 2007, 584(Pt3): 715-726

[3] FJ Giordano: **Oxygen, oxidative stress, hypoxia, and heart failure.** *J Clin Invest* 2005, 115(3):500-8

[4] KR Götz *et al.*: **Transgenic mice for real-time visualization of cGMP in intact adult cardiomyocytes.** *Circ Res* 2014, 114(8): 1235-45

## A29 A computational approach to understand the mechanisms of pharmacological modulation of guanylate cyclases

### Luis Agulló, Martin Floor, Jordi Villà-Freixa

#### Computational Biochemistry and Biophysics Laboratory (CBBL), U_ScienceTech (UST), University of Vic - Central University of Catalonia (UVIC-UCC), Vic, Barcelona, Spain

##### **Correspondence:** Luis Agulló (luis.agullo@uvic.cat)

**Background:**

Guanylate cyclases are important drugs targets for several disorders, including cardiovascular, pulmonary and renal diseases. Only heme-dependent stimulators and heme-independent activators of soluble guanylate cyclases (sGCs) have been amply studied, although even in this case their mechanism of action is unclear. We have recently described the potential binding site of heme-dependent stimulators [1]. In this study, we are: first, investigating the mechanism of action of these heme-dependent stimulators of sGCs, and, second, searching for new modulators of membrane guanylate cyclases (mGCs). In both cases, we are using the recently available structural data for this class of enzymes.

**Methods:**

Basically, the general procedure includes first comparative modelling of the target protein (based on the satisfaction of spatial restraints), docking of a subset of compounds pertaining to the ZINC-database (Zbc-ZINC Biogenic compounds; 180,313 molecules) to several conformations of the potential “druggable” sites in the proteins of interest, and selection of the compounds with the best score for site specificity. Drugs with poor site and protein specificity are discarded. Molecular dynamics is used for the selection of protein conformations submitted to drug docking (after evaluation of the volume of the cavities during the whole simulation) and to analyze the time-dependent structural changes evoked by the best-scoring compounds. Ligand binding affinities are evaluated by the Linear Interaction Energy (LIE) method [2].

**Results and conclusions:**

**Fig. 4:**
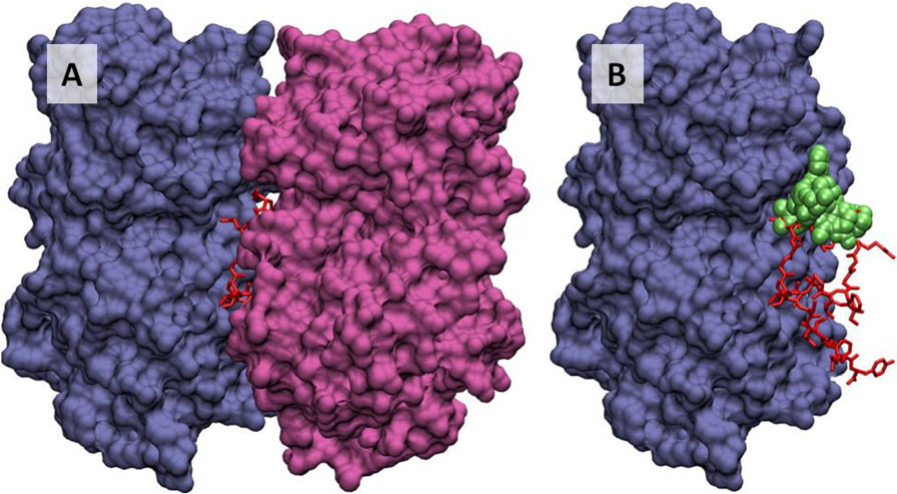
**(Abstract A29).** Binding sites of the drugs with the best score in a structure-based virtual screening (SBVS) for modulators of NPRA. **a** chains A and B of the receptor domain of human NPRA (in a surface representation; mauve and blue) with bound ANP (in a licorice representation; red); (**b**) chain B (in this view chain A has been eliminated for simplicity) with ANP and the six drugs with the best score (see Table 3) in a preliminary SBVS (in sphere representations; green).

Molecular dynamics of the catalytic domain of human sGC (both, in its inactive and active conformations) in the presence or absence of YC-1 are underway to identify its possible mechanism of action. On the other hand, different sites (including catalytic and receptor domains) of the atrial natriuretic peptide receptor 1 have been submitted to structure-based virtual screening (SBVS) in order to find new modulators of mGCs. Preliminary results of this screening are shown on Table 3 and Fig. 4 for the receptor-binding site.

**Table 3:** **(Abstract A29).** Drugs with the best score in a structure-based virtual screening (SBVS) for modulators of NPRA

Compounds	Predicted binding affinity (kcal/mol)
z00_092002	-11.8
z00_090230	-11.7
z00_032491	-11.7
z00_032002	-11.6
z01_003076	-11.5
z01_031318	-11.5

Computational structure-based approaches can be very useful in the understanding of the mechanisms of drug function and in the search of new modulators of important drug targets. The new ligands thus discovered could be of invaluable help to dissect the role of these proteins in their physiological contexts.

Conflicts of interest

*none.*

**References:**

[1]. Agulló L, Buch I, Gutiérrez-de-Terán H, Garcia-Dorado D, Villà-Freixa J. **Computational exploration of the binding mode of heme-dependent stimulators into the active catalytic domain of soluble guanylate cyclase.** *Proteins.* 2016, 84:1534-48.

[2]. Hansson T, Marelius J, Aqvist J. **Ligand binding affinity prediction by linear interaction energy methods.** *J Comput Aided Mol Des.* 1998, 12:27-35.

## A30 Development of cGMP-sensors targeted to TnI and PLB reveal difference in compartmentation of the natriuretic peptide receptors A and B

### Ornella Manfra^1,2^, Gaia Calamera^1,2^, Nicoletta C. Surdo^3^, Silja Meier^1,2^, Alexander Froese^4^, Viacheslav O. Nikolaev^4^, Manuela Zaccolo^3^, Finn Olav Levy^1,2^, Kjetil Wessel Andressen^1,2^

#### ^1^Department of Pharmacology, University of Oslo and Oslo University Hospital, Oslo, Norway; ^2^Center for Heart Failure Research, University of Oslo, Oslo, Norway; ^3^Department of Physiology, Anatomy and Genetics, Oxford University, Oxford, UK; ^4^German Center for Cardiovascular Research, University Medical Center Hamburg-Eppendorf and Institute of Experimental Cardiovascular Research, Hamburg, Germany

##### **Correspondence:** Kjetil Wessel Andressen **(**kjetilwa@medisin.uio.no**)**

**Background:**

Natriuretic peptide receptor-B (NPR-B; GC-B) stimulation by C-type natriuretic peptide (CNP) causes a lusitropic and negative inotropic response, through cGMP-mediated phospholamban (PLB) and troponin I (TnI) phosphorylation. Despite similar increases in cGMP, these effects are not mimicked by NPR-A (GC-A) stimulation by brain natriuretic peptide (BNP). Thus, the mechanisms of the differential cGMP signalling and compartmentation remain unclear.

**Objective:**

Clarify the organization of functional cGMP compartments and the role of phosphodiesterases (PDEs) in both neonatal and adult rat cardiac myocytes.

**Methods and Results:**

In ventricular strips, NPR-B-stimulation induced a lusitropic response and negative inotropic response that was enhanced by inhibition of PDE3. Fluorescence resonance energy transfer (FRET)-based sensors for cGMP subcellularly targeted to proteins that regulate inotropic responses (TnI and PLB) were constructed. Measurements of subcellular changes in single cardiac myocytes revealed that NPR-A- and NPR-B-stimulation increased cGMP near PLB, whereas only NPR-B-stimulation increased cGMP near TnI. PDE2 and PDE3 regulated cGMP in both compartments. By combining scanning ion conductance microscopy (SICM), FRET and local receptor stimulation, we demonstrate that NPR-B receptors both in the T-tubules and on the cell crests are able to increase cGMP similarly near both TnI and PLB.

**Conclusions:**

NPR-A receptors increase cGMP only near PLB and does not modify lusitropic or inotropic responses. The cGMP-mediated lusitropic and negative inotropic responses generated by NPR-B in cardiac myocytes is compartmentalized near both TnI and PLB. The developed targeted sensors are novel tools to characterize the cGMP compartments that regulate inotropic and lusitropic responses.

***Competing interest***

*The authors do not have a potentially perceived conflict of interest.*

## A31 NO-GC in pericytes as modulator of lung fibrosis

### Annemarie Aue, Fabian Schwiering, Dieter Groneberg, Andreas Friebe

#### Physiologisches Institut, Universität Würzburg, Würzburg, Germany

##### **Correspondence:** Annemarie Aue (annemarie.aue@uni-wuerzburg.de)

**Background:**

Lung fibrosis is a chronic disease with a median survival of three years. Underlying mechanisms are not fully understood and effective therapies are lacking. During fibrosis, myofibroblasts are responsible for an excessive production and deposition of extracellular matrix (ECM) proteins and collagen. As consequence, functional tissue is replaced by scar tissue. The origin of myofibroblasts remains unresolved and pericytes have been implicated as a source of myofibroblasts. NO-sensitive guanylyl cyclase (NO-GC), the receptor for NO, has been recently shown by our group to be highly expressed in retinal pericytes. Therefore, we hypothesized NO-GC expression in lung pericytes and a potential modulation of fibrotic processes by the NO/cGMP cascade.

**Methods:**

To investigate a possible participation of NO-GC in lung fibrosis we used our general KO mice (GCKO). Expression of Cre recombinase under the control of the SMMHC promotor led to deletion of NO-GC in SMC but surprisingly also in lung pericytes; therefore, we used the 'smooth muscle cell-specific' KO mice as model for pericyte-directed NO-GC deletion. Fibrosis was induced by a single dose of bleomycin (2 U/kg) via intratracheal instillation. 21 days after instillation lungs were harvested. Measurements of dry lung weight and collagen content were used to evaluate fibrotic responses. Immunohistochemistry was performed to characterize pericytes and lung fibrosis.

**Results:**

In fact, NO-GC expression can be detected in lung pericytes by colocalization with established pericyte markers PDGFRβ and desmin. Healthy lungs did not express αSMA, a typical marker of fibrosis. Bleomycin treated mice developed fibrosis shown by an increase in lung dry weight and collagen content. Both parameters were further elevated in GCKO mice and in animals deficient in pericyte-NO-GC compared to control animals. In control mice, fibrotic areas were defined by cells expressing PDGFRβ and αSMA without NO-GC expression. In GCKO, fibrotic areas were bigger and more diffuse compared to control animals.

**Conclusion:**

Absence of NO-GC leads to a deterioration of bleomycin-induced lung fibrosis in mice. Thus, NO/cGMP signalling appears to be protective in the development of lung fibrosis.

***Competing interest***

*The authors declare no conflict of interest.*

## A32 The phosphodiesterase inhibitors sildenafil and tadalafil accumulate inside platelets in the presence of cyclic guanosine monophosphate

### Gzona Bajraktari^1^, Jürgen Burhenne^1^, Walter E. Haefeli^1^ and Johanna Weiss^1^

#### ^1^Department of Clinical Pharmacology and Pharmacoepidemiology, University of Heidelberg, Im Neuenheimer Feld 410, 69120 Heidelberg, Germany

##### **Correspondence:** Gzona Bajraktari **(**gzona.bajraktari@med.uni-heidelberg.de**)**

**Introduction:**

Sildenafil and tadalafil are phosphodiesterase 5 (PDE5) inhibitors widely used as therapy in erectile dysfunction and pulmonary hypertension. Their mechanism of action is the elevation of intracellular cyclic guanosine monophosphate (cGMP) by inhibiting the break down by PDE5. cGMP regulates different processes like smooth muscle relaxation and platelet aggregation [1]. Binding of cGMP to the allosteric site of PDE5 causes a conformation change in the catalytic site of the enzyme, enhancing the binding affinity of PDE5 inhibitors [2-4]. Using isolated and/or recombinant PDE5, it has been demonstrated that cGMP can increase the affinity of this enzyme for sildenafil and tadalafil [5, 6], but none of them investigated the influence of this changed affinity on intracellular concentrations of PDE5 inhibitors.

**Objectives:**

The aim of this work was to demonstrate that the change of PDE5 affinity for PDE5 inhibitors provoked by cGMP leads to higher intracellular concentrations of these compounds. Platelets - possessing the entire pathway - were used as a cell system.

**Materials and methods:**

Isolated and washed platelets were incubated with sildenafil or tadalafil at different concentrations and for a range of time intervals with or without DEA NONO-ate as an NO donor. Intracellular sildenafil and tadalafil concentrations were quantified using ultra performance chromatography coupled to mass spectrometry methods and intracellular cGMP was measured with a commercial ELISA assay.

**Results:**

Intracellular cGMP concentrations increased when samples were treated with sildenafil with or without DEA NONO-ate (5 μM). Sildenafil was avidly taken up into platelets leading to substantially higher intracellular than extracellular concentrations with up to 4-fold higher concentrations in the DEA NONO-ate treated samples. Regarding time dependency a plateau was reached after 10 min. The results obtained for tadalafil were similar to those for sildenafil aside from some aspects. A plateau was reached only after 30 min, intracellular accumulation was considerably higher than for sildenafil, and the effect of DEA NONO-ate was less pronounced.

**Conclusion:**

Sildenafil and tadalafil both accumulate extensively inside platelets. cGMP concentrations present in the cell influence accumulation most likely by changing the affinity of PDE5 inhibitors for PDE5.

**Funding**

*Parts of the study were funded by Glaxo Smith Kline (Brantford, Middlesex, UK). Moreover, this research has been funded with support from the European Commission (Erasmus mundus Basileus IV program).*

**References:**

[1]. Schwarz UR, Walter U, Eigenthaler M: Taming platelets with cyclic nucleotides. Biochem Pharmacol 2001, 62:1153-1161.

[2]. Corbin JD, Francis SH: Conformational conversion of PDE5 by incubation with sildenafil or metal ion is accompanied by stimulation of allosteric cGMP binding. Cell Signal 2011, 23:1578-1583.

[3]. Blount MA, Beasley A, Zoraghi R, Sekhar KR, Bessay EP, Francis SH, Corbin JD: Binding of tritiated sildenafil, tadalafil, or vardenafil to the phosphodiesterase-5 catalytic site displays potency, specificity, heterogeneity, and cGMP stimulation. Mol Pharmacol 2004, 66:144-152.

[4]. Biswas KH, Visweswariah SS: Distinct allostery induced in the cyclic GMP-binding, cyclic GMP-specific phosphodiesterase (PDE5) by cyclic GMP, sildenafil, and metal ions. J Biol Chem 2011, 286:8545-8554.

[5]. Mullershausen F, Friebe A, Feil R, Thompson WJ, Hofmann F, Koesling D: Direct activation of PDE5 by cGMP: long-term effects within NO/cGMP signaling. J Cell Biol 2003, 160:719-727.

[6]. Rybalkin SD, Rybalkina IG, Shimizu-Albergine M, Tang XB, Beavo JA: PDE5 is converted to an activated state upon cGMP binding to the GAF A domain. EMBO J. 2003, 22:469-478.

## A33 Modulatory role of cGMP on colonic motility in the mouse

### Katharina Beck^1^, Barbara Voussen^1^, Alexander Vincent^2^, Sean P. Parsons^2^, Jan D. Huizinga^2^, Andreas Friebe^1^

#### ^1^Physiologisches Institut, Universität Würzburg, Würzburg, Germany; ^2^Farncombe Family Digestive Health Research Institute, McMaster University, Hamilton, Canada

##### **Correspondence:** Katharina Beck **(**katharina.beck1@uni-wuerzburg.de**)**

**Background:**

Regulation of gastrointestinal motility is complex and involves both excitatory and inhibitory neurotransmission. A very important inhibitory neurotransmitter is nitric oxide (NO). In the GI tract, NO-sensitive guanylyl cyclase (NO-GC), is the main receptor for NO, which leads to generation of cGMP. It is expressed in several cell types such as smooth muscle cells (SMC) and interstitial cells of Cajal (ICC). Cell-specific knockout mice for NO-GC revealed NO-GC as an intricate modulator of spontaneous contractions in the colon and specified the role of SMC and ICC in the regulation of murine colonic motility.

**Methods:**

This study focuses on the role of NO-GC in murine colonic motility. We used isometric force studies to record contraction pattern of colonic circular muscle rings from global NO-GC knockout (GCKO) and cell-specific knockout mice lacking NO-GC specifically in SMC or ICC. Moreover, video recordings of whole colon preparations were used to generate spatiotemporal maps, in order to evaluate the contraction pattern and propagation characteristics of the knockout mice. Outflow measurements gave detailed information about the efficiency of the propulsive contractions. Spatiotemporal maps enable the analysis of long distance contractions (LDC), the strongest propulsive motor pattern in the mouse colon.

**Results and conclusion:**

Isometric force studies of colon rings showed three different contractions. We recorded continuous small high frequency contractions, which were interrupted by bigger ‘intermediate contractions’, as well as, periodic ‘giant contractions’ which are characterized by a strong tonic contraction with superimposed ‘ripples’. Our results reveal a modulatory role of cGMP on intermediate and giant contractions via SMC and ICC.

Furthermore, whole colon preparations revealed cGMP as an important component of the neuronal circuitry that orchestrates the LDC, which is most likely the mouse equivalent of the High Amplitude Propagating Contraction of the human colon. Without cGMP, the frequency of LDC increases. Moreover, the LDC becomes less effective as shown by an altered LDC topography. This is likely a result of disturbed NO/cGMP signalling in ICC.

***Competing interest***

*The authors declare no conflict of interest.*

## A34 T-type Ca^2+^ channel blocker reveals novel target for pancreatic cancer therapy, Role of PKG - p21 signalling axis

### Fabiola Zakia Mónica ^1,2^, Edward Seto^1^, Ferid Murad^1^, Ka Bian^1^

#### ^1^Department of Biochemistry and Molecular Medicine, George Washington University, School of Medicine, Washington, DC, USA; ^2^Department of Pharmacology, Faculty of Medical Sciences, State University of Campinas (UNICAMP), Campinas, Sao Paolo, Brazil

##### **Correspondence:** Ka Bian **(**bcmkxb@gwu.edu**)**

Pancreatic cancer has the highest cancer mortality rate of 93%. Only 57% of pancreatic cancer patients live up to 6 months and only 15% of patients live over a year and a half. However, the effective therapies for pancreatic cancer are lacking. T-type calcium channels (transient opening calcium channels) are normally located within the brain, peripheral nervous, cardiovascular, and endocrine systems. Overexpression of T-channels in different tumor types has recently gained attention; however, the role for these channels in pancreatic cancer is not well explored. NNC 55-0396 is an analog of mibefradil (Ro 40-5967) with higher selectivity, enhanced potency and reduced side effects. It has been suggested that NNC 55-0396 can permeate through the plasma membrane and block T-calcium channels, however, the signaling pathways underlying its anti-cancer activity is unknown (1). Our studies with in four pancreatic cancer cell lines (Pa-TU-8988, Panc-1, YAPC, BxPC-3) showed that T-channel subtype Cav 3.1 (α1G) is expressed in all cell lines, whereas Cav 3.2 (α1H) only in Panc-1. The α1subunit is the primary subunit that forms the transmembrane pore of the channel. NNC 55-0396 treatment inhibited proliferation of all cell lines. The silencing Cav 3.1 by using siRNA CACNA1G in Pa-TU-8988 cells resulted similar proliferation inhibition. Cell cycle analysis in Pa-TU 8988 synchronized cells treated with NNC 55-0396 (5μM) revealed a T-channel blocker-induced G1 arrest. Thus, we further verified the expression of key genes involved in cell cycle regulation. The cyclin-dependent kinases 2 (cdk-2) and 6 (cdk-6) were significantly decreased by 0.56- and 0.38-fold, respectively while the cdk inhibitor p21 (CDKN1A) was markedly increased by 2.5 fold after treating with NNC 55-0396 (5μM, 24 hours). No significant difference was observed in cdk 4 gene expression. Similar results were observed with Western blots of CDK2, 6 and p21 Since the p21WAF1/Cip1 (p21) protein is an inhibitor of cyclin-dependent kinases, we also checked other pancreatic cells and observed that the T-channel blocker increased p21 expression in all cell lines. The cyclin-dependent kinase inhibitor p21 is a major effector of the tumor suppressor p53. However, T-Ca^2+^ channel blocker promoted p21 expression in both p53-positive (Panc-1, BxPC-3) and p53-deficient (Pa-TU-8988) cells (2), and inhibited proliferation of all tested cell lines. We propose that p21 can be activated independently of p53 in pancreatic cancer cells. T-type Ca^2+^ channel blocker may exerts transcriptional regulation of p21 directly or via either PKG I or HDAC pathways in pancreatic cancer cells.

**Conflict of interest**

*authors do not have a potentially perceived conflict of interest.*

**References:**

(1) Santoni G, Santoni M, Nabissi M. *Br J Pharmacol* 2012 (PMID: 22352795).

(2) Deer EL et al, Pancreas 2010 (PMID:20418756).

## A35 Identification of a novel antihypertensive that targets the oxidative activation of cGMP-dependent protein kinase

### Joseph R. Burgoyne^1^, Oleksandra Prysyazhna^1^, Daniel Richards^1^ and Philip Eaton^1^

#### ^1^King’s College London, Cardiovascular Division, The British Heart Foundation Centre of Excellence, The Rayne Institute, St Thomas’ Hospital, London, SE1 7EH, UK

##### **Correspondence:** Joseph R. Burgoyne **(**joseph.burgoyne@kcl.ac.uk**)**

Arterial hypertension is an increasingly prevalent condition that predisposes an individual to increased risk of cardiovascular disease and mortality. Despite the availability of antihypertensive drugs, many patients have high blood pressure that is resistant to current treatments. The discovery that cGMP-dependent protein kinase 1α (PKG1α) intermolecular disulfide formation contributes to oxidant-induced or endothelium-derived hyperpolarisation factor-dependent vasodilation [1,2], highlights a new mechanism that could be targeted therapeutically to lower blood pressure in patients resistant to current therapies. Such drugs may also offer a new, complementary first-line therapy against hypertension.

Here we developed a novel drug assay to screen a library of small electrophilic compounds, to find those that covalently target cysteine 42 of PKG1α to mimic or induce disulfide activation. 12 candidates identified in this screen were subsequently tested for their ability to relax mesenteric vessels isolated from wild-type mice. Those that successfully relaxed mesenteries were re-tested in vessels from wild-type and concomitantly compared to those isolated from C42S PKG1α knock in (KI) mice. This led to the identification of a hit compound, which we named **G1** that induced relaxation of wild-type mesenteric vessels (EC_50_ = 3.9 ±0.65uM), but to a lesser extent those isolated from C42S PKG1α KI mice (EC_50_ = 37 ±11.4uM). Furthermore, feeding of G1 (20 mg/kg for 4 days) lowered blood pressure in telemetered angiotensin-II hypertensive wild-type mice. This ability of G1 to lower blood pressure was absent in hypertensive C42S PKG1α KI mice, thus confirming selectivity for its mechanism of action. In summary, we have identified the first in a new class of antihypertensive drug that with further development, could be used clinically to lower blood pressure as a first line therapy or perhaps to treat patients resistant to current interventions.

**Conflict of interest**

*The authors declare that they do not have a potentially perceived conflict of interest.*

**References:**

[1] JR Burgoyne, M Madhani, F Cuello, RL Charles, JP Brennan, E Schröder, DD Browning, P Eaton: **Cysteine redox sensor in PKGIa enables oxidant-induced activation**. *Science* 2007 **317**: 1393-7

[2] O Prysyazhna, O Rudyk, P Eaton. **Single atom substitution in mouse protein kinase G eliminates oxidant sensing to cause hypertension**. *Nat Med* 2012 **18**:286-90

## A36 Development of FRET-based sensors with nanomolar affinity for cGMP using structure-based design

### Gaia Calamera^1,2^, Marianne Bjørnerem^1,2^, Andrea Hembre Ulsund^1,2^, Jeong Joo Kim^3^, Choel Kim^3^, Finn Olav Levy^1,2^, Kjetil Wessel Andressen^1,2^

#### ^*1*^*Department of Pharmacology, University of Oslo and Oslo University Hospital, Oslo, Norway;*^*2*^*Center for Heart Failure Research, University of Oslo, Oslo, Norway;*^3^*Department of Pharmacology, Baylor College of Medicine, Houston, Texas, USA*

##### **Correspondence:** Gaia Calamera **(**gaia.calamera@studmed.uio.no**)**

**Background:**

FRET-sensors (Fluorescence Resonance Energy Transfer) have been widely used to detect protein-protein interaction, conformational changes of proteins or to monitor levels of cyclic nucleotides (CNs). Particularly, several cAMP and cGMP FRET sensors have been developed and shown to be valuable tools for determining spatial and temporal intracellular signaling. Our aim is to monitor the dynamic changes of cGMP in specific sub-cellular microdomains in cardiac myocytes. Here, the concentration of cGMP is low, and there are few current cGMP-sensors that can monitor the low cGMP concentrations seen after guanylyl cyclase activation. In the current study, we wanted to construct novel FRET sensors with high affinity for cGMP that can measure temporal and spatial signaling of guanylyl cyclases.

**Methods:**

We constructed novel cGMP sensors based on cGMP binding domains from Plasmodium falciparum (PfPKG) and human (PKG I and PKG II). The binding domains were sandwiched between FRET pairs, either cyano (CFP) and yellow (Venus) or blue (T-sapphire) and red (Dimer2) fluorescent proteins. To improve affinity and selectivity (against cAMP), mutations in the cGMP-binding pocket improved affinity and also selectivity. All sensors were expressed in HEK293 cells and FRET efficacy was determined either in vitro or in intact cells stimulated with an NO donor (stimulates soluble guanylyl cyclase).

**Results:**

We designed three sensors with high affinity (~10 nM) for cGMP and high selectivity towards cAMP (up to 1.000 fold). All sensors yielded a large dynamic range (10-20 % change in FRET) and responded to NO-donor in HEK293 cells.

**Conclusions:**

The new sensors based on modified cGMP binding domains from PKG I has the ability to detect cGMP with high affinity and selectivity. We termed the sensor with highest selectivity “ScGI sensor” (Selective cGMP-dependent protein kinase I sensor) and believe it will be a valuable tool to unravel spatial and temporal cGMP-mediated intracellular signaling in cardiac myocytes.

***Competing interest***

*The authors do not have a potentially perceived conflict of interest.*

**References:**

[1] Nikolaev VO, Gambaryan S, Lohse MJ: **Fluorescent sensors for rapid monitoring of intracellular cGMP.** *Nat Methods.* 2006, **3**:23-5.

[2] Michael Russwurm, Florian Mullershausen, Andreas Friebe, Ronald Jäger, Corina Russwurm, Doris Koesling: **Design of fluorescence resonance energy transfer (FRET)-based cGMP indicators: a systematic approach.** *Biochem J.* 2007, **407**:69–77.

[3] Yusuke Niino, Kohji Hotta, Kotaro Oka: **Simultaneous Live Cell Imaging Using Dual FRET Sensors with a Single Excitation Light.** *PLoS ONE* 2009, **4**:e6036.

[4] Jeong Joo Kim, Christian Flueck, Eugen Franz, Eduardo Sanabria-Figueroa, Eloise Thompson, Robin Lorenz, Daniela Bertinetti, David A. Baker, Friedrich W. Herberg, Choel Kim: **Crystal Structures of the Carboxyl cGMP Binding Domain of the Plasmodium falciparum cGMP-dependent Protein Kinase Reveal a Novel Capping Triad Crucial for Merozoite Egress.** *PLoS Pathog.* 2015 **11**:e1004639.

[5] Kim, J. J., Lorenz, R., Arold, S. T., Reger, A. S., Sankaran, B., Casteel, D. E.,Herberg, F. W., Kim, C. : **Crystal Structure of PKG I:cGMP Complex Reveals a cGMP-Mediated Dimeric Interface that Facilitates cGMP-Induced Activation**. Structure, 2016. **24**(5): p. 710-20.

[6] Campbell, J. C., Kim, J. J., Li, K. Y., Huang, G. Y., Reger, A. S., Matsuda, S., Sankaran, B., Link, T. M., Yuasa, K., Ladbury, J. E., Casteel, D. E., Kim, C. **Structural Basis of Cyclic Nucleotide Selectivity in cGMP-dependent Protein Kinase II.** J Biol Chem, 2016. **291**(11): p. 5623-33.

## A37 Oxidant sensor in the cGMP-binding pocket of cGMP-dependent protein kinase Iα regulates nitroxyl-mediated kinase activity

### Sonia Donzelli^1,2^, Mara Goetz^1**,2**^, Kjestine Schmidt^2,3^, Markus Wolters^4^, Konstantina Stathopoulou^1,2^, Oleksandra Prysyazhna^5^, Jenna Scotcher^5^, Christian Dees^6^, Hariharan Subramanian^2,7^, Elke Butt^6^, Alisa Kamynina^5^, S. Bruce King^8^, Viacheslav O. Nikolaev^2,7^, Cor de Witt^2,3^, Lars I. Leichert^9^, Robert Feil^4^, Philip Eaton^5^, Friederike Cuello^1,2^

#### ^*1*^Department of Experimental Pharmacology and Toxicology, Cardiovascular Research Center, University, Medical Center Hamburg-Eppendorf, Martinistrasse 52, 20246 Hamburg, Germany; ^2^DZHK (German Center for Cardiovascular Research), partner site Hamburg/Kiel/Lübeck, University Medical Center Hamburg-Eppendorf, Martinistrasse 52, 20246 Hamburg, Germany; ^3^Department of Physiology, University of Lübeck, DZHK Partner Site Hamburg/Kiel/Lübeck, University Heart Centre Lübeck, Lübeck, Germany; ^4^Interfakultäres Institut für Biochemie, University of Tübingen, Hoppe-Seyler-Str. 4, 72076 Tübingen, Germany; ^5^King´s College London, Cardiovascular Division, British Heart Foundation Centre of Excellence, the Rayne Institute, St Thomas´ Hospital, London SE17EH, United Kingdom; ^6^Institute of Experimental Biomedicine II, University Medical Center Würzburg, Grombühlstraße 12, 97080 Würzburg, Germany; ^7^Institute of Experimental Cardiovascular Research, University Medical Center Hamburg-Eppendorf, Martinistrasse 52, 20246 Hamburg, Germany; ^8^Department of Chemistry, Wake Forest University, Winston-Salem, North Carolina 27109, USA; ^9^Institute of Biochemistry and Pathobiochemistry - Microbial Biochemistry, Ruhr University Bochum, Universitätsstrasse 150, 44780 Bochum, Germany

##### **Correspondence:** Friederike Cuello **(**f.cuello@uke.de**)**

**Background:**

Although nitroxyl (HNO) is produced endogenously, with pharmacological donors showing broad therapeutic promise, its mechanisms of action is not fully elucidated.

**Methods and Results:**

Mass spectrometry and site-directed mutagenesis showed that the chemically distinct HNO donors 1-nitrosocyclohexyl acetate or Angeli`s salt each induced disulfides at disparate sites within cGMP-dependent protein kinase I-alpha (PKGIα). These included interdisulfide between Cys42 of the two identical subunits of the kinase, as well as a previously unobserved intradisulfide between Cys117 and Cys195 in the high affinity cGMP-binding site. Kinase activity was monitored in cells separately transfected with wildtype (WT), Cys42Ser or Cys117/195Ser PKGIα that cannot form the inter- or intradisulfide, respectively. This revealed HNO enhanced WT kinase activity, an effect significantly attenuated in inter- or intradisulfide-deficient PKGIα. Cys42/117/195Ser PKGIα was completely resistant to HNO-induced activation. To investigate whether the intradisulfide modulates cGMP binding, real-time imaging was performed in vascular smooth muscle cells expressing a FRET-biosensor comprising the cGMP-binding sites of PKGIα. HNO induced FRET changes similar to those elicited by an increase of cGMP, suggesting that HNO-induced intradisulfide formation is associated with activation of PKGIα. Consistent with the intradisulfide lowering affinity for its classical second messenger activator, cGMP was unable to potentiate HNO-intradisulfide induced PKGIα activity. Intradisulfide formation in PKGIα correlated with enhanced HNO-mediated vasorelaxation in mesenteric arteries *in vitro* and arteriolar dilation *in vivo* in mice.

**Conclusion:**

We conclude that HNO induces an intradisulfide in the high affinity cGMP-binding site of PKGIα, inducing the same effect as cGMP binding, namely kinase activation and thus vasorelaxation.

***Competing interest***

*No conflict of interest to declare.*

## A38 Shedding light on CNP-responsive vascular smooth muscle cells

### Hyazinth Dobrowinski^#^, Moritz Lehners^#^, Michael Paolillo Hannes Schmidt, Robert Feil

#### Interfakultäres Institut für Biochemie, University of Tübingen, Tübingen, Germany

##### **Correspondence:** Hyazinth Dobrowinski (hyazinth.dobrowinski@ifib.uni-tuebingen.de)

^#^equal contribution

Cyclic GMP regulates multiple functions in the cardiovascular system. It is generally accepted that vascular smooth muscle cells (VSMCs) can generate cGMP through NO-activated soluble guanylate cyclase as well as transmembrane guanylate cyclases GC-A and GC-B that are stimulated by atrial natriuretic peptide (ANP) and C-type natriuretic peptide (CNP), respectively. Studies with atherosclerotic mice in which either NO-activated guanylate cyclase or cGMP-dependent protein kinase I had been deleted suggest that cGMP signaling regulates dynamic changes of VSMC growth and phenotype (contractile vs. synthetic) during vascular remodeling. However, the distribution and function of NO-, ANP-, and CNP-responsive cells in a given population of VSMCs is not clear. Here, we used transgenic cGMP sensor mice that express the fluorescence resonance energy transfer-based cGMP sensor cGi500 to characterize cGMP responses in live VSMC populations at the singe-cell level. In primary VSMCs isolated from ubiquitously expressing cGi500 mice, we observed a strong heterogeneity of cGMP signals triggered by ANP, CNP, or the NO-releasing compound DEA/NO. Interestingly, differential cGMP responses were associated with specific VSMC phenotypes. Contractile VSMCs (defined by strong expression of αSMA and SM22α) responded stronger to ANP than to CNP, while synthetic VSMCs (defined by weak expression of αSMA and SM22α) responded stronger to CNP than to ANP. Passaging or growth on fibronectin, maneuvers known to promote the synthetic VSMC phenotype, increased the fraction of CNP-responsive cells. To confirm that the CNP-preferring cells originate from smooth muscle cells, we isolated VSMCs from transgenic mice that expressed the cGi500 sensor specifically in smooth muscle cells [SM22Cre x R26-CAG-mT/cGi500(L2)]. These cultures also contained a heterogeneous population of ANP- and CNP-preferring cells demonstrating that different VSMC phenotypes vary in their combination of cGMP signaling components. In line with this concept, experiments with VSMCs expressing β-galactosidase under the control of the GC-B promoter indicated that expression of the CNP receptor parallels the development of the synthetic VSMC phenotype during cell culture. To test the relevance of our *in vitro* findings for the *in vivo* situation, we measured cGMP signals in aortae of healthy cGMP sensor mice. These aortae showed cGMP responses to ANP and NO, but not to CNP, supporting the hypothesis that CNP-responding synthetic VSMCs develop *in vivo* only under pathologic conditions such as atherosclerosis. In the future, we will employ biosensor technology to further investigate the link between CNP-induced cGMP signaling, VSMC plasticity and vascular disease under close-to-native conditions to gain novel insights into human cardiovascular disease states.

**Funding**

*This work was supported by DFG grant FOR 2060.*

***Competing interest***

*The authors declare no competing financial interests.*

## A39 A new look at cGMP signaling, shear stress, and thrombosis

### Susanne Feil^1#^, Lai Wen^1#^, Markus Wolters^1^, Martin Thunemann^1^, Kjestine Schmidt^2^, Marcus Olbrich^3^, Harald Langer^3^, Meinrad Gawaz^3^, Andreas Friebe^4^, Cor de Wit^2^, Robert Feil^1^

#### ^1^Interfakultäres Institut für Biochemie, Universität Tübingen, Tübingen, Germany; ^2^Institut für Physiologie, Universität zu Lübeck, Lübeck, Germany; ^3^Department of Cardiology & Cardiovascular Medicine, University of Tübingen, Tübingen, Germany; ^4^Physiologisches Institut, Universität Würzburg, Würzburg, Germany

##### **Correspondence:** Susanne Feil (susanne.feil@uni-tuebingen.de)

^#^equal contribution

It is well known that NO supplied by the endothelium activates NO-sensitive guanylate cyclase (NO-GC) in platelets, resulting in an increase in intraplatelet cGMP. However, the spatiotemporal dynamics of cGMP signals in platelets and their functional relevance during hemostasis and thrombosis are largely unknown. Here, we used FRET-based cGMP sensor mice for visualization of platelet cGMP signals in real time during thrombus formation *ex vivo* and *in vivo*. The cGMP concentration was then correlated with functional parameters such as platelet aggregation and thrombus growth. As expected, NO triggered strong cGMP elevations in platelet thrombi formed under flow *in vitro*. Surprisingly, we found that NO-induced cGMP signals in platelets were dramatically increased by fluid flow/shear stress. Simultaneous measurements of cGMP and Ca^2+^ revealed that the concentrations of these two second messengers had an inverse relationship. In the presence of NO, application of flow increased cGMP and suppressed Ca^2+^ signals. Furthermore, cGMP inhibited platelet adhesion to fibrinogen and fibronectin *in vitro*. To test the *in vivo* relevance of our findings, we induced thrombosis in platelet-specific cGMP sensor mice by mechanical or laser-induced injury of cremaster arterioles and monitored cGMP during thrombus formation by intravital FRET imaging. These experiments showed that cGMP was indeed elevated in platelets during thrombus growth *in vivo*. Experiments with platelet-specific NO-GC knockout mice validated the FRET/cGMP measurements and showed that flow-mediated cGMP signaling limits thrombosis. Taken together, this study has discovered NO/cGMP signaling as a new mode of platelet mechanotransduction. We propose a revised model for the role of cGMP signaling in thrombosis. In this model an increase in shear stress during thrombus formation acts as an auto-regulatory brake to prevent thrombus overgrowth and vessel occlusion via an increase in cGMP followed by a decrease in the intraplatelet Ca^2+^ concentration.

**Funding**

*This work was supported by DFG grant KFO 274.*

***Competing interesting***

*The authors declare no competing financial interests.*

## A40 Mechanisms associated with cGMP-dependent activation of *Plasmodium falciparum* PKG

### Eugen Franz^1^, Jeong Joo Kim^1,2^, Daniela Bertinetti^1^, Choel Kim^2^, Friedrich W. Herberg^1^

#### ^1^ Department of Biochemistry, University of Kassel, 34132 Kassel, Germany; ^2^ Department of Pharmacology, Baylor College of Medicine, One Baylor Plaza, Houston, Texas, USA

##### **Correspondence:** Eugen Franz (franz.eugen@uni-kassel.de)

**Background:**

Protozoan parasites of the subphylum Apicomplexa are significant threats to human and animal health. Malaria is still one of the most threatening infectious diseases worldwide [5,8]. In particular, treatment of malaria has been hampered by drug resistance. Therefore, the development of innovative therapies with novel drug targets is needed. The most dangerous and deadliest variant of malaria is caused by protozoans of the *Plasmodium falciparum*, which are transmitted by the female *Anopheles* mosquitoes.

Previous studies revealed that *P. falciparum* cGMP-dependent protein kinase (PfPKG) has three functional cGMP (CNB-A/B/D) and one degenerate (CNB-C) binding site [2,3]. PfPKG is crucial for both sexual and asexual proliferation in the mosquito and the human host [1,5,7,8]. Overall sequence and domain organisation of PfPKG significantly differ from mammalian PKG representing a strong drug target for malaria. However, the regulation mechanisms of PfPKG are largely unknown. Our previous studies demonstrated that the C-terminal CNB-D is most important for the activation of PfPKG [3,4]. Most of protein kinases have an N-terminal amphipathic helix (A-helix) shielding a hydrophobic surface of the catalytic domain. Although the A-helix has a highly conserved structural motif of serine/threonine kinases, its functional role in activation and regulation is not known. The crystal structure of PfPKG reveals that the αC-helix of the CNB-D corresponds to this structural motif. Therefore, we focused in particular on different CNBs and the catalytic domain to investigate the activation mechanism of PfPKG.

**Results:**

To investigate the activation mechanism of PfPKG, we studied cGMP binding and activation of various deletion constructs using fluorescence polarization (FP) and a microfluidic mobility-shift assay (MSA).

Our results showed differences in the activation mechanism of PfPKG compared to mammalian PKG. Strikingly, our measurements showed that a deletion construct (residues 401-853) only containing CNB-D and catalytic domain remains inactive without cGMP and becomes active in the presence of cGMP despite the missing an N-terminal autoinhibitory sequence (AS). Furthermore, several deletion constructs of the PfPKG were generated, in which the individual CNBs were sequentially deleted: PfPKG 32-853 (without AS), PfPKG 158-853 (without AS and CNB-A), PfPKG 275-853 (without AS and CNB-A/B) and PfPKG 401-853 (CNB-D with kinase domain). Analogously to PfPKG 401-853, all deletion constructs revealed a low basal activity and can be activated 20-25-fold by cGMP suggesting that inhibition of activity may not depend on the interaction between the AS and the catalytic domain. While these deletion constructs showed similar activation constants (K_A_ about 300 nM) compared to the full length PfPKG, the Hill’s coefficient changed from 1.7 (PfPKG 32-853) to 0.7 (PfPKG 401-853) indicating that CNB-A and CNB-B are required for positive cooperativity.

Structural comparison of PfPKG in inactive and active conformations suggested that three amino acids are crucial in stabilizing different conformation of the αC-helix. The inactive conformation showed that an arginine (R528) of the αC-helix forms a hydrogen bond with an aspartate (D597) in the catalytic domain. The cGMP binding to the CNB-D displaces R528 towards the cGMP binding pocket and R528 interacts with a tyrosine residue (Y480) instead forming a stable salt bridge. Our data show that mutating these residues in the deletion mutant (PfPKG 401-853) increases activation constant without changing its affinity for cGMP. In comparison to wild type (PfPKG 401-853), single mutant constructs (Y480F, R528K and D597N) have similar affinities (EC_50_ about 40 nM) for cGMP. However, Y480F mutation showed a 5-fold increased K_A_-value (about 1500 nM) and R528K as well as D597N a 6-fold decreased K_A_-value (about 70 nM) compared to wild type. In contrast to wild type (PfPKG 401-853), R528K has a great impact on the specific activity (about 0.3 vs. 5-6 U/mg).

**Conclusion:**

Our data highlight the critical role of the CNB-D domain in activation and regulation of PfPKG. Furthermore, CNB-D is essential for the function and the stability of the catalytic domain. cGMP binding to the CNB-D domain causes a conformational change in particular in the αC-helix, which keeps the catalytic domain to an active conformation. A unique salt bridge between R528 of the αC-helix and Y480 of the CNB-D domain stabilizes PfPKG in an active conformation. Our results clearly demonstrate differences in the activation mechanisms between PfPKG and human PKG suggesting that PfPKG can be differentially targeted.

**References:**

[1] Alam M, *Phosphoproteomics reveals malaria parasite Protein Kinase G as a signaling hub regulating egress and invasion*, Nature Communications 2015, 6: 7285.

[2] Baker DA, *Cyclic nucleotide signaling in malaria parasites*, Cellular Microbiology 2011, 13: 331-339.

[3] Deng W, *The role of two novel regulatory sites in the activation of the cGMP-dependent protein kinase from Plasmodium falciparum*, Biochem J. 2003, 374: 559-565.

[4] Garcia L, *Malaria*, Clinics in laboratory medicine 2010, 30: 93-129.

[5] Govindasamy K, *Invasion of hepatocytes by Plasmodium sporozoites requires cGMP-dependent protein kinase and calcium-dependent protein kinase 4*, Mol Microbiol. 2016, 102(2): 349-363.

[6] Kim J, *Crystal structures of the carboxyl cGMP binding domain of the Plasmodium falciparum cGMP-dependent protein kinase reveal a novel capping triad crucial for merozoite egress*, PLoS Pathog. 2015, 11(2): e1004639.

[7] McRobert L, *Gametogenesis in Malaria Parasites Is Mediated by the cGMP- Dependent Protein Kinase*, PLoS Biol. 2008, 6: e139.

[8] Taylor H, *The Malaria Parasite Cyclic GMP-Dependent Protein Kinase Plays a Central Role in Blood-Stage Schizogony*, Eukaryotic Cell 2010, 9: 37-45.

## A41 Riociguat for the treatment of pulmonary arterial hypertension (PAH): 2-year results from the PATENT-2 long-term extension

### Hossein-Ardeschir Ghofrani^1^, Friedrich Grimminger^1^, Ekkehard Grünig^2^, Yigao Huang^3^, Pavel Jansa^4^, Zhi Cheng Jing^5^, David Kilpatrick^6^, David Langleben^7^, Stephan Rosenkranz^8^, Flavia Menezes^9^, Arno Fritsch^10^, Sylvia Nikkho^11^, Reiner Frey^11^, Marc Humbert^12^

#### ^1^Department of Internal Medicine, University of Giessen and Marburg Lung Center (UGMLC), Giessen, Germany; ^2^Centre for Pulmonary Hypertension at the Thoraxclinic of University Hospital Heidelberg, Heidelberg, Germany; ^3^Department of Cardiology, Guangdong General Hospital and Guangdong Cardiovascular Institute, Guangzhou, Guangdong, China; ^4^Department of Cardiology and Angiology of the First Faculty of Medicine and General Teaching Hospital, Prague, Czech Republic; ^5^State Key Laboratory of Cardiovascular Disease FuWai Hospital, National Center for Cardiovascular Disease, Peking Union Medical College and Chinese Academy of Medical Sciences, Beijing, China; ^6^Discipline of Medicine, University of Tasmania, Hobart, Australia; ^7^Center for Pulmonary Vascular Disease, McGill University, Montreal, QC, Canada; ^8^Department III of Internal Medicine and Cologne Cardiovascular Research Centre, Cologne University Heart Centre, Cologne, Germany; ^9^Bayer HealthCare Pharmaceuticals, São Paulo, Brazil; ^10^Global Clinical Development, Bayer AG, Wuppertal, Germany; ^11^Global Clinical Development, Bayer AG, Berlin, Germany; ^12^Université Paris-Sud, Université Paris-Saclay, Le Kremlin Bicêtre, France

##### **Correspondence:** Hossein-Ardeschir Ghofrani **(**ardeschir.ghofrani@innere.med.uni-giessen.de**)**

**Background:**

PAH is a chronic condition with a poor prognosis characterized by increased pulmonary vascular resistance. Riociguat, a soluble guanylate cyclase stimulator, is the first member of this novel class of PAH therapies. In the 12-week PATENT-1 study, riociguat significantly improved 6-minute walking distance (6MWD), and other secondary endpoints in patients with PAH. We present analyses from the long-term PATENT-2 extension study and analyses of correlation between efficacy endpoints and long-term outcomes.

**Methods:**

PAH patients who were treatment-naïve or pretreated with endothelin receptor antagonists or prostanoids entered PATENT-2 after completing PATENT-1 without ongoing riociguat-related serious adverse events (SAEs). All patients received riociguat individually adjusted up to 2.5 mg three times daily. Primary endpoints were safety and tolerability; secondary endpoints included 6MWD, World Health Organization functional class (WHO FC), *N*-terminal prohormone of brain natriuretic peptide (NT-proBNP), survival, and clinical worsening-free survival. Correlation between efficacy parameters and long-term outcomes was assessed using Kaplan–Meier analyses and a Cox proportional-hazards regression model.

**Results:**

Of 405 patients completing PATENT-1, 396 (98%) entered PATENT-2. At this cut-off (March 2014), 275 (69%) patients were ongoing, 307 (78%) had received ≥2 years of treatment, and 13 (3%) had switched to the commercial drug. At 2 years of PATENT-2, 258/307 (84%) patients were receiving the maximum riociguat dose of 2.5 mg tid, 31/307 (10%) were receiving 2 mg tid, 12/307 (4%) were receiving 1.5 mg tid, 3/307 (1%) were receiving 1 mg tid, and 3/307 (1%) were receiving 0.5 mg tid. Riociguat was well tolerated; 11% of patients withdrew due to adverse events (AEs). There were 13 (3%) drug-related SAEs of syncope and 4 (1%) drug-related SAEs of pulmonary bleeding. All SAEs reported were within the range of the known safety profile for riociguat. At 2 years, mean±SD 6MWD increased from PATENT-1 baseline by +47±85 m (n=296) and WHO FC improved/stabilized/worsened in 33/58/9% of patients (n=306). At 2 years, survival was 93% and 17% of former therapy-naïve patients were receiving additional PAH therapy. Measurements of 6MWD, WHO FC, and NT-proBNP at baseline and after 12 weeks of treatment with riociguat correlated significantly with long-term survival and clinical worsening-free survival.

**Conclusions:**

Riociguat has a good long-term safety profile and shows sustained clinical effect for up to 2 years in PAH patients. The correlation of 6MWD, WHO FC, and NT-proBNP with long-term survival and clinical worsening-free survival emphasizes the prognostic value of 6MWD, WHO FC, and NT-proBNP for PAH patients.

**Conflict of interest:**

*Hossein Ardeschir Ghofrani has received personal fees for board membership from Actelion, Bayer AG, GSK, Novartis, Pfizer, Bellerophon Therapeutics, personal fees for consultancy from Actelion, Bayer AG, GSK, Novartis, Pfizer, Belleraphon Therapeutics and United Therapeutics, personal fees for paid lectures from Actelion, Bayer AG, GSK, Novartis, Pfizer, United Therapeutics, grants fees from Actelion, Bayer AG, Pfizer and Novartis and personal fees for travel/accommodation expenses from Actelion, Bayer AG, GSK, Novartis, Pfizer, United Therapeutics.*

*Fredrich Grimminger has received grant fees from Bayer Healthcare and personal fees from Actelion, Bayer Healthcare, Lilly, Novartis and Pfizer.*

*Ekkehard Grünig has received grant fees from Bayer AG, Actelion, GSK, Lilly, Pfizer, personal fees from Bayer AG, Miltenyi, Novartis and United Therapeutics and non-financial support from Alexion and Novartis.*

*Yigao Huang has received personal fees from Bayer AG (board/advisory committee member).*

*Pavel Jansa has received investigator fees from Actelion and Bayer AG and personal fees from Bayer AG and AOP.*

*Zhi Cheng Jing has received personal fees from Actelion, Bayer Healthcare, Pfizer and United Therapeutics.*

*David Kilpatrick has nothing to declare.*

*David Langleben has received personal fees and non-financial support from Bayer Healthcare Pharmaceuticals, Actelion, Gilead, GSK and Ikaria.*

*Stephan Rosenkranz has received grant fees from Actelion, Bayer AG, Novartis, Pfizer and United Therapeutics and personal fees from Actelion, Bayer AG, Gilead, GSK, Novartis, Pfizer and United Therapeutics.*

*Flavia Menezes is an employee of Bayer S.A.*

*Arno Fritsch is an employee of Bayer AG.*

*Sylvia Nikkho is an employee of Bayer AG.*

*Reiner Frey is an employee of Bayer AG.*

*Marc Humbert has relationships with drug companies including Actelion, Bayer, GSK, Novartis and Pfizer. In addition to being investigator in trials involving these companies, relationships include consultancy service and membership of scientific advisory boards.*

## A42 Localization of the NO-sensitive guanylyl cyclase in mouse lung

### Dieter Groneberg, Annemarie Aue, Fabian Schwiering, Andreas Friebe

#### Physiologisches Institut, Universität Würzburg, Würzburg, Germany

##### **Correspondence:** Dieter Groneberg **(**dieter.groneberg@uni-wuerzburg.de**)**

**Background:**

The NO/cGMP cascade is essential for the regulation of many physiological functions in the pulmonary system. NO-sensitive guanylyl cyclase (NO-GC) has traditionally been purified from lung due to its very high expression level compared to other organs. Up to date the exact identity of cell types expressing NO-GC in the lung are unclear.

**Methods:**

We used immunohistochemistry to localize NO-GC in perfusion-fixed mouse lung using a home-made antibody against the β_1_ subunit of NO-GC. Global NO-GC knockout (GCKO) and cell-specific models were used to prove the specificity of the immuno-signals. Several markers specific for lung cells, e.g. smooth muscle cells, fibroblasts, pericytes were used. In addition, we used Cre recombinase-mediated, cell-specific expression of the reporter dye tdTomato.

**Results and Conclusion:**

NO-GC is expressed in bronchial and vascular smooth muscle cells indicated by co-localization with the smooth muscle cell marker αSMA. In addition, NO-GC is also strongly expressed in pericytes as determined by co-staining with platelet-derived growth factor receptor ß (PDGFRß). Pericytic expression of NO-GC was corroborated by the use of another pericyte marker, desmin. These findings were confirmed with animals expressing the fluorescent dye tdTomato. NO-GC is co-localized with tdTomato expressed under the control of PDGFRß- and NG2-promotor, both markers for pericytes. Surprisingly, a subgroup of these NO-GC-positive pericytes also express smooth muscle myosin heavy chain (SMMHC) thought to be specific for smooth muscle cells. In conclusion, NO-GC is expressed in SMC and pericytes of the lung.

***Competing interest***

*The authors declare no conflict of interest.*

## A43 The role of cGMP in a cell culture model of Diabetic Nephropathy

### Manuela Harloff^1^, Joerg Reinders^2^, Jens Schlossmann^1^

#### ^1^Department of Pharmacology and Toxicology, University of Regensburg, 93053 Regensburg, Germany; ^2^Department Functional Genomics, University of Regensburg, 93053 Regensburg, Germany

##### **Correspondence:** Manuela Harloff **(**manuela.harloff@ur.de**)**

Diabetes mellitus is the world leading cause of functional kidney impairment. In adults with diabetes the risk of end-stage renal disease (ESRD) is up to 10 times higher than in those without. 30-40% of patients develop a diabetic nephropathy (DN) as a long-term complication of diabetes, which might require dialysis and renal replacement therapy.[1,2]

A specific therapy doesn’t exist so far, because the underlying mechanisms are still not completely understood.

However, many intracellular processes are already published, which might be involved in the pathogenic mechanisms of diabetic nephropathy, e. g. generation of reactive oxygen species (ROS) or advanced glycation end products (AGEs), activation of PKC or the JAK/STAT pathway.[3]

In this work, we want to find out the role of the cGMP-dependent protein kinase I (cGKI) in the development of diabetic nephropathy and if the NO/sGC/cGKI pathway could be a potential therapeutic target.

Therefore, we established a cell culture model with primary murine mesangial cells, which were incubated under normal glucose (8 mM D-Glucose) and high glucose (25 mM D-Glucose) conditions to simulate the blood glucose levels in diabetes. The cells were further treated with 1 mM 8-Br-cGMP to activate the cGKI signalling.

In our model, we confirm previously published effects of high glucose concentrations on Thrombospondin-1 (TSP-1) expression, an important regulator of transforming growth factor (TGF)-β.[4] TSP-1 expression is upregulated under high glucose conditions, but additional incubation with 8-Br-cGMP retains the protein expression equally to normal glucose conditions. Moreover, we show the same effect on Smad-2 expression, a protein which acts as a transcription factor and regulates the expression of extracellular matrix proteins like fibronectin. This could be a first hint that the activation of cGKI might have compensative effects under high glucose conditions.

To gain a more detailed insight into the cellular processes under high glucose conditions, we are using a proteomic approach with LC/MS. With this tool, we are able to identify and quantify over 2000 proteins in one cell lysate sample and to compare the proteome from differently treated cells.

***Competing interest***

*All the authors declared no competing interests.*

**Funding**

*The work was supported by the Bavarian State, Sonderforschungsbereich SFB699.*

**References:**

[1] World Health Organisation: Global Report on Diabetes. Geneva; 2016.

[2] Gallagher H, Suckling RJ: Diabetic nephropathy: **where are we on the journey from pathophysiology to treatment?** Diabetes, obesity & metabolism 2016, 18:641-647.

[3] Kanwar YS, Sun L, Xie P, Liu F-Y, Chen S: **A glimpse of various pathogenetic mechanisms of diabetic nephropathy.** Annual review of pathology 2011, 6:395-423.

[4] Wang S, Wu X, Lincoln TM, Murphy-Ullrich JE: **Expression of Constitutively Active cGMP-Dependent Protein Kinase Prevents Glucose Stimulation of Thrombospondin 1 Expression and TGF- Activity.** Diabetes 2003, 52:2144-2150.

## A44 Discovery of a conserved stimulator binding pocket in soluble guanylate cyclase

### Joon Jung^**1**^, Jessica A. Wales^**2**^, Cheng-Yu Chen^**2**^, Linda Breci^**2**^, Andrzej Weichsel^**2**^, Sylvie G. Bernier^**1**^, Robert Solinga^**1**^, James E. Sheppeck II^**1**^, Paul A. Renhowe^**1**^, and William R. Montfort^**2**^

#### ^**1**^Ironwood Pharmaceuticals, Cambridge, MA, 02142, USA; ^**2**^Department of Chemistry and Biochemistry, University of Arizona, Tucson, AZ, 8572, USA

##### **Correspondence:** Joon Jung **(**jjung@ironwoodpharma.com**)**; William R. Montfort

**Background:**

Soluble guanylate cyclase (sGC), the nitric oxide (NO) receptor, has been a target for treating cardiovascular disease for over 150 years. sGC stimulators synergize with NO and show clinical promise, but the molecular binding site and the mechanism of action have not been fully characterized. In the present study, a photoactivatable sGC stimulator was coupled with LC-MS/MS and two-dimensional NMR spectroscopy approaches were used to discover a conserved sGC stimulator binding pocket.

**Materials and Methods:**

sGC stimulator IWP-854 containing a photolyzable diazirine and PEG-linked biotin affinity tag was synthesized. Photoaffinity cross-linking studies were conducted in human and *Manduca sexta (Ms)* sGC, as well as in bacterial H-NOX homologs from *Clostridium botulinum (Cb)* and *Shewanella woodyi (Sw).* Cross-linked proteins were digested using trypsin and analysed by nano-LC-MS/MS. Additionally, protein transferred nuclear Overhauser effect spectroscopy (Tr-NOESY) and chemical perturbation in heteronuclear single quantum coherence (HSQC) NMR approaches were applied to various constructs of *Ms* sGC and bacterial H-NOX homologs.

**Results:**

IWP-854 cross-linked to the β1 heme domain of human and *Ms* sGC as well as to bacterial H-NOX homologs from *Cb* and *Sw.* Competition of IWP-854 with BAY 41-2272 and IWP-051 (the parent chemical core of IWP-854) in 1-, 5- and 50-fold molar excess decreased the cross-linked protein in a concentration dependent manner, suggesting that they bind to the same site. Cross-linked peptides were identified using mass spectrometry and a novel signature peak at 270.127 m/z and were exclusively localized to the β1 heme domain and coiled-coil domain of purified human sGC. The observation of NOE peaks derived from binding of IWP-051 to *Ms* sGC and bacterial H-NOX homologs using Tr-NOESY NMR recapitulated the cross-linking data. Additionally, several residues displayed prominent concentration-dependent shifts in resonance upon titration with IWP-051 using well-characterized HSQC spectra of *Sw* H-NOX. A computational model of stimulator binding to the sGC heme domain was generated based on current experimental data, available x-ray structures of domains and previous chemical cross-linking studies^1^.

**Conclusion:**

These data suggest stimulators bind to a cleft between two sub-domains in the sGC β1 heme domain, near a previously identified tunnel of possible importance for NO escape from the heme pocket^2^. Identifying the sGC stimulator binding pocket resolves a long-standing question and may provide a path forward for structure-guided drug discovery.

***Competing interest***

*Ironwood authors are employees of and own stock or stock options of Ironwood Pharmaceuticals. Ironwood Pharmaceuticals funded the research.*

**References:**

[1] Fritz, B.G. et al. **Molecular Model of a Soluble Guanylyl Cyclase Fragment Determined by Small-Angle X-ray Scattering and Chemical Cross-Linking.** *Biochemistry* **52**, 1568-1582 (2013).

[2] Winter, M.B., Herzik, M.A., Jr., Kuriyan, J. & Marletta, M.A. **Tunnels modulate ligand flux in a heme nitric oxide/oxygen binding (H-NOX) domain.** *Proc. Natl. Acad. Sci. USA* **108**, E881-E889 (2011).

## A45 Structure of cGMP-Dependent Protein Kinase Iα bound with a balanol like inhibitor, N46, explains its high selectivity over cAMP-Dependent Protein Kinase

### Liying Qin^1^, Ying-Ju Sung^2^, Darren Casteel^3^, and Choel Kim^1,4^

#### ^1^Verna and Marrs McLean Department of Biochemistry and Molecular Biology, Baylor College of Medicine, Houston, Texas, USA; ^2^Geisinger Commonwealth School of Medicine, Commonwealth Medical College, Scranton, PA18509, USA; ^3^Department of Medicine, University of California, San Diego, La Jolla, California, USA; ^4^Department of Pharmacology, Baylor College of Medicine, Houston, Texas, USA

##### **Correspondence:** Liying Qin **(**Liying.Qin@bcm.edu**)**

**Background:**

**Fig. 5:**
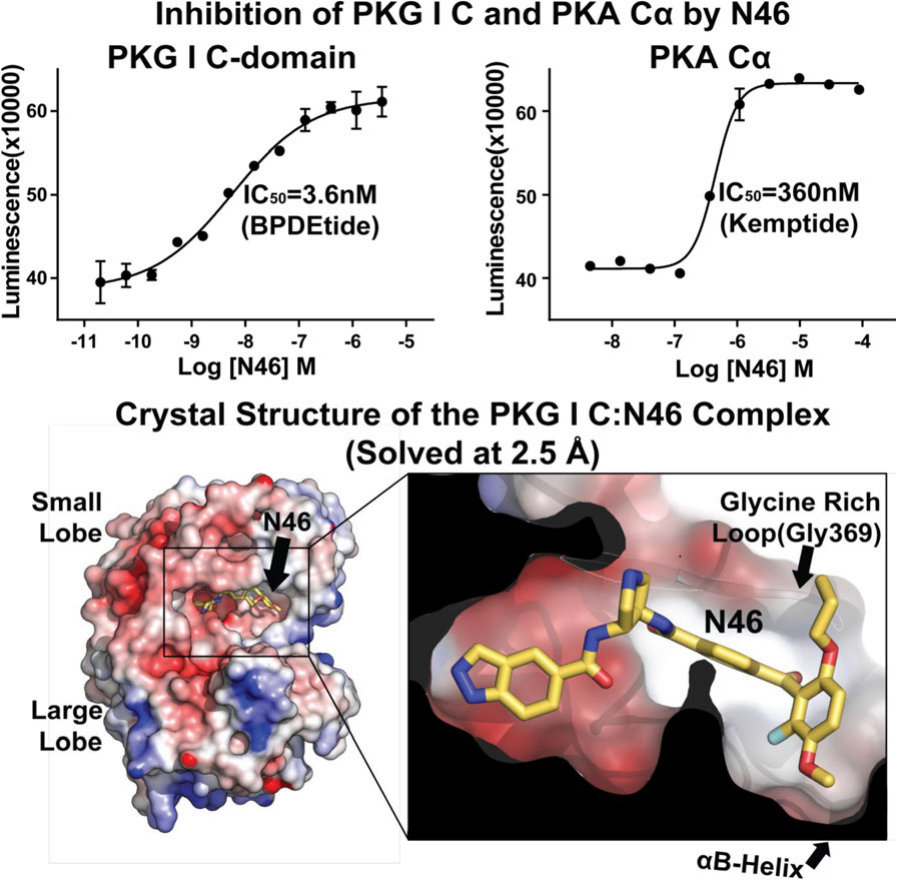
**(Abstract A45).** The crystal structure of the PKG I C:N46 complex explains high selectivity of N46 for PKG Iα. ADP to ATP conversion curves in the presence of N46 are shown for both PKG I C and PKA Cα on the top and the crystal structure of the PKG I C domain bound with N46 on the bottom. The structure of the PKG I C:N46 complex is shown in a surface representation. The zoomed-in view highlights N46 bound within the extended pocket formed between the small and large lobes. The surface is colored according to the contact electrostatic potential calculated with APBS [9]. Positively charged areas are shown in blue and negatively charged areas are in red. Each data points were measured in duplicate using Kinase-Glo Luminescent Assay. Error bars denote SEM

PKG Iα is a central regulator of smooth muscle tone and nociception and has been targeted for treating arterial and pulmonary hypertension and chronic pain[1-4]. In particular, activating PKG Iα in nociceptive neurons induces a long-term hyperexcitability that causes chronic pain in diseases such as nerve inflammation, ischemia, and metastatic bone cancer[5-7]. A recent study showed that a balanol like compound, N46, inhibits PKG Iα with high potency and selectivity (Fig. 5) and attenuates thermal hyperalgelia and osteoarthritic pain in rats[8]. However, little is known about the molecular details of PKG Iα and N46 interaction.

**Results:**

To understand the molecular basis of high potency and selectivity of N46, we measured its inhibition constants for PKG Iα catalytic (C) domain and determined their co-crystal structure at 2.5 Å resolution. Our measurements using Kinase-Glo Luminescent Assay show that N46 inhibits PKG Iα C with an IC_50_ value of 3.7 nM (using BPDEtide as a substrate) while it inhibits PKA Cα with 360 nM (using Kemptide).

The crystal structure of PKG Iα C:N46 complex reveals that N46 binds to an elongated pocket that extends from the inner edge to the outer rim of the active site of the active site within the C-domain. In particular, N46 interacts with Gly348 at the glycine rich loop and bulky residues at the inner surface of the αB helix mainly through nonpolar interactions. Our structural model of the cAMP dependent protein kinase (PKA) Cα docked with N46 shows that a serine residue (Ser53) replaces Gly348 in the glycine rich loop and may cause steric hindrance thus reducing interaction. The model also shows that the same region at the αB helix in PKA Cα has less bulky residues making no contact with N46.

**Conclusion:**

Our activation data show that N46 inhibits PKG Iα C with high affinity and selectivity over PKA C**α.** Our co-crystal structure of the PKG Iα C and N46 explains high potency and selectivity of N46 for PKG Iα and provides a starting point for structure guided design of PKG I selective inhibitors.

***Competing interest***

*The authors declare no conflict of interest.*

**References:**

[1]. Francis SH, Busch JL, Corbin JD, Sibley D: **cGMP-dependent protein kinases and cGMP phosphodiesterases in nitric oxide and cGMP action.** *Pharmacol Rev* 2010, **62:**525-563.

[2]. Hofmann F, Bernhard D, Lukowski R, Weinmeister P: **cGMP Regulated Protein Kinases (cGK).** In *cGMP: Generators, Effectors and Therapeutic Implications.* Edited by Schmidt HHHW, Hofmann F, Stasch J-P. Berlin, Heidelberg: Springer Berlin Heidelberg; 2009: 137-162.

[3]. Schlossmann J, Hofmann F: **cGMP-dependent protein kinases in drug discovery.** *Drug Discovery Today* 2005, **10:**627-634.

[4]. Schlossmann J, Schinner E: **cGMP becomes a drug target.** *Naunyn-Schmiedeberg's Archives of Pharmacology* 2012, **385:**243-252.

[5]. Gangadharan V, Wang X, Luo C: **[EXPRESS] Cyclic GMP-dependent protein kinase-I localized in nociceptors modulates nociceptive cortical neuronal activity and pain hypersensitivity.** *Mol Pain* 2017, **13:**1744806917701743.

[6]. Luo C, Gangadharan V, Bali KK, Xie RG, Agarwal N, Kurejova M, Tappe-Theodor A, Tegeder I, Feil S, Lewin G, et al: **Presynaptically localized cyclic GMP-dependent protein kinase 1 is a key determinant of spinal synaptic potentiation and pain hypersensitivity.** *PLoS Biol* 2012, **10:**e1001283.

[7]. Sung YJ, Chiu DT, Ambron RT: **Activation and retrograde transport of protein kinase G in rat nociceptive neurons after nerve injury and inflammation.** *Neuroscience* 2006, **141:**697-709.

[8]. Sung YJ, Sofoluke N, Nkamany M, Deng S, Xie Y, Greenwood J, Farid R, Landry DW, Ambron RT: **A novel inhibitor of active protein kinase G attenuates chronic inflammatory and osteoarthritic pain.** *Pain* 2017, **158:**822-832.

[9]. Baker NA, Sept D, Joseph S, Holst MJ, McCammon JA: **Electrostatics of nanosystems: application to microtubules and the ribosome.** *Proc Natl Acad Sci U S A* 2001, **98:**10037-10041.

## A46 Contradictory effects of cytosolic preparations from coronary arteries on NO-stimulated cGMP formation *in vitro*

### Alexander Kollau^1^, Andrea Neubauer^1^, Astrid Schrammel^1^, Michael Russwurm^2^, Doris Koesling^2^, Bernd Mayer^1^

#### ^1^Department of Pharmacology and Toxicology, University of Graz, A 8010 Graz, Austria; ^2^Department of Pharmacology and Toxicology, Ruhr University Bochum, D 44780 Bochum, Germany

##### **Correspondence:** Alexander Kollau **(**alexander.kollau@uni-graz.at**)**

**Fig. 6:**
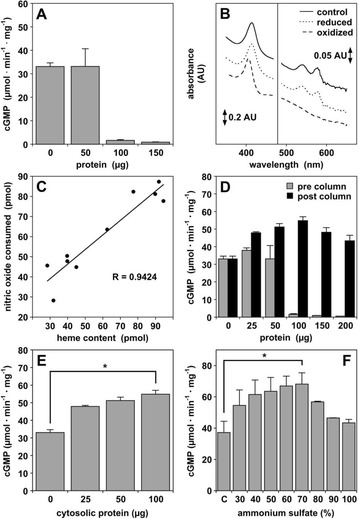
**(Abstract A46).** NO-scavenging and sGC-activating properties of PCA cytosols. **a** Protein-dependent reduction of NO-stimulated cGMP formation in the presence of cytosols **b**) UV/Vis spectra of cytosols under various conditions **c**) Correlation of NO consumption and heme content of cytosols **d**) Effect of native or hemoglobin-depleted cytosols on NO-stimulated cGMP formation **e**) Protein-dependent increase of NO-stimulated cGMP formation in the presence of hemoglobin-depleted cytosols **f**) Selective precipitation of the activating factor by ammonium sulfate, * p<0.05

As a central part of the NO/cGMP signaling pathway soluble guanylate cyclase (sGC) has become a promising therapeutic target in the treatment of cardiovascular diseases [1] and is thus subject of intensive research. In the course of studies on bioactivation of the anti-anginal drug nitroglycerin in blood vessels we performed co-incubation experiments of purified sGC from bovine lung with cytosols from porcine coronary arteries. Cytosolic preparations considerably diminished NO-stimulated sGC activity (Fig. 6a). This effect persisted in the presence of the phospodiesterase inhibitor isobutylmethylxanthine, a protease inhibitor cocktail, and superoxide dismutase, excluding cGMP hydrolysis, proteolysis of sGC, and NO inactivation by superoxide as culprits. Spectroscopic analysis of cytosols revealed the presence a heme-containing protein with characteristic Soret and α/β bands (Fig. 6b). Using Western blot and LC-MS/MS analysis we identified the protein as hemoglobin, which is known to effectively scavenge NO [2]. Heme-mediated NO scavenging by cytosolic preparations was confirmed with an NO-sensitive electrode (Fig. 6c). Removal of hemoglobin by haptoglobin affinity chromatography revealed an unexpected increase of NO-stimulated sGC activity by cytosols (Fig. 6d). The effect was dependent on the amount of cytosolic protein added (Fig. 6e), precipitable by ammonium sulfate (Fig. 6f), and sensitive to heat and organic solvents. Preliminary results indicate that this protein, termed sGC-activating factor (sGC-AF) can be recovered by gel filtration. Work on isolation and identification of sGC-AF is ongoing in our laboratory. Our findings demonstrate expression of an as yet unidentified protein in porcine coronary arteries that activates sGC, resulting in increased v_max_ of the maximally NO-stimulated enzyme.

**Conflict of interest**

*None*

**References:**

1. Stasch JP, Pacher P, Evgenov OV: **Soluble guanylate cyclase as an emerging therapeutic target in cardiopulmonary disease**. *Circulation* 2011, **123**(20):2263-2273.

2. Ford PC, Pereira JCM, Miranda KM: **Mechanisms of nitric oxide reactions mediated by biologically relevant metal centers**. *Struct Bond* 2013, **154**:99-136.

## A47 cGMP induction suppresses pancreatic cancer stem cell properties

### Motofumi Kumazoe^1,2,†^, Mika Takai ^1,†^, Chieri Takeuchi^1^, Mai Kadomatsu^1^, Shun Hiroi^1^, Kanako Takamatsu^1^, Takashi Nojiri^2^, Kenji Kangawa^2^, Hirofumi Tachibana^1^

#### ^1^Division of Applied Biological Chemistry, Department of Bioscience and Biotechnology, Faculty of Agriculture, Kyushu University, 6-10-1 Hakozaki, Higashi-ku, 812-8581 Fukuoka, Japan; ^2^Department of Biochemistry, National Cerebral and Cardiovascular Center Research Institute, 5-7-1 Fujishiro-dai, Suita-City, 565-8565 Osaka, Japan

##### **Correspondence:** Hirofumi Tachibana **(**tatibana@agr.kyushu-u.ac.jp**)**; Motofumi Kumazoe

^†^These authors contributed equally to this work.

**Clinical background:**

**Fig. 7:**
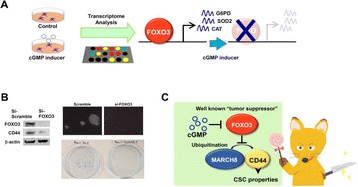
**(Abstract A47).** cGMP induction suppresses pancreatic cancer stem cell properties. **a** cGMP induction suppressed FOXO3. **b** Silencing of FOXO3 suppressed CSC properties. **c** cGMP induction suppresses pancreatic cancer stem cell properties

Several reports indicate cancer stem cells (CSCs) may act a central role in pancreatic ductal adenocarcinoma (PDAC) metastasis and recurrence. However, no clinical available approach is established. cGMP is known as a second messenger plays a crucial role in penile erection and vascular homeostasis. We previously reported that cGMP acts the signal mediator in 67-kDa laminin receptor-dependent multiple myeloma cell death^1^. Here we show cGMP induction drastically suppressed CSC properties in three different PDAC cell lines including Panc-1, Miapaca-2 and BxPC-3 cells. Our microarray analysis showed cGMP-induction suppressed FOXO3 (see Fig. 7a). We also showed FOXO3 are strongly expressed in CD44^+^ cells^2^. Surprisingly, FOXO3, known as the tumour suppressor plays the crucial role in the maintenance of CSC properties and FOXO3 knock down strongly suppressed CSC properties by downregulation of CD44, the essential protein for CSC properties (see Fig. 7b, c). The same results were obtained in vivo^2^.

**Conclusion:**

Considering the FOXO3 knock out mice did not change survival period, cGMP induction and FOXO3 suppression could be the novel strategies for PDAC therapy.

**Conflict of interest**

*The authors declare that they do not have a potentially perceived conflict of interest.*

**References:**

[1]. M Kumazoe, K Sugihara, S Tsukamoto, Y Huang, Y Tsurudome, T Suzuki, Y Suemasu, N Ueda, S Yamashita, Y Kim, K Yamada, H Tachibana: **67-kDa laminin receptor increases cGMP to induce cancer-selective apoptosis.** *J. Clin. Invest.* 2013, **123**: 787-799

[2]. M Kumazoe, M Takai, J Bae, S Hiroi, Y Huang, K Takamatsu, Y Won, M Yamashita, S Hidaka, S Yamashita, S Yamada, M Murata, S Tsukamoto, H Tachibana: **FOXO3 is essential for CD44 expression in pancreatic cancer cells.** *Oncogene* 2016, *in press*.

## A48 Formation of nitric oxide by aldehyde dehydrogenase-2 is necessary and sufficient for nitroglycerin-induced activation of soluble guanylate cyclase

### Marissa Opelt^1^, Emrah Eroglu^2^, Markus Waldeck-Weiermair^2^, Michael Russwurm^3^, Doris Koesling^3^, Roland Malli^2^, Wolfgang F. Graier^2^, John T. Fassett^1^, Astrid Schrammel^1^, Bernd Mayer^1^

#### ^1^Institute of Pharmaceutical Sciences, Department of Pharmacology and Toxicology, University of Graz, Graz, Austria; ^2^Institute of Molecular Biology and Biochemistry, Center of Molecular Medicine, Medical University of Graz, Graz, Austria; ^3^Department of Pharmacology and Toxicology, Ruhr University Bochum, Bochum, Germany

##### **Correspondence:** Bernd Mayer **(**bernhard-michael.mayer@uni-graz.at**)**

**Fig. 8:**
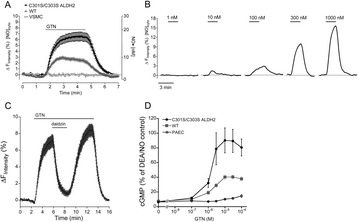
**(Abstract A48).** GTN-induced NO formation and cGMP accumulation in intact cells. **a**. NO release over time was measured in VSMC infected with C-geNOp alone or in combination with either WT or C301S/C303S ALDH2 in response to 1 μM GTN **b.** Representative curves showing NO release in response to cumulative increasing GTN concentrations (10 nM – 1 μM) in VSMC expressing C301S/C303S ALDH2 **c.** Effect of 0.2 mM daidzin on GTN-derived NO formation **d.** GTN-induced cGMP accumulation in non-infected and infected (WT or C301S/C303S ALDH2) porcine aortic endothelial cells

Aldehyde dehydrogenase-2 (ALDH2) catalyzes vascular bioactivation of the antianginal drug nitroglycerin (GTN), resulting in 3´,5´-cyclic guanosine monophosphate (cGMP)-mediated vasodilation through activation of soluble guanylate cyclase (sGC). We have previously shown that a minor reaction of ALDH2-catalyzed GTN bioconversion, accounting for about 5% of the main clearance-based turnover yielding inorganic nitrite, results in direct nitric oxide (NO) formation and concluded that this minor pathway could provide the link between vascular GTN metabolism and activation of sGC [1]. However, the biological significance of NO formation by purified ALDH2 is questioned by the persistent failure to detect release of NO in vascular tissue and cells exposed to therapeutically relevant GTN concentrations (<1 μM). To address this issue, we took advantage of a novel, highly sensitive genetically encoded fluorescent NO probe (C-geNOp) that enables real-time monitoring of intracellular NO formation in cultured vascular smooth muscle cells (VSMC) expressing either wild-type ALDH2 or a mutant (C301S/C303S ALDH2) that reduces GTN to NO but lacks clearance-based GTN denitration activity. Addition of 1 μM GTN to VSMC expressing either wild-type or C301S/C303S ALDH2 resulted in a pronounced increase in intracellular NO, with maximal concentrations of 7 and 17 nM, respectively (Fig. 8a). In VSMC expressing C301S/C303S ALDH2 NO formation was detectable at therapeutically relevant submicromolar concentrations (10 nM – 1 μM) of the nitrate (Fig. 8b). The selective ALDH2 inhibitor daidzin (0.2 mM) completely inhibited GTN-derived NO formation in a rapid and reversible manner (Fig. 8c). Formation of GTN-derived NO correlated well with activation of purified sGC in VSMC lysates as well as cGMP accumulation in cultured porcine aortic endothelial cells that had been infected with wild-type or C301S/C303S ALDH2 (Fig. 8d). Our findings demonstrate that ALDH2-catalyzed NO formation is necessary and sufficient for vascular bioactivation of GTN.

**Conflict of interest**

*None*

**Reference:**

[1]. Wenzl MV, Beretta M, Griesberger M, Russwurm M, Koesling D, Schmidt K, Mayer B and Gorren AC: **Site-directed mutagenesis of aldehyde dehydrogenase-2 suggests three distinct pathways of nitroglycerin biotransformation.** *Mol Pharmacol* 2011, **80:** 258-266

## A49 C-type natriuretic peptide increases titin phosphorylation and decreases passive stiffness in rat cardiomyocytes

### Selene J. Sollie ^1,2^, Lise Román Moltzau^1,2^, Maria Hernandez-Valladares^3^, Frode Berven^3^, Finn Olav Levy^1,2^, Kjetil W. Andressen^1,2^

#### ^1^Department of Pharmacology, Faculty of Medicine, University of Oslo and Oslo University Hospital, Oslo, Norway; ^2^Center for Heart Failure Research, Faculty of Medicine, University of Oslo, Oslo, Norway; ^3^Department of Biomedicine, Faculty of Medicine and Dentistry, University of Bergen, Bergen, Norway

##### **Correspondence:** Lise Román Moltzau **(**l.r.moltzau@medisin.uio.no**)**

Diastolic heart failure, often referred to as HFpEF (heart failure with preserved ejection fraction) accounts for about 50% of heart failure cases. Currently, there is no good treatment for this condition. Changes associated with HFpEF are impaired left ventricular (LV) filling, increased LV stiffness and altered expression and phosphorylation of the elastic sarcomeric protein titin. The main function of titin is to keep the structural integrity of the sarcomere intact, but also function as an elastic spring, contributing to passive tension development within the myocytes. Protein kinase G (PKG) has earlier been shown to phosphorylate titin and decrease passive stiffness in human myofibrils, thus allowing more compliant cardiomyocytes. PKG can be activated by cGMP increased either by nitric oxide (NO) through the activation of soluble guanylyl cyclase (sGC) or by the natriuretic peptides (NPs) through the activation of the particulate guanylyl cyclases NPR-A (GC-A) or NPR-B (GC-B). The aim of this study was to determine which receptor-mediated signaling pathways induce PKG-dependent titin phosphorylation and reduce passive force development. Cyclic GMP levels, titin phosphorylation, phosphomapping of the titin protein and passive tension were performed in isolated rat cardiomyocytes. C-type NP (CNP), stimulating NPR-B, brain NP (BNP) stimulating NPR-A and SNAP (NO donor) stimulating sGC all increased cGMP. However, only CNP increased titin phosphorylation and decreased passive tension in single cardiomyocytes. Thus, despite increasing cGMP, activating NPR-A or sGC did not reduce cardiomyocyte stiffness, whereas NPR-B activation did. This indicates compartmentation of the different signaling pathways increasing cGMP. Further, our results suggest NPR-B as a potential target to improve LV filling by decreasing LV stiffness in HFpEF patients.

***Competing interest***

*No conflicts of interest.*

## A50 Endothelial ANP-GC-A signaling protects against pre-metastatic niche formation in tumor-bearing mice

### Takashi Nojiri^1,2^, Takeshi Tokudome^1^, Motofumi Kumazoe^1^, Miki Arai ^3,4^, Yutaka Suzuki^4^, Koichi Miura^1^, Jun Hino^1^, Hiroshi Hosoda^5^, Mikiya Miyazato^1^, Meinoshin Okumura^2^, Shinpei Kawaoka^3^, Kenji Kangawa^1^

#### ^1^Department of Biochemistry, National Cerebral and Cardiovascular Center Research Institute, Suita, Osaka, Japan; ^2^Department of General Thoracic Surgery, Osaka University Graduate School of Medicine, Suita, Osaka, Japan; ^3^Advanced Telecommunications Research Institute International (ATR), The Thomas N. Sato BioMEC-X Laboratories, Kyoto, Japan; ^4^The University of Tokyo, graduate school of frontier science, Kashiwa, Japan; ^5^Department of Regenerative Medicine and Tissue Engineering, National Cerebral and Cardiovascular Center Research Institute, Suita, Osaka, Japan

##### **Correspondence:** Takashi Nojiri **(**nojiri@ri.ncvc.go.jp**)**

**Clinical background:**

Drugging cancer metastases, extremely deadly diseases, has been on high demand but still challenging. Metastasis is a step-wise complex phenomenon including dynamic physiological alternations of both cancers and host. Cancers set up a favorable environment (soil) in distant organs for disseminated cancer cells (seeds) to efficiently metastasize: pre-metastatic niche hypothesis. Pre-metastatic niche is a possible target for targeting metastasis. We have previously reported that atrial natriuretic peptide (ANP), an endogenous peptide produced by the heart, inhibits hematogenous cancer metastasis through vascular endothelial cells. We showed that ANP inhibits the tumor cell adhesion to the vascular endothelium by suppressing E-selectin expression, which has a central role in the adhesion of tumor cells to endothelial cells. In this study, we show that ANP suppresses pre-metastatic niche formation and following metastasis when pharmacologically supplied in tumor-bearing mice. ANP administration reduced the lung metastasis in the mouse models of 4T1 breast cancer and colon26 cancer. Comprehensive RNA-seq analyses using the 4T1 and Lewis Lung Carcinoma (LLC) models demonstrated that gene expression changes characteristics of pre-metastatic niche in the lung were suppressed by ANP treatment. The lung of mice overexpressing GC-A, a receptor for ANP, in endothelial cells, was resistant to pre-metastatic niche formation than the WT lung. Neither ANP administration nor GC-A overexpression had a hazardous effect on the lung gene expressions in a cancer-free condition. In summary, we showed that the endothelial ANP-GC-A signaling attenuates pre-metastatic niche formation in the lung in a context-specific manner.

**Conclusion:**

Altogether, we concluded that ANP-GC-A signaling as a promising target for controlling hematogenous cancer metastasis and pre-metastatic niche formation in various types of cancers.

## A51 How do DRG axons bifurcate? Roles of PDE2, Npr3 and crosstalk of cGMP and Ca^2+^ signaling

### Stefanie Peters^1^, Hannes Schmidt^2,5^, B. Selin Kenet^1,3^, Sarah Helena Nies^1^, Katharina Frank^2,4^, Lai Wen^1^, Fritz G. Rathjen^2^ and Robert Feil^1^

#### ^1^Interfakultäres Institut für Biochemie, University of Tübingen, Tübingen, Germany; ^2^Max-Delbrück-Centrum für Molekulare Medizin, Berlin, Germany; ^3^Present address: Institut für Zellbiologie des Nervensystems, TU München, Germany; ^4^Present address: Institut für Immunologie, BioMedical Center, LMU, Planegg-Martinsried, Germany; ^5^Present address: Interfakultäres Institut für Biochemie, University of Tübingen, Tübingen, Germany

##### **Correspondence:** Stefanie Peters **(**stefanie.peters@uni-tuebingen.de**)**

Axonal branching is essential for correct formation of neuronal networks and subsequent transmission of information throughout the body. It is well known that a cGMP signaling cascade consisting of C-type natriuretic peptide (CNP), guanylate cyclase B (GC-B, also known as Npr2) and cGMP-dependent protein kinase I (cGKI) is crucial for axon bifurcation of dorsal root ganglia (DRG) sensory neurons and cranial sensory ganglia neurons during mouse embryonic development. In this study, we investigated (1) whether other components of the cGMP signaling pathway, namely phosphodiesterases (PDEs) and the natriuretic peptide “clearance” receptor Npr3, are also involved in the bifurcation of embryonic DRG neurons and (2) whether CNP-evoked increase of cGMP regulates the intracellular Ca^**2+**^ level in DRG neurons. RT-PCR screens, in situ hybridization, and fluorescence resonance energy transfer-based live-cell cGMP imaging revealed PDE2A as the major enzyme responsible for the degradation of CNP-induced cGMP in embryonic DRG neurons. Interestingly, cGMP measurements and DiI labeling of PDE2A knockout embryos indicated that an elevated cGMP level does not disturb axon bifurcation of DRG neurons. Npr3 is expressed in cells of the roof and floor plate of the spinal cord as well as in the dorsal roots of E12.5 mouse embryos. Npr3 likely acts as a clearance receptor for CNP and might, therefore, lower the activity of the CNP/GC-B/cGMP cascade in DRGs. In the absence of Npr3, and presumably higher cGMP levels in DRG neurons, a small proportion of sensory axons showed deficits in bifurcation by turning either in rostral or caudal direction, while most axons branched normally. Fura-2-based Ca^2+^ imaging revealed that acetylcholine (ACh) and ATP induce Ca^2+^ transients in somata and growth cones of E12.5 DRG neurons, respectively. Simultaneous imaging of cGMP and Ca^2+^ signals showed that the ACh/ATP-induced Ca^2+^ transients were strongly suppressed by CNP-induced cGMP. The suppressive effect of cGMP on agonist-induced Ca^2+^ signals was absent in DRG neurons of cGKI knockout mice demonstrating that the cGMP/Ca^2+^ crosstalk is mediated by cGKI. Altogether, our study indicates that DRG sensory axon bifurcation tolerates increased cGMP levels in PDE2A or Npr3 knockout mice. Furthermore, we discovered a crosstalk between cGMP and Ca^2+^ signaling in embryonic DRG neurons that might provide a mechanistic basis for axon bifurcation.

**Funding**

*This work was supported by DFG grant FOR 2060.*

***Competing interest***

*The authors declare no competing financial interests.*

## A52 Natural compounds as inhibitors of soluble guanylate cyclase

### Olga N. Petrova, Isabelle Lamarre and Michel Négrerie

#### Laboratory for Optics and Biosciences, Ecole Polytechnique, Palaiseau, France

##### **Correspondence:** Olga N. Petrova **(**olga.petrova@polytechnique.edu**)**

**Background:**

Soluble guanylate cyclase (sGC) is an enzyme involved in signal transduction which catalyzes the formation of cGMP from GTP and is activated by the binding of NO to its heme group. The NO – cGMP – sGC signaling pathway participates in a wide range of regulating processes for intracellular metabolism: regulation of blood pressure through smooth muscle relaxation and vasodilation, apoptosis, signaling in tumor cell proliferation, angiogenesis, immune response and inflammation. This study was aimed at discovering sGC inhibitors and to understand their binding mechanism.

The effect of 200 natural compounds from a chemical library, including hypericin and hypocrellin (two potential agents for photodynamic therapy), have been investigated on sGC activity.

Subsequently, hypericin, hypocrellin and four other natural compounds from plants and fungi: stictic acid, violastyrene, 2-hydroxy-3,5,8-triaceto-1,4-naphthoquinone (HTANQ), 3,6-dibromo-purpurogallin (DBPG) were shown to inhibit sGC in vitro and in HUVEC cells.

Using immunoenzymatic assay of sGC activity, we measured the cross effects of these inhibitors with NO and with the activator BAY 41-2272.

**Fig. 9:**
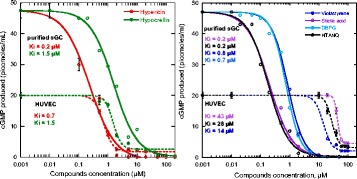
**(Abstract A52).** Inhibition of purified sGC (solid curves) and sGC in HUVEC cells (dotted curves) by the natural compounds. Density of cells was 5.6 × 10^5^ cells/ml. Activity was measured as synthesized cGMP in the presence of the NO-donor nitroprusside (300 μM)

The inhibition constant (Ki) for cGMP synthesis (Fig. 9) measured in vitro on purified sGC (hypericin: 0.3 μM; hypocrellin: 0.6 μM, stictic acid: 0.2 μM, violastyrene: 0.8 μM, HTANQ: 0.2 μM, DPG: 0.7 μM) was lower than that measured in vivo on HUVEC (hypericin: 0.7 μM; hypocrellin: 1.5 μM, stictic acid: 43 μM, violastyrene: 14 μM, HTANQ: 28 μM). We noted that DBPG does not pass through the HUVEC cell membrane.

In vitro, the presence of the NO-independent activator BAY 41-2272 bound to sGC does not change the inhibition induced by these compounds. In vivo, BAY 41-2272 reduces the hypericin/hypocrellin induced inhibition of sGC but does not influence the inhibition constant of compounds from plants and fungi.

By surface plasmon resonance, we showed irreversible binding of hypericin, hypocrellin and DBPG to guanylate cyclase in comparison with other studied inhibitors.

**Conclusion:**

Our results demonstrate that the discovered inhibitors are allosteric modulators which bind neither to the heme, nor to the catalytic and activator sites, revealing a new class of pharmacological compounds for sGC.

***Competing interest***

*The authors declare no conflict of interest.*

**Funding**

*Fellowship was provided to O.N.Petrova by «Initiative Interdisciplinaire from Université Paris-Saclay.*

## A53 Dephosphorylation is the Mechanism of Fibroblast Growth Factor Inhibition of Guanylyl Cyclase-B

### Jerid W. Robinson^1^, Jeremy R. Egbert^2^, Julia Davydova^3^, Laurinda A. Jaffe^2^, and Lincoln R. Potter^1,4^

#### ^1^Department of Biochemistry, Molecular Biology, and Biophysics, University of Minnesota, Minneapolis, MN, USA; ^2^Department of Cell Biology, University of Connecticut Health Center, Farmington, CT, USA; ^3^Department of Surgery, University of Minnesota, Minneapolis, MN, USA; ^4^Department of Pharmacology, University of Minnesota, Minneapolis, MN, USA

##### **Correspondence:** Jerid W. Robinson **(**robin768@umn.edu**)**

Both activating mutations in fibroblast growth factor receptor 3 (FGFR3) and inactivating mutations of guanylyl cyclase B (GC-B), also called NPRB or NPR2, cause human dwarfism. Reciprocal regulation of the two pathways has been reported, but how FGFR3 inhibits GC-B is not known. Here, we demonstrate that FGF2 stimulation of FGFR3 causes a rapid, potent, and reversible inhibition of endogenous GC-B enzymatic activity in rat chondrosarcoma cells. FGF2 concomitantly reduced the phosphate content and enzymatic activity of GC-B, and the serine and threonine phosphatase inhibitor, cantharidin, blocked the FGF2-dependent reduction in the enzymatic activity of GC-B. A mutated form of GC-B containing glutamate substitutions for all confirmed and putative phosphorylation sites, such that it cannot be inactivated by dephosphorylation, was not inactivated by FGF2. We conclude that FGF2 activation of FGFR3 inactivates GC-B by a reversible dephosphorylation mechanism and suggest that dephosphorylation is a universal inactivation mechanism commonly employed by natriuretic peptide-stimulated guanylyl cyclase receptors.

## A54 Prevention of Guanylyl Cyclase-B dephosphorylation increases long bone content, density, and strength

### Jerid W. Robinson^1^, Nicholas Blixt^2^, Leia C. Shuhaibar^3^, Gordon L. Warren^4^, Kim C. Mansky^2^, Laurinda A. Jaffe^3^, and Lincoln R. Potter^1,5^

#### ^1^Department of Biochemistry, Molecular Biology, and Biophysics, University of Minnesota, Minneapolis, MN, USA; ^2^Department of Developmental and Surgical Sciences, University of Minnesota, Minneapolis, MN, USA; ^3^Department of Cell Biology, University of Connecticut Health Center, Farmington, CT, USA; ^4^Department of Physical Therapy, Georgia State University, Atlanta, GA, USA; ^5^Department of Pharmacology, University of Minnesota, Minneapolis, MN, USA

##### **Correspondence:** Jerid W. Robinson **(**robin768@umn.edu**)**

Activating and inactivating mutations in guanylyl cyclase (GC)-B cause skeletal overgrowth and dwarfism, respectively. C-type natriuretic peptide (CNP) dependent stimulation of GC-B requires receptor phosphorylation on multiple residues. A knock-in mouse expressing a mutant GC-B where all known phosphorylation sites were mutated to glutamate to mimic a constitutively phosphorylated enzyme, called GC-B^7E/7E^, was created that has a longer appendicular skeleton. Here, we show that GC-B^7E/7E^ mice have 30% more trabecular bone volume and number, 10% greater cortical bone thickness and area, and 20% more bone mineral density at 9 weeks compared to GC-B^WT/WT^ mice. 3-point bending assays demonstrated that 9 week bones from GC-B^7E/7E^ mice have 35% increased strength and stiffness and 65% increased toughness compared to wild-type mice, all of which can be attributed to the increased cortical bone. At 16 weeks, cortical area and bone mineral density are still 14% and 22% greater in GC-B^7E/7E^ mice. However, no difference in trabecular bone volume and number was observed between the two mouse lines at 16 weeks, due to GC-B^7E/7E^ trabecular bone loss. Why the trabecular bone increases observed at 9 weeks are absent at 16 weeks is not known. These data indicate that blocking GC-B dephosphorylation and inactivation increases cortical and trabecular content, density, stiffness, and strength of long bones at 9 weeks. At 16 weeks the cortical gains are diminished and the trabecular gains are lost. We suggest that therapeutics that increase GC-B phosphorylation may decrease fracture healing time and/or prevent post-menopausal osteoporotic bone loss.

***Competing interest***

*On behalf of all authors, the corresponding author states that there is no conflict of interest*

## A55 Evaluation of Kidney Protection of Soluble Guanylate Cyclase (sGC) Modulators in a Novel, Fast Mouse Model

### Simone Romoli^1^, Tobias Bauch^1^, Karoline Dröbner^1^ and Frank Eitner^1^

#### ^1^Cardiovascular Research II, Therapeutic Research Group, Drug Discovery, Bayer AG, Wuppertal, Germany

##### **Correspondence:** Simone Romoli **(**simone.romoli@bayer.com**)**

**Introduction:**

Treatment with sGC stimulators or activators has resulted in kidney protection in a variety of preclinical animal models. A key surrogate for efficacy in treating chronic kidney disease progression is albuminuria. Currently, common kidney *in vivo* models with albuminuria as primary endpoint are chronic models, with duration of up to several months. This is a significant hurdle in the search for novel kidney protective agents like sGC modulators.

**Methods:**

We developed a new, short-term model that will enable us to study novel drugs with the potential ability to reduce albuminuria. Urine was collected for four hours from wildtype C57/Bl6J mice that had received two simultaneous injections of angiotensin II (AngII, 10 μg *i.v.* + 10 μg *s.c.*). Albuminuria was measured as albumin-creatinine ration (ACR) with Roche Cobas I400. Group sizes ranged from 5-9 mice. All treatments were given orally prior to AngII. Data are reported as means +/- SD.

**Results:**

**Fig. 10:**
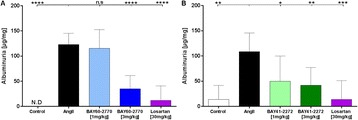
**(Abstract A55).** sGC modulators reduce albuminuria. Albuminuria was significantly increased within 4h after AngII bolus injections. The albuminuria ratio was significantly reduced in the experimental group treated with sGC activator BAY-602770 to a mean 35±26 μg/mg at the dose 3 mg/kg (**a**). Treatment with the sGC stimulator BAY 41-2272 significantly reduced the albuminuria to a mean 50± 49 μg/mg and 42± 35 μg/mg in the respective doses of 1 and 3mg/kg (**b**). Losartan treated animals showed a highly significant reduction of AngII induced albuminuria by around 90%, N.D = not detectable albuminuria, Data are mean ± SD */**/***/****= significant with p<0.05/0.01/0.001/0.0001 vs. AngII (One-way ANOVA followed by Dunnett´s Multiple Comparison post-hoc analysis)

Application of AngII resulted in a robust and significant albuminuria (mean ACR 123 μg/mg vs. 13 μg/mg in control mice). No albuminuria was detectable 24h after AngII application. We investigated the sGC stimulator BAY 41-2272 and the sGC activator BAY 60-2770 in this model. Both compounds had demonstrated significant anti-proteinuric effects in different long-term studies (data not shown). Treatment with 3 mg/kg BAY 60-2770 reduced albuminuria by 71% compared with the control group (Fig. 10a). In a second experiment, the sGC stimulator BAY 41-2272 reduced albuminuria significantly by 54% and 62% at 1 and 3 mg/kg respectively (Fig. 10b). Losartan was used in both studies as positive control.

**Conclusion:**

Our new mouse model was capable of demonstrating albuminuria-reducing effects of select sGC modulators within a few hours experimental setting. We propose the use of this model as robust and fast platform for the screening of novel drugs in albuminuric diseases.

***Competing interest***

*All authors are full-time employees of Bayer AG.*

## A56 Pharmacological activation of the soluble guanylate cyclase inhibits the progression of pressure overload-induced pathological myocardial hypertrophy in an aortic banded rat model

### Mihály Ruppert^1,2^, Tamás Radovits^2^, Sevil Korkmaz-Icöz^1^, Shiliang Li^1^, Péter Hegedűs^1^, Sivakanan Loganathan^1^, Balázs Tamás Németh^2^, Attila Oláh^2^, Csaba Mátyás^2^, Kálmán Benke^2^, Béla Merkely^2^, Matthias Karck^1^, Gábor Szabó^1^

#### ^1^Department of Cardiac Surgery, University of Heidelberg, Heidelberg, Germany; ^2^Heart and Vascular Center, Semmelweis University, Budapest, Hungary

##### **Correspondence:** Mihály Ruppert **(**ruppertmis@gmail.com**)**

**Clinical background:**

In contrast to the previous scientific views, recent studies have indicated that pressure overload-induced left ventricular myocardial hypertrophy (LVH) is pathological in principal. Therefore in the last decades, understanding the molecular basis of LVH in order to prevent its manifestation has been the focus of interest. As a result, the soluble guanylate cyclase (sGC) - cyclic guanosine monophosphate (cGMP) – protein kinase G pathway has been identified as a potent anti-hypertrophic signaling, which may offer possibilities for pharmaceutical interventions.

**Purpose:**

In the present study the novel sGC activator Cinaciguat was tested in a rodent model of pressure overload-induced LVH.

**Methods:**

Constriction of the abdominal aorta at the suprarenal level (aortic banding; AB) was performed to establish hypertension in the proximal part of the aorta for 6 or 12 weeks. Sham operated animals were used as controls. From the 7th to the 12th experimental week 10mg/kg/day cinaciguat (Cin) or placebo (Co) was administered p.o. Serial echocardiography and invasive pressure-volume analysis were carried out to assess the morphological and functional alterations of the left ventricle. Furthermore, histological and molecular biological measurements were performed.

**Results:**

Activation of the sGC enzyme by cinaciguat effectively reduced the hypertrophic remodeling of the myocardium as evidenced by decreased heart weight-to-tibial length ratio (0.57±0.02 vs. 0.48±0.02g/cm, p<0.05 AB 12th week-Co week vs. AB 12th week-Cin), decreased cardiomyocyte diameter (23.94±0.59 vs. 20.02±0.20μm, p<0.05 AB 12th week-Co week vs. AB 12th week-Cin) and reduced myocardial expression levels of atrial natriuretic peptide and β/α-myosin heavy chain ratio. In addition, the cinaciguat treatment also inhibited the increased collagen accumulation in the interstitium (Masson’s score: 1.7±0.2 vs. 1.2±0.1, P<0.05 AB 12week-Co vs. AB 12week-Cin) and provided protection against the nitro-oxidative stress. All of these beneficial structural and molecular alterations manifested in significantly improved cardiac function (ejection fraction: 47.4±2.7 vs. 63.7±2.4%, P<0.05 AB 12week-Co vs. AB 12week-Cin).

**Conclusion:**

Here we provided evidence, that chronic activation of the sGC enzyme might represent a novel therapy in case of pressure overload-induced LVH and cardiac dysfunction.

***Competing interest***

*The authors declare that they do not have a potentially perceived conflict of interest.*

## A57 Optogenetic guided synthesis of cGMP through Rhodopsin-guanylyl cyclases

### Ulrike Scheib^1^, Matthias Broser^1^, Shatanik Mukherjee^1^, Katja Stehfest^1^, Christine E. Gee^2^, Heinz G. Körschen^3^, Thomas G. Oertner^2^, and Peter Hegemann^1^

#### ^1^Humboldt Universität zu Berlin, Institute for Biology, Experimental Biophysics, Berlin, Germany; ^2^University Medical Center Hamburg-Eppendorf, Center for Molecular Neurobiology Hamburg, Institute for Synaptic Physiology, Hamburg, Germany; ^3^Center of Advanced European Studies and Research (caesar) Bonn, Department: Molecular Sensory Systems, Bonn, Germany

##### **Correspondence:** Ulrike Scheib **(**ulrike.scheib@hu-berlin.de**)**

**Fig. 11:**
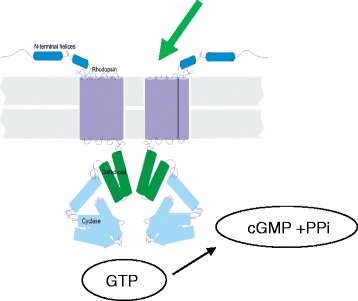
**(Abstract A57).** Model of the Rhodopsin-guanylyl cyclase. Activation with green light (500 ms) leads to the production of cGMP

**Background**:

Cyclic GMP (cGMP) is a central second messenger, regulating a multitude of cellular and physiological processes, like the visual cascade, electrolyte homeostasis, and smooth muscle relaxation (Lucas et al., 2000). Recently, a rhodopsin-guanylyl cyclase (RhGC) from the aquatic fungus *Blastocladiella Emersonii* was characterized (Scheib et al., 2015, Gao et al., 2015), which produces cGMP upon a short flash of green light and can be expressed in different mammalian cell types (Fig. 11). Due to the direct linkage between the rhodopsin and the cyclase domain, RhGC represents the first member of a novel class of enzyme linked rhodopsin (2). Here, we further characterize RhGC of *Blastocladiella Emersonii* (BE) enzymatically and with the help of X-ray crystallography. Additionally, we found an orthologue variant, RhGC from Catenaria Anguillulae with improved photo-stability, which can also be functionally expressed in different eukaryotic cell types. Like for the BE version, the onset of cGMP production was rapid and cGMP signals could be induced repeatedly, while no dark activity was apparent.

**Conclusion:**

Rhodopsin-guanylyl cyclases are a versatile optogenetic tool to analyze cGMP-dependent signaling processes in cell biology and the neurosciences.

**Conflict of interest**

The authors declare that they do not have a potentially perceived conflict of interest.

**References:**

Lucas, K. A., Pitari, G. M., Kazerounian, S., Ruiz-Stewart, I., Park, J., Schulz, S., … Waldman, S. A. (2000). Guanylyl cyclases and signaling by cyclic GMP. *Pharmacological Reviews*, *52*(3), 375–414. Retrieved from http://www.ncbi.nlm.nih.gov/pubmed/10977868

Scheib, U., Stehfest, K., Gee, C. E., Körschen, H. G., Fudim, R., Oertner, T. G., & Hegemann, P. (2015). The rhodopsin-guanylyl cyclase of the aquatic fungus Blastocladiella emersonii enables fast optical control of cGMP signaling. *Science Signaling*, *8*(389), rs8. doi:10.1126/scisignal.aab0611

Gao, S., Nagpal, J., Schneider, M. W., Kozjak-Pavlovic, V., Nagel, G., & Gottschalk, A. (2015). Optogenetic manipulation of cGMP in cells and animals by the tightly light-regulated guanylyl-cyclase opsin CyclOp. *Nature Communications*, *6*, 8046. doi:10.1038/ncomms9046

## A58 Phosphorylation of juxtamembrane serine and threonine residues of guanylate cyclase B is essential for sensory axon bifurcation

### Hannes Schmidt^1,2^, Deborah M. Dickey^3^, Alexandre Dumoulin^2^, Ralf Kühn^2^, Laurinda Jaffe^4^, Lincoln R Potter^3^, Fritz G Rathjen^2^

#### ^1^Interfaculty Institute of Biochemistry, University of Tübingen, Tübingen, Germany; ^2^Max Delbrück Center for Molecular Medicine in the Helmholtz Association, Berlin, Germany; ^3^Department of Biochemistry, University of Minnesota, Minneapolis, Minnesota, USA; ^4^Department of Cell Biology, University of Connecticut Health Center, Farmington, Connecticut, USA

##### **Correspondence:** Hannes Schmidt **(**hannes.schmidt@uni-tuebingen.de**)**

cGMP signalling activated by C-type natriuretic peptide (CNP) and its receptor, the particulate guanylate cyclase B (GC-B, also known as Npr2), is critically involved in the regulation of endochondral ossification, the maturation of oocytes and the homeostasis of arterial blood pressure. Biallelic loss-of-function mutations in the human NPR2 gene result in acromesomelic dysplasia type Maroteaux (AMDM), a skeletal dysplasia with an extremely short and disproportionate stature. Similar to human patients, constitutive GC-B-deficient mice are characterized by a dwarfed phenotype. Adding to the physiological functions of the CNP/GC-B-system, our studies revealed that CNP/GC-B-induced cGMP signalling via cGMP-dependent protein kinase I controls bifurcation of sensory axons in the embryonic spinal cord or brain stem. In the absence of any one of these components the axons of neurons from the dorsal root ganglia or cranial sensory ganglia no longer bifurcate and instead turn either in an ascending or descending direction.

GC-B forms a homodimeric transmembrane receptor composed of an amino-terminal extracellular ligand-binding-domain, a single membrane-spanning region, and an intracellular kinase homology domain (KHD) followed by a dimerization segment and a carboxyl-terminal guanylate cyclase domain. Previous studies reported that phosphorylation of seven regulatory serine and threonine residues in the KHD of GC-B is a prerequisite for ligand-induced cGMP formation. However, whether phosphorylation of these sites in GC-B is also required in physiological systems needs to be confirmed.

To address this question we applied CRISPR/Cas9 genome editing to generate a knock-in mouse mutant in which each site was substituted by alanine (GC-B-7A), resulting in a nonphosphorylatable enzyme. Biochemical analysis of guanylate cyclase activity in tissues from GC-B^7A/7A^ mice demonstrated a significant reduction in cGMP levels generated upon stimulation by CNP. Interestingly, we also detected a decrease in guanylate cyclase activity in the heterozygous GC-B^WT/7A^ mice as compared to wild type mice which might be due to a dominant negative effect of the mutant form of the receptor. GC-B^7A/7A^ mice exhibit a dwarfed phenotype as well as a lack of sensory axon bifurcation, thereby mimicking observations from global GC-B knock-out mice. In contrast, no alterations in axon bifurcation were found in a glutamate-substituted mouse mutant of GC-B (GC-B-7E) that cannot be inactivated by dephosphorylation.

In conclusion our studies demonstrated that phosphorylation of regulatory serine and threonine residues in the KHD is required for activation of cGMP generation by GC-B and physiological function such as bone growth and axon bifurcation.

**Funding**

*This work was supported by DFG grants SFB 665 and FOR 2060.*

***Competing interest***

*The authors declare no conflict of interest.*

## A59 Functional β_3_-Adrenoceptor redistribution impairs NO/cGMP/PDE2 signaling in heart failure

### Sophie Schobesberger^1^, Peter Wright^2^, Claire Poulet^2^, Catherine Mansfield^2^, Andreas Friebe^3^, Sian E. Harding^2^, Viacheslav O. Nikolaev^1^, Julia Gorelik^2^

#### ^1^Institute of Experimental Cardiovascular Research, University Medical Center Hamburg-Eppendorf, 20246, Hamburg, Germany; ^2^Department of Myocardial Function, Imperial College London, Hammersmith Hospital, W12 0NN, London, UK; ^3^Institute of Physiology,Universität Würzburg, 97070, Würzburg, Germany

##### **Correspondence:** Sophie Schobesberger **(**s.schobesberger@uke.de**)**

**Introduction:**

Cardiomyocyte β_3_-adrenoceptor (β_3_-AR) levels are increased in cardiac diseases where they alleviate pathological hypertrophy and cellular remodelling through 3’,5’-cyclic guanosine monophosphate (cGMP) signalling [1].. However, the subcellular compartmentation of β_3_-AR/cGMP signaling, its regulation by phosphodiesterases (PDEs) and its alterations in heart failure require further investigation.

**Objective**:

To directly visualize β_3_-AR/cGMP signaling, study receptor localization and β_3_-AR microdomain regulation by PDEs as well as receptor interaction partners in healthy and failing cardiomyocytes.

**Methods and Results:**

Adult rat ventricular cardiomyocytes expressing the cGMP-specific Förster Resonance Energy Transfer (FRET) biosensor red cGES-DE5 were stimulated with the β-AR agonist isoprenaline. This led to the generation of cGMP in a β_3_-AR/NO/soluble guanylyl cyclase (sGC)-dependent manner. The PDEs 2 and 5 proved to be the main degraders of β_3_-AR/cGMP as determined by selective, pharmacological inhibition of cGMP PDEs. FRET in combination with scanning ion conductance microscopy revealed a shift of functional β_3_-ARs from the T-tubules of healthy cardiomyocytes to unstructured membrane areas of cardiomyocytes in heart failure. This shift was also inducible in healthy cells by disrupting the scaffolding domain of Caveolin 3 (C3SD) with the TAT-C3SD peptide. In heart failure, significantly lower β_3_-AR-stimulated cGMP levels and increased PDE2 activity were detected. Immunocytochemical staining also revealed reduced sGC association with caveolin-rich membrane fractions in failing cells.

**Conclusions:**

In healthy cardiomyocytes functional β_3_-ARs are localized in T-tubules and β_3_-AR/cGMP levels are regulated by PDE2 and PDE5. Heart failure leads to redistribution of functional β_3_-ARs and sGC from T-tubules to detubulated membrane areas. Together with increased PDE2 activity, this can disrupt receptor-associated cGMP microdomains and impair potentially protective β_3_-AR/cGMP signaling.

***Competing interest***

*There is no conflict of interest for the presented work.*

**Funding**

*The work is funded by the Deutsche Forschungsgemeinschaft.*

**References:**

[1] Cannavo A1, Koch WJ. **Targeting β3-Adrenergic Receptors in the Heart: Selective Agonism and β-Blockade.** *J Cardiovasc Pharmacol*. 2017 69(2):71-78.

## A60 Irreversible activation of soluble guanylate cyclase by cinaciguat

### Alexander Kollau^1^, Marissa Opelt^1^,Gerald Wölkart^1^, Antonius C.F. Gorren^1^, Michael Russwurm^2^, Doris Koesling^2^, Astrid Schrammel^1,^ Bernd Mayer^1^

#### ^1^ Department of Pharmacology and Toxicology, University of Graz, A 8010 Graz, Austria; ^2^ Department of Pharmacology and Toxicology, Ruhr University Bochum, D 44780 Bochum, Germany

##### **Correspondence:** Astrid Schrammel **(**astrid.schrammel-gorren@uni-graz.at**)**

**Clinical background:**

**Fig. 12:**
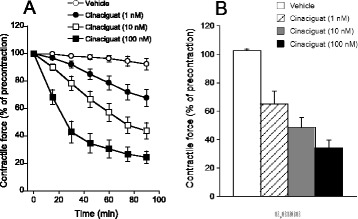
**(Abstract A60).** Effect of cinaciguat on vasorelaxation. Cinaciguat (1-100 nM) induced time-dependent relaxation of K^+^-precontracted porcine coronary arteries **a**. The dilatory effect of cinaciguat was not reversed upon extensive washout of the drug for 1 hour **b.** Data represent mean values±SEM of 7 individual experiments

Activation of soluble guanylyl gyclase (sGC) is regarded as an innovative therapeutic concept for the treatment of various pathologies associated with oxidative stress including pulmonary hypertension and acute heart failure. Belonging to the group of so-called sGC activators, cinaciguat (BAY 58-2667) and BAY 60-2770 are supposed to preferentially stimulate either oxidized (ferric) or heme-depleted (apo) sGC [1]. This concept has been challenged by studies demonstrating complete relaxation of intact uninjured blood vessels exposed to these drugs [2]. To investigate this apparent discrepancy, the effect of cinaciguat on relaxation of micro- and macrovessels was tested and compared to tissue cGMP levels. Moreover, using purified sGC from bovine lung the mechanism of enzyme activation by cinaciguat was studied in greater detail. Organ bath experiments showed that the drug caused time-dependent relaxation of precontracted coronary vessels (See Fig. 12). The dilatory response was not affected by extensive washout over a period of 1 hour indicating virtually irreversible relaxation. Cinaciguat-induced relaxation was associated with a time- and concentration-dependent increase in vascular cGMP measured by radioimmuno assay. Washout of the drug for 1 hour had no effect on vascular cGMP levels. The irreversible type of action was also observed in experiments with cultured porcine aortic endothelial cells.

Activation of purified sGC by cinaciguat resulted in moderate cGMP formation *i.e.* ~10% of maximal activity induced by the NO donor sodium (Z)-1-(N,N-diethylamino)diazen-1-ium-1,2-diolate (DEA/NO). Oxidation of the enzyme with 1H-[1,2,4] oxadiazolo[4,3-a]quinoxalin-1-one (ODQ) to its ferric state did not significantly increase cinaciguat-induced sGC activity. The degree of ferric enzyme activation was neither affected by concentration (1-100 nM of cinaciguat) nor incubation time (10-90 min). By contrast, heme-depleted sGC was activated by cinaciguat in a concentration-dependent manner yielding values comparable or higher than measured in the presence of a maximally active concentration of the NO donor. According to the results obtained with isolated vessels cinaciguat-induced sGC activation was irreversible (at least for 90 min).

**Conclusion:**

Cinaciguat induced irreversible relaxation of intact uninjured coronary arteries and aortas. The irreversible mode of action was also observed in experiments with cultured porcine endothelial cells and purified bovine lung sGC. Assuming that under physiological conditions cells contain a small pool of sGC as heme-free species our data indicate that cinaciguat is capable of continuously shifting the cellular equilibrium between NO-sensitive sGC and heme-free apo sGC towards latter population. Hypotensive side effects of cinaciguat therapy observed in clinical trials [3] may substantiate this hypothesis.

**Conflict of interest**

*None*

**References:**

[1]. Evgenov OV, Pacher P, Schmidt PM, Haskó G, Schmidt HHHW and Stasch JP: **NO-independent stimulators and activators of soluble guanylate cyclase: Discovery and therapeutic potential.** *Nat Rev Drug Discov* 2006, **5**:755-768

[2]. Jabs A, Oelze M, Mikhed Y, Stamm P, Kröller-Schön S, Welschof P, Jansen T, Hausding M, Kopp M, Steven S, Schulz E, Stasch JP, Münzel T and Daiber A: **Effect of soluble guanylyl cyclase activator and stimulator therapy on nitroglycerin-induced nitrate tolerance in rats.** *Vasc Pharmacol* 2015, **71**:181-191

[3]. Gheorghiade M, Greene SJ, Filippatos G, Erdmann E, Ferrari R, Levy PD, Maggioni A, Nowack C and Mebazaa A: **Cinaciguat, a soluble guanylate cyclase activator: Results from the randomized, controlled, phase IIb COMPOSE programme in acute heart failure syndromes**. *Eur J Heart Fail* 2012, **14**:1056-1066

## A61 Role of protein kinase G signaling in aortic wall maintenance and repair

### Gerburg K Schwaerzer^1^, Darren E Casteel^1^, Nancy D Dalton^1^, Yusu Gu^1^, Shunhui Zhuang^1^, Dianna M Milewicz^2^, Kirk L Peterson^1^, Renate Pilz^1^

#### ^1^Department of Medicine, University of California San Diego, La Jolla, 92093 California, USA; ^2^Division of Medical Genetics and Cardiology, Department of Internal Medicine, The University of Texas Health Science Center at Houston, Houston, 77030, Texas, USA

##### **Correspondence:** Gerburg K Schwaerzer **(**gschwaerzer@ucsd.edu**)**

**Background:**

Aortic aneurysms and dissections account for 1-2% of all deaths in the U.S.; aortic media degeneration is a hallmark of the disease, and involves smooth muscle cell (SMC) dysfunction and elastin degradation. A heterozygous gain-of-function mutation (R177Q) in protein kinase G 1 (PKG1) was recently identified as the cause of early-onset, high-penetrance familial thoracic aortic aneurysms and dissections [1]. Among many other physiological processes, PKG1 controls vascular contractility and keeps smooth muscle cells (SMCs) in a differentiated, quiescent, and contractile state [2].

**Results:**

We generated mice carrying a R177Q (RQ) knock-in mutation, which causes constitutive, cGMP-independent enzyme activation. At 8-12 months of age, heterozygous PKG1RQ/+ mice show aortic media degeneration with increased elastin fiber fragmentation and collagen accumulation, decreased SMC count, and aortic dilatation. The aortic pathology in younger PKG1RQ/+ mice is aggravated by hypertensive stress and results in increased mortality due to aortic rupture after transverse aortic constriction surgery. Generation of reactive oxygen species (ROS) is increased in aortic SMCs from PKG1RQ/+ mice, and there is evidence of oxidative damage and altered contractile and ECM gene expression in the aorta of PKG1RQ/+ mice compared to wild type litter mates.

**Conclusion:**

PKG1-R177Q increases ROS generation in aortic SMCs causing oxidative damage in aortic media, altered contractile and ECM gene expression, and impaired aortic wall maintenance and repair.

***Competing interest***

*The authors declare that they do not have a potentially perceived conflict of interest.*

**References:**

[1]. D-C Guo, E Regalado, DE Casteel, RL Santos-Cortez, L Gong et al.: **Recurrent Gain-of Function Mutation in** ***PRKG1*** **causes Thoracic Aortic Aneurysms and Acute Aortic Dissections.** Am J Hum Genet 2013, 93 (2): 398-404

[2]. T Zhang, S Zhuang, DE Casteel, DJ Looney, GR Boss, RB Pilz: **A cysteine-rich LIM-only protein mediates regulation of smooth muscle-specific gene expression by cGMP-dependent protein kinase.** J Biol Chem 2007, 282 (46): 33367-80

## A62 Pericytes isolated from mouse lung express NO-sensitive guanylyl cyclase (NO-GC) and differentiate into myofibroblasts upon stimulation with TGF-ß

### Fabian Schwiering, Annemarie Aue, Dieter Groneberg, Andreas Friebe

#### Physiologisches Institut, Universität Würzburg, Würzburg, Germany

##### **Correspondence:** Fabian Schwiering **(**fabian.schwiering@uni-wuerzburg.de**)**

**Background:**

Pericytes are vascular cells which can be found in the basal membrane (BM) around blood capillaries. They are closely related to vascular smooth muscle cells (VSMC). However, pericytes can be distinguished from VSMC through morphology and specific markers such as platelet-derived growth factor receptor ß (PDGFR-ß) or desmin. Recent data indicate that pericytes could be the main precursor of myofibroblasts in the lung which deposit extracellular matrix in the interstitium during lung fibrosis.

**Methods:**

Pericytes were isolated from mouse lung and seeded on collagen-coated glass until they reached confluency of ~ 90%. For immunohistochemistry (IHC), the cells were fixed, permeabilized with digitonin or Triton-X-100, stained with specific antibodies and viewed under a confocal fluorescence microscope. Only primary pericytes cultures were used. To investigate differentiation into myofibroblasts, lung pericytes were isolated, seeded onto collagen-coated wells and stimulated with 10 ng/ml transforming growth factor ß (TGF-ß) - a well-known fibrosis trigger in the lung. After 72 h incubation, the cells were lysed and the protein content of α smooth muscle actin (αSMA) and NO-GC was determined using Western blot (WB). Histone H3 was used as housekeeping gene.

**Results:**

The isolated cells were confirmed as pericytes based on fact that 90% were immunopositive for PDGFR-ß and 60% were positive for desmin. Moreover, pericytes from WT were positive for NO-GC in IHC and WB; yet, with both methods, NO-GC was shown to be absent in pericytes from global NO-GC knockout mice (GCKO). While αSMA expression was low in untreated pericytes (IHC and WB), TGF-ß strongly increased the expression of αSMA. Interestingly, TGF-ß also led to an increase in the NO-GC level in pericytes.

**Discussion:**

In summary, we established a protocol to isolate NO-GC-expressing pericytes from mouse lung. These cells can differentiate into myofibroblasts upon treatment with the profibrotic TGF-ß. The identification of pericytes as a precursor of myofibroblasts is an important step in the treatment of lung fibrosis. Pericytes could be a target for a therapy of lung fibrosis. NO-GC stimulators are known to have antiproliferative and/or apoptotic effects. Further studies are directed to test the antifibrotic potential of NO-GC stimulation in vitro and in vivo.

***Competing interest***

*The authors declare no conflict of interest.*

## A63 Conformational, dynamical and oxidation state variations monitored through gas HNOX infiltration and heme replacement by agonists

### Aikaterini I. Argyriou^1^, Garyfalia Makrynitsa^1^, Ioannis I. Alexandropoulos^1^, Andriana Stamopoulou^1^, Marina Bantzi^2^, Athanassios Giannis^2^, Stavros Topouzis^1^, Andreas Papapetropoulos^1,3^, Georgios A. Spyroulias^1^

#### ^1^Department of Pharmacy, University of Patras, GR-26504, Patras, Greece; ^2^Institute of Organic Chemistry, Leipzig University, D-04103 Leipzig,Germany; ^3^School of Health Sciences, Faculty of Pharmacy, University of Athens, GR-15 771, Athens, Greece

##### **Correspondence:** Georgios A. Spyroulias (G.A.Spyroulias@upatras.gr)

Some of the diatomic diffusible molecules, like O2, CO and NO are involve in vital biochemical processes and are classified among the indispensable signaling molecules in all kingdoms of life. Heme-nitric oxide/oxygen binding (H-NOX) domain is found either as a stand-alone protein consisting of approximately 200 amino acids in length or fused to other domains within larger proteins, such as in soluble guanylate cyclase (sGC) and is conserved across eukaryotes and bacteria. sGC-related proteins consist if a heme-binding N-terminal domain (H-NOX) that regulates the catalytic site located in the C-terminal end of the protein. sGC is an heterodimer, comprising by an α1 or α2 subunit combined with β1 subunit catalyzing the conversion of GTP to cGMP. Within sGC, the H-NOX domain in the β1 subunit functions as a sensor for the diffusible signaling agent nitric oxide (NO). In the vasculature, NO increases the activity of sGC by several hundred fold, and results in vasodilation and inhibition of platelet aggregation. Oxidative stress is the key-factor for heme loss of sGC and consequently, the inability of NO activation. This condition leads the enzyme to degradation and as a result the NO signaling pathway is disrupted. Several chemical compounds like cinaciguat (BAY 58-2667), are heme-independent activators which protects sGC from proteasomal degradation.

However, the critical determinants of the gas filtering and the coordination to the iron(II) heme are still not completely elucidated. In the present study we investigate the conformational and the electronic properties of the HNOX domain from Nostoc sp. which exhibits 35% sequence homology with the corresponding one from human sGC. For the structural and dynamic characterization of the protein during the Fe(II) oxidation state, we performed 2D and 3D homo- and heteronuclear NMR experiments in solution as well as UV-visible spectroscopy. Furthermore, we conducted experiments in order to examine the changes upon the heme molecule after the interaction with chemical compounds and oxidizing agents (NO, BAY 58-2667, ODQ). The manner by which the oxidation status of Fe(II)-Protoporphyrin IX prosthetic group affect the structure and the dynamical behavior of HNOX domain will allow us to gain considerable information regarding the regulation of sGC activation/stimulation and will ultimately allow the rational design of ligands interacting in a largely predictable way with sGC.

**Acknowledgments:**

*We acknowledge partial support from EU FP7-REGPOT-2011 “SEE-DRUG” (nr. 285950 to A.P., S.T.& G.S.).*

***Competing interest*** *:*

*The authors have no conflict of interest to declare.*

**Reference:**

[1]. II Alexandropoulos, AI Argyriou, KD Marousis, S Topouzis, A Papapetropoulos, GA Spyroulias: ^**1**^ **H,** ^**13**^ **C,** ^**15**^ **N backbone and side-chain resonance assignment of Nostoc sp. C139A variant of the heme-nitric oxide/oxygen binding (H-NOX) domain.** *Biomol NMR Assign.* 2016, 10: 395-400. 1998, 80: 65-69

## A64 sGC maturation in cells: mechanisms and regulation

### Dennis J. Stuehr^1^, Arnab Ghosh^1^, Yue Dai^1^, and Saurav Misra^2^

#### ^1^Department of Pathobiology, Lerner Research Institute, Cleveland Clinic, Cleveland, Ohio 44195 USA; ^2^Kansas State University, Manhattan, Kansas 66506 USA

##### **Correspondence:** Dennis J. Stuehr **(**stuehrd@ccf.org**)**

After translation, the sGC-β subunit must undergo heme insertion and then associate with a partner sGC-α subunit in order to create a functional heterodimer. We have uncovered important roles for NO and hsp90 in directing these processes, and are investigating the molecular and cellular basis for their effects. In cells, our work suggests only the immature and heme-free form of sGC-β is associated with hsp90, and it does not interact with sGC-α until after the hsp90 helps to drive sGC-β heme insertion in an ATP-dependent process. In several types of resting cells, and in tissues *ex vivo*, we find that a mixture of immature and mature sGC-β exists, and that NO exposure can quickly cause the cells to up-regulate heme insertion into the apo-sGC-β and so drive maturation to the sGC heterodimer. This effect is seen either with added NO donor or in transwell systems that contain physically-separated NOS- and sGC-expressing cells. The NO-driven sGC-β maturation depends on hsp90, because it is blocked by hsp90 inhibitors or antagonized by an ATP-ase-deficient hsp90 variant. If the NO exposure (flux) exceeds a certain threshold, the cell’s sGC-β heme insertion becomes inhibited, coincident with a build up of SNO on the sGC-β subunit, and its return to association with hsp90. We are currently determining if and how the SNO events and heme status of sGC-β are involved in directing its changes in protein interactions in the cells. In molecular studies with purified sGC-β and hsp90 proteins, we found their interaction involved the hsp90 M domain and the PAS domain of sGC-β. HxD and hydroxyl-radical footprinting studies have identified specific regions within these domains as likely interacting sites. Molecular modelling suggests that hsp90 M domain interacts with regions of the sGC-β PAS that are also likely involved in sGC heterodimer formation. Point mutation or deletion within these regions of PAS blocked its interaction with purified hsp90, and we are now testing the importance of these interactions for enabling sGC hsp90 interaction, heme insertion, and heterodimer formation within cells.

## A65 Inhibition of kainate receptor by extracellular cGMP

### Boris Tchernychev^1^, Joon Jung^1^, Guang Liu^1^, Inmaculada Silos-Santiago^2^, Gerhard Hannig^1^

#### ^1^Ironwood Pharmaceuticals, Cambridge, MA 02142, United States; ^2^Decibel Therapeutics, Cambridge, MA 02142, United States

##### **Correspondence:** Boris Tchernychev **(**btchernychev@ironwoodpharma.com**)**

**Background:**

ATP-binding cassette transporters mediate active efflux of intracellular cGMP into interstitial space. This extracellular cGMP was shown to modulate several important physiological processes such as neuronal survival [1] and visceral hypersensitivity [2]. The identification of the still elusive molecular target(s) of extracellular cGMP represents an important step to further enhance our understanding of its role in these processes. Here, we have employed a computational approach to identify the kainate receptor as a potential target for extracellular cGMP.

**Materials and Methods:**

Ligand similarity and docking analyses were used for target identification, and functional assays in primary neurons and transfected cells to evaluate cGMP as a modulator of kainate receptor function. The effect of guanine nucleotides on the kainate receptor-mediated ion flux was studied using patch clamp technique. Cellular permeability of extracellular cGMP was evaluated using a cGMP-Glo sensor stably expressed in HEK 293 cells.

**Results:**

Target identification approach using the structure of cGMP and its 3-dimensional similarity to ligands with known target was utilized. Ligand-gated ion channels were identified as primary hits of ligand similarity to cGMP, with the class of kainate receptors revealing the highest similarity scores. Next, we used CHO cells transfected with GluK2/5 kainate receptor to evaluate the effect of extracellular cGMP on receptor conductivity and found GluK2/5-mediated ion current inhibition by cGMP in a concentration-dependent manner. No effect on GluK2/5-mediated ion flux was observed when cGMP was tested in agonist or desensitization mode, with similar results found for GMP and guanosine. Moreover, when the effect of extracellular cGMP on kainate-receptor activity was studied in cultured primary human DRG neurons, two types of the kainate-induced currents were observed: desensitizing and non-desensitizing currents. Treatment of these neurons with cGMP had no effect on desensitizing ion current evoked by kainate application. In contrast, extracellular cGMP completely inhibited non-desensitizing ion flux, indicating that human DRG neurons express different sub-sets of heteromeric kainate receptors which can differ in their response to kainate and in their sensitivity to extracellular cGMP.

**Conclusions:**

In summary, our computational *in silico* ligand similarity scoring model has identified the GluK2/5 kainate receptor as a potential molecular target for extracellular cGMP, a finding subsequently supported in *in vitro* biochemical and functional assays. However, *in vivo* studies are needed to further confirm the identity of the GluK2/5 kainate receptor as a target of extracellular cGMP in relevant animal models.

**Acknowledgments:**

Supported by Ironwood Pharmaceuticals, Inc., and Allergan.

**Conflict of Interest:**

Boris Tchernychev, Joon Jung, Guang Liu and Gerhard Hannig are employees of Ironwood Pharmaceuticals with stock ownership. Inmaculada Silos-Santiago was an employee of Ironwood Pharmaceuticals at the time of this study.

**References:**

[1] Albrecht P, Henke N, Tien ML, Issberner A, Bouchachia I, Maher P, Lewerenz J, Methner A: **Extracellular cyclic GMP and its derivatives GMP and guanosine protect from oxidative glutamate toxicity.** Neurochem. Int. 2013, 62(5):610-9

[2] Castro J, Harrington AM, Hughes PA, Martin CM, Ge P, Shea CM, Jin H, Jacobson S, Hannig G, Mann E, Cohen MB, MacDougall JE, Lavins BJ, Kurtz CB, Silos-Santiago I, Johnston JM, Currie MG, Blackshaw LA, Brierley SM.: **Linaclotide inhibits colonic nociceptors and relieves abdominal pain via guanylate cyclase-C and extracellular cyclic guanosine 3',5'-monophosphate.** Gastroenterology 2013, 145(6):1334-46

## A66 A non-canonical chemical feedback self-limits nitric oxide-cyclic GMP signaling in health and disease

### Vu Thao-Vi Dao^1,2#^, Martin Deile^3#^, Pavel I. Nedvetsky^4^, Andreas Güldner^5^, César Ibarra-Alvarado^6^, Axel Gödecke^7^ and Harald H.H.W. Schmidt^1^

#### ^1^Department for Pharmacology and Personalized Medicine, CARIM, FHML, Maastricht University, Maastricht, The Netherlands; ^2^Department of Pharmacology, Johannes Gutenberg University, Mainz, Germany; ^3^Asklepios-Klinik Radeberg, Dresden, Germany; ^4^Vesalius Research Center, Katholieke Universiteit Leuven, Leuven, Belgium; ^5^Residency Anaesthesiology, Department of Anaesthesiology und Critical Care Medicine, Technische Universität, Dresden, Germany; ^6^Facultad de Química, Universidad Autónoma de Querétaro, Santiago de Querétaro, Mexico; ^7^Institut für Herz- und Kreislaufphysiologie Heinrich-Heine-Universität, Düsseldorf, Germany

##### **Correspondence:** Harald H.H.W. Schmidt **(**h.schmidt@maastrichtuniversity.nl**)**; Vu Thao-Vi Dao

#Both authors contributed equally

**Fig. 13:**
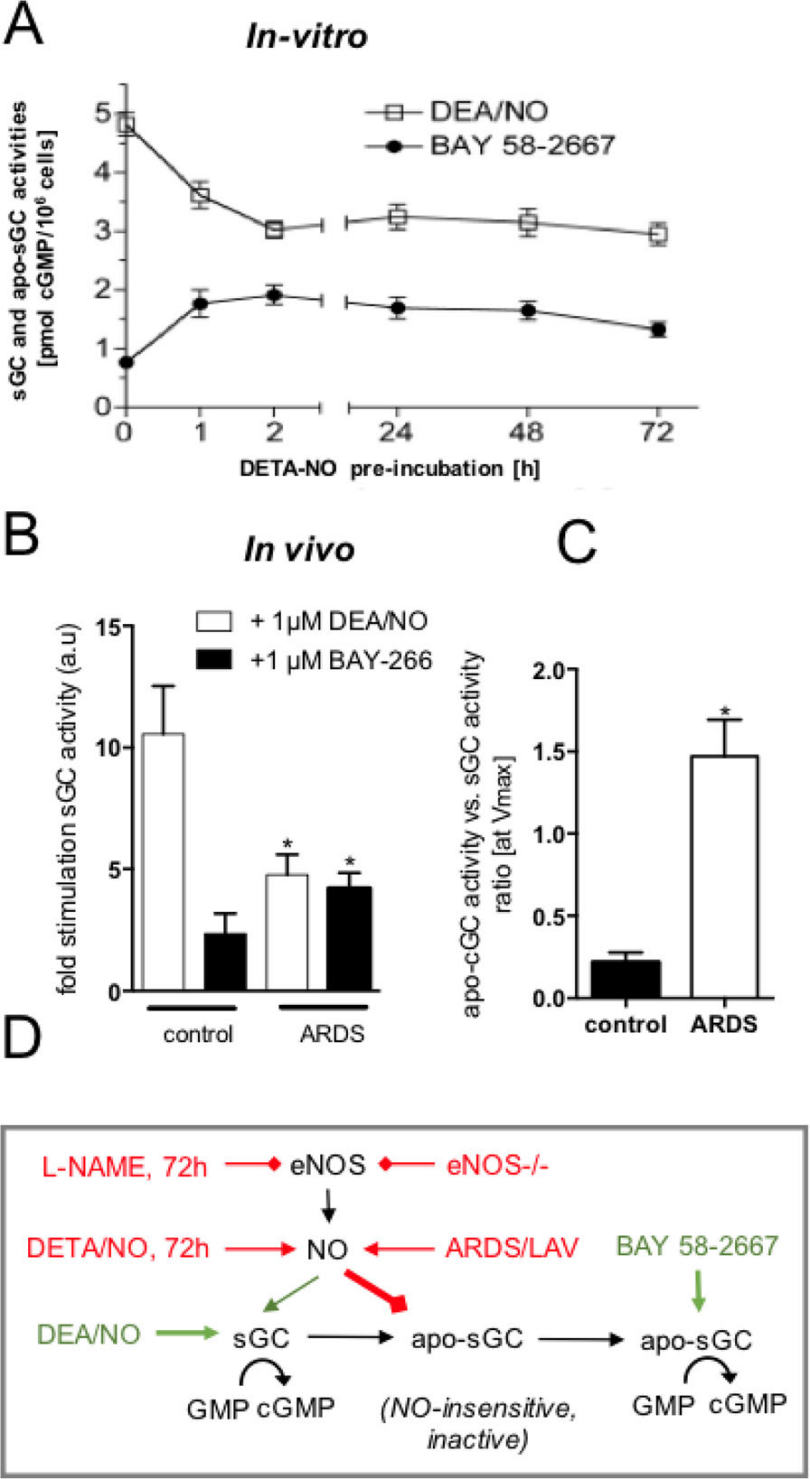
**(Abstract A66]** See text description.)

Nitric oxide signals through soluble guanylate cyclase (sGC) generating cyclic guanosine monophosphate (cGMP) to exert essential protective functions in the cardiovascular system. The possible feedback regulation of this important pathway in health and disease is incompletely understood. Here we show in pulmonary artery endothelial cells that chronic exposure to exogenous NO donor drugs or, importantly, also to endogenous NO down-regulates sGC protein and cGMP formation up to three-fold. Mechanistically, this effect is surprisingly cGMP-independent and involves oxidative loss of the sGC’s NO-binding heme and formation of apo-sGC, insensitive to NO. Alternative mechanisms, such as thiol-dependent modulation appeared not to be involved. In an in vivo disease model with high levels of NO, acute respiratory distress syndrome, a similar self-limiting redox shift from NO-sensitive to NO-insensitive apo-sGC was observed (see Fig. 13). These data suggest a bimodal, non-equilibrium role for NO signaling by acutely stimulating cGMP formation and chronically redox inactivating the enzyme towards its apo-form defining a self-limiting chemical feedback. Our findings add to the limitations of still widely used NO donor drugs. The NO-induced apo-state of sGC can however be functionally recovered by heme mimetic apo-sGC activator drugs.

***Competing interest***

*The authors declare no conflict of interest.*

## A67 Conditional genetic ablation of cGMP-dependent protein kinase I impairs stress responses in mice

### Angelos Vachaviolos^1,#^, Andrea Gerling^1,#^, Martin Thunemann^1^, Stefan Z. Lutz^2^, Hans-Ulrich Häring^2^, Marcel A. Krüger ^3^, Bernd J. Pichler^3^, Michael J. Shipston^4^, Susanne Feil^1^, Robert Feil^1^

#### ^1^Interfakultäres Institut für Biochemie, University of Tübingen, Tübingen, Germany; ^2^Department of Internal Medicine, Division of Endocrinology, Diabetology, Vascular Disease, Nephrology and Clinical Chemistry, University of Tübingen, Tübingen, Germany; ^3^Werner Siemens Imaging Center, Department of Preclinical Imaging and Radiopharmacy, University of Tübingen, Tübingen, Germany; ^4^Centre for Integrative Physiology, School of Biomedical Science, Hugh Robson Building, University of Edinburgh, EH8 9XD Edinburgh, Scotland, United Kingdom

##### **Correspondence:** Angelos Vachaviolos **(**angelos.vachaviolos@uni-tuebingen.de**)**

# equal contribution

The important role of the cGMP-signalling pathway and its major effector protein, cGMP-dependent protein kinase I (cGKI), in the cardiovascular system is well established. In addition, cGMP-signalling has long been proposed to play a role in the regulation of stress responses via the modulation of the hypothalamic-pituitary-adrenal (HPA) axis. However, the underlying mechanisms including the cell types and cGMP effectors involved are largely unknown.

To shed light on the role of cGKI in stress responses, we studied two conditional cGKI mouse models, (1) mice lacking cGKI in the nervous system, which were generated using a Nestin-Cre line (cGKI-brain-KO mice), and (2) mice, in which cGKI expression was abolished from all cells except smooth muscle cells (cGKI-SM-rescue mice). We exposed cGKI mutant and control mice to different stress types and monitored stress hormone levels by immunoassays, core body temperature by telemetry and brown adipose tissue (BAT) activity, indirectly, by [^18^F]FDG PET imaging.

We detected cGKI expression in distinct regions of the hypothalamus, pituitary and adrenal gland, as well as in vessels and nerve bundles of BAT. Interestingly, we found abundant expression of cGKI in fibroblast-like cells evenly scattered between the steroidogenic cells of the adrenal cortex and between brown adipocytes in BAT. Injection of the “immunological stressor” interleukin-1β elicited increased plasma concentrations of adrenocorticotrophic hormone (ACTH) and corticosterone (CORT). While cGKI-brain-KO mice had similar levels of ACTH and CORT as controls, cGKI-SM-rescue mice showed significantly higher ACTH and, surprisingly, lower CORT levels than control mice. Compared to controls, CORT release after direct injection of ACTH was attenuated in cGKI-SM-rescue mice, suggesting reduced ACTH sensitivity and/or steroidogenic capacity of their adrenal glands. Cold-stress-induced thermoregulation was also defective in cGKI mutant mice. cGKI-brain-KO mice were unable to maintain their core body temperature when subjected to cold-stress at 4 °C. PET imaging of cold-stressed cGKI-brain-KO mice revealed reduced [^18^F]FDG uptake in BAT compared to controls, indicating a dysfunction of their BAT.

In sum, this study demonstrates the important roles of cGKI in stress responses in mice.

Our data suggest that cGKI is mandatory in non-neuronal/Nestin-negative and neuronal/Nestin-positive cells for a proper stress response in the HPA axis and BAT, respectively.

***Competing interest***

*The authors declare no conflict of interests.*

## A68 Combined treatment with miR-425 and miR-155 enhances the reduction of atrial natriuretic peptide expression and downstream cGMP levels

### Sara Vandenwijngaert^1^, Clara D. Ledsky^1^, Obiajulu Agha^1^, Dongjian Hu^2^, Ibrahim J. Domian^2,3^, Emmanuel S. Buys^1*#*^, Christopher Newton-Cheh^2,4,5*#*^, Donald B. Bloch^1,6*#*^

#### ^1^Department of Anesthesia, Critical Care, and Pain Medicine, Massachusetts General Hospital and Harvard Medical School, Boston, Massachusetts, USA; ^2^Cardiovascular Research Center, Department of Medicine, Massachusetts General Hospital and Harvard Medical School, Boston, Massachusetts, USA; ^3^Harvard Stem Cell Institute, Cambridge, Massachusetts, USA; ^4^Program in Medical and Population Genetics, Broad Institute, Cambridge, Massachusetts, USA; ^5^Center for Genomic Medicine, Massachusetts General Hospital and Harvard Medical School, Boston, Massachusetts, USA; ^5^Division of Rheumatology, Allergy and Immunology, Department of Medicine, Massachusetts General Hospital and Harvard Medical School, Boston, Massachusetts, USA

##### **Correspondence:** Sara Vandenwijngaert **(**svandenwijngaert@mgh.harvard.edu**)**

^#^ Contributed equally

**Background:**

MicroRNA-425 and miR-155 repress expression of *NPPA*, the gene encoding atrial natriuretic peptide (ANP). We investigated whether treatment with a combination of miR-425 and miR-155 resulted in an additive effect on *NPPA* repression in human cardiomyocytes.

**Materials and Methods:**

Human embryonic stem cell derived cardiomyocytes (hESC-CMs) were transfected with negative control miRNA, miR-425, miR-155, or a combination. The total amount of transfected miRNA was held constant under all conditions by adding negative control miRNA. Two days after transfection, *NPPA* expression was measured via RT-qPCR. N-terminal (Nt) proANP protein levels in cardiomyocyte media were assessed using an enzyme-linked immunosorbent assay (ALPCO). A novel assay was developed to measure the effects of cardiomyocyte miRNA transfection on downstream cGMP levels. Briefly, COS7 cells expressing the ANP receptor natriuretic peptide receptor 1 (NPR1) fused with turboGFP were exposed for two hours to media collected from hESC-CMs transfected with miRNAs. Next, cGMP levels in these COS7 cells were measured using an enzyme immunoassay (Cayman Chemical).

**Results:**

MicroRNA-425 and miR-155 each decreased *NPPA* expression in hESC-CMs over a wide range of concentrations, with a significant reduction at concentrations as low as 1 nM. The combination of 0.5 nM miR-425 and 0.5 nM miR-155 decreased cardiomyocyte *NPPA* expression to a greater extent than either 0.5 nM miR-425 or 0.5 nM miR-155 alone (see Table). In addition, the combination of miR-425 and miR-155 was more effective at decreasing secreted Nt proANP protein levels than either miRNA alone (see Table). Even in comparison with *NPPA* repression elicited by 1 nM miR-425 or 1 nM miR-155, combining 0.5 nM miR-425 and 0.5 nM miR-155 reduced *NPPA* expression to a greater extent (see Table). The additive effect of miR-425 and miR-155 on cardiomyocyte ANP expression and secretion was associated with a greater decrease in cyclic 3’,5’-guanosine monophosphate (cGMP) levels in NPR1-expressing cells. Combining 0.5 nM miR-425 and 0.5 nM miR-155 resulted in a greater reduction in downstream cGMP levels compared to 1 nM of miR-425 or 1 nM miR-155 (see Table 4).

**Table 4:** **(Abstract A68).** Additive effect of combining miR-425 and miR-155 on *NPPA* expression and downstream cGMP levels

	1 nM NC miR	0.5 nM miR-425 + 0.5 nM NC miR	0.5 nM miR-155 + 0.5 nM NC miR	0.5 nM miR-425 + 0.5 nM miR-155
*NPPA* expression	1.00±0.02	0.88±0.04	0.87±0.03	0.64±0.04
Nt proANP levels	1.00±0.20	0.75±0.06	0.89±0.25	0.52±0.08
	1 nM NC miR	1 nM miR-425	1 nM miR-155	0.5 nM miR-425 + 0.5 nM miR-155
*NPPA* expression	1.00±0.03	0.84±0.04	0.83±0.03	0.66±0.02
cGMP levels	1.00±0.08	0.86±0.05	0.90±0.07	0.76±0.05

Values (average±SEM) are shown relative to the negative control (NC) miRNA values

**Conclusions:**

These data suggest that greater target repression is obtained by administering lower doses of two cooperative miRNAs, which might also produce fewer off-target effects and thus more specific target regulation.

***Competing interest***

*The authors have no conflict of interest to report.*

## A69 NO/cGMP signaling as important modulator of murine small intestinal motility

### Barbara Voussen, Katharina Beck, Nadine Mauro, Jonas Keppler, Andreas Friebe

#### Physiologisches Institut, Universität Würzburg, Würzburg, Germany

##### **Correspondence:** Barbara Voussen **(**barbara.voussen@uni-wuerzburg.de**)**

**Background:**

Gastrointestinal (GI) motility and peristalsis originate from coordinated movements of circular and longitudinal smooth muscle layers. In the enteric nervous systems, NO is released from nitrergic neurons as a major inhibitory neurotransmitter. GI diseases affecting motility are often associated with impaired nitrergic signaling. The specific role of nitrergic inhibitory signaling on the circular and longitudinal muscle layers (CM and LM, respectively) in the small intestine has not been clearly determined yet. Therefore, in the present study, we investigated the NO-mediated influence on these two muscle layers in murine ileum.

**Methods:**

As NO-sensitive guanylyl cyclase (NO-GC) is the main receptor for NO in the GI tract, we first looked for NO-GC expression in murine ileum via immunohistochemistry. For functional analyses, we measured smooth muscle tone in ileal CM and spontaneous contractions in both ileal muscle layers from mice lacking NO-GC globally (GCKO) and specifically in smooth muscle cells (SMC-GCKO).

**Results:**

In contrast to findings from other parts of the GI tract, the immunohistochemical stainings showed NO-GC expression in platelet-derived growth factor receptor α (PDGFRα)-positive cells but not in interstitial cells of Cajal (ICC). Organ bath experiments revealed NO-GC in SMC to be involved in the maintenance of tone of circular smooth muscle: Addition of an NO-GC inhibitor led to an increase and addition of an NO donor to a decrease in tissue tone. The amplitude of spontaneous contractions in CM was increased in the absence of NO-GC. In contrast, contractile activity in LM was not different between WT and knockout strains. When activated by NO, NO-GC led to suppression of spontaneous contractions in WT longitudinal smooth muscle whereas GCKO tissue was unaffected. To our surprise, NO suppressed spontaneous contractions in longitudinal strips from SMC-GCKO ileum indicating participation of other cell type(s).

**Conclusion:**

NO-GC in SMC is involved in the regulation of tone and amplitude of spontaneous contractions in ileal CM. In LM, NO induces suppression of spontaneous contractions via NO-GC in a non-SMC type.

***Competing interest***

*The authors declare no conflict of interest.*

## A70 The soluble guanylyl cyclase activator, BAY60-2770, abrogates leukocyte adhesion and recruitment in sickle cell disease: *In vitro* and *in vivo* studies

### Wilson A. Ferreira Jr^1^, Hanan Chweih^1^, Pamela L Brito^1^, Camila B Almeida^1^, Carla FF Penteado^1^, Sara SO Saad^1^, Fernando F Costa^1^, Paul S Frenette^3^, Damian Brockschnieder^2^, Johannes-Peter Stasch^2^, Peter Sandner^2^, Nicola Conran^1^

#### ^**1**^Hematology Centre, School of Medical Sciences, University of Campinas (UNICAMP), Sao Paolo, Brazil; ^2^ Bayer AG, Pharmaceuticals - Drug Discovery, Wuppertal, Germany; ^3^ Ruth L. and David S^.^ Gottesman Institute for Stem Cell and Regenerative Medicine Research, Albert Einstein College of Medicine, Bronx, NY, USA

##### **Correspondence:** Wilson A. Ferreira Jr **(**ferreirajr@usp.br**)**

**Fig. 14:**
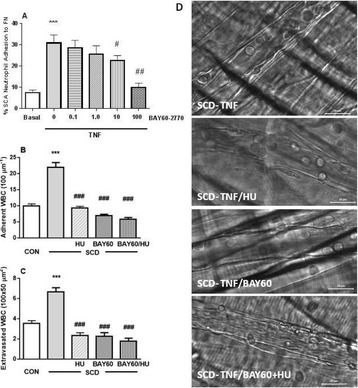
**(Abstract A70).** Effects of BAY60-2770 on TNF-induced leukocyte adhesion and recruitment in SCD mice. Effects of BAY60-2770 on TNF-induced leukocyte adhesion and recruitment in SCD. **a** Neutrophils from steady-state SCA patients (N=7) were incubated, or not, with BAY 60-2770 (0.1 – 100 μM; 120 min) before stimulating with TNF (200 ng/ml) and allowed to adhere to fibronectin-coated plates (30 min, 37^o^C, 5% CO_2_). ***, P < 0.001 compared to basal; #. P<0.05, ##P<0.01, compared to TNF. **b**-**d** Intravital microscopy of the cremaster microcirculation of chimeric SCD mice: (**b**) Leukocyte adhesion and (**c**) leukocyte extravasation at 180 min after TNF-α administration (TNF, 0.5 μg i.p.) and with co-administration of hydroxyurea (HU, 100 μg/kg) and/or BAY60-2770 (BAY 60, 10 μg/mouse). N = 32-39 venules of 3-4 mice per group; ***, P<0.05, compared to CON mice that also received TNF; ###, P<0.05, compared to TNF. Bar= 20μm. Mann-Whitney or AVOVA/Sidak’s test, as appropriate

Many of the complications of the hereditary hemoglobinopathy, sickle cell disease (SCD), result from recurrent vaso-occlusive processes. SCD vaso-occlusion is triggered by the adhesion of leukocytes (particularly neutrophils) and other blood cells to activated endothelium, in response to chronic inflammatory and hypoxic stimuli. The only drug currently approved for SCD therapy is hydroxyurea (HU), a drug that may mediate some of its beneficial effects by releasing nitric oxide (NO). We investigated the effects of the soluble guanylate cyclase (sGC) activator, BAY 60-2770, on SCD leukocyte adhesion/recruitment, using *in vitro* static adhesion and intravital techniques. Human neutrophils (2x10^6^cells/ml) were isolated from healthy controls (CON, N=9) and HbSS sickle cell anemia individuals in steady state (SCA, N=7). SCA neutrophils demonstrate significantly increased adhesion to 20 μg/ml fibronectin ligand (30 min), compared to CON (P<0.05, data not shown), which is further increased by stimulating cells with 200ng/mL TNF-α (TNF; P<0.001, Fig. 14a), a potent cytokine found augmented in SCA. Notably, TNF-stimulated adhesion of SCA neutrophils was significantly diminished by the pre-incubation of cells with 10-100 μM BAY 60-2770 (120 min; Fig. 14a). Intravital microscopy was used to look at the effects of BAY 60-2770 on leukocyte recruitment in the cremaster circulation of chimeric SCD mice (irradiated C57BL6 mice received transgenic SCD Berkeley mouse marrow). Leukocyte recruitment was induced by the administration of murine TNF-α (TNF; 0.5 μg, *i.p.*); in SCD mice, augmented leukocyte adhesion and extravasation occur, leading to vaso-occlusive-like processes in the microcirculation following TNF administration (180 min), when compared to chimeric control mice receiving TNF (CON; C57BL6/C57BL6 transplantation)(Fig. 14b-d). Accordingly, co-administration of HU (100 μg/kg; i.v.) and/or BAY60-2770 (10 μg/mouse; i.v.), at the time of TNF administration, significantly inhibited the adhesion and extravasation of leukocytes in the microcirculation (P<0.001, comp. to vehicle; N=3-4 for each group, 30-36 venules analyzed per group), where the BAY60-2770 compound abolished leukocyte recruitment in a similar manner to that of HU. This study demonstrates the importance of sGC-cGMP signalling in the leukocyte recruitment that triggers vaso-occlusive events in SCD and provides preliminary data to indicate that sGC activators may provide a potential alternative approach for diminishing leukocyte activation and occlusive mechanisms and, possibly, even the painful vaso-occlusive episodes that characterise SCD.

**Funding**

*This work was supported by a restricted research grant of Bayer AG.*

## A71 Cardiovascular actions and tissue distribution of IW-1973 - a clinical-stage soluble guanylate cyclase stimulator

### Daniel P Zimmer^1^, Jenny Tobin^1^, Courtney Shea^1^, Renee Sarno^1^, Kimberly Long^1^, Sarah Jacobson^1^, Kim Tang^1^, Peter Germano^1^, James Wakefield^1^, Ali Banijamali^1^, G-Yoon Jamie Im^1^, James E Sheppeck II^1^, Albert T Profy^1^, G. Todd Milne^1^, Mark G Currie^1^, and Jaime L Masferrer^1^

#### ^1^Ironwood Pharmaceuticals, Cambridge, MA, 02142, USA

##### **Correspondence:** Daniel P Zimmer **(**dzimmer@ironwoodpharma.com**)**

**Introduction:**

The nitric oxide (NO)-soluble guanylate cyclase (sGC)-cGMP pathway is a fundamental signaling pathway in the cardiovascular system with an established role in controlling vasodilation and local blood flow. Systemic and pulmonary hypertension, diabetes, and heart failure are among the diseases associated with endothelial dysfunction and reduced NO-cGMP signalling that may treatable by modulators of this pathway such as IW-1973, a clinical-stage sGC stimulator.

**Methods:**

W-1973 effects on cGMP production were explored in human recombinant sGC enzyme and HEK-293 cell assays. The effect of IW-1973 on relaxation of human resistance arteries was determined. Hemodynamic effects of IW-1973 were compared in normotensive Wistar rats and spontaneously hypertensive rats (SHR). In a Dahl salt-sensitive (DSS) rat model of hypertension, hemodynamics were monitored over 6 weeks of treatment with IW-1973, and effects on the cardiac stress marker NT-proBNP were measured. The pharmacokinetic (PK) profile and tissue distribution of IW-1973 were determined in rats.

**Results:**

IW-1973 stimulated cGMP production in sGC enzyme and HEK-293 cell-based assays (EC_50_ 267 nM and 197 nM, respectively) performed in the presence of the NO donor DETA-NONOate. IW-1973 demonstrated synergy with NO and required the sGC holoenzyme. IW-1973 relaxed pre-contracted human resistance arteries ex vivo (EC_50_ 34.7 nM). IW-1973 demonstrated dose-dependent mean arterial pressure (MAP) reduction in normotensive rats and SHR. The peak effect on MAP reduction after 3 days of dosing at 10 mg/kg/day was notably greater in SHR (27.4 ± 0.9 mmHg SEM) relative to normotensive rats (11.3 ± 1 mmHg SEM). In DSS rats, IW-1973 demonstrated sustained MAP reduction through 6 weeks, and reduced NT-proBNP by 84% at the 10 mg/kg/day dose. IW-1973 (3 mg/kg/day) at 1 week and 6 weeks reduced MAP to a similar extent as losartan (30 mg/kg/day) at both timepoints. Oral bioavailability of IW-1973 in rats was 80-102%, with a Tmax of 8 h, a half-life of 12-22 h, a large volume of distribution (10-11 L/kg), and hepatic clearance. IW-1973 was extensively distributed to tissues including heart, kidney, liver, and lung.

**Conclusions:**

IW-1973 enhanced NO signalling in vitro and reduced MAP in 2 models of hypertension. Effects of IW-1973 on MAP were sustained over 6 weeks, with no evidence of tachyphylaxis. IW-1973 treatment reduced NT-proBNP, a key biomarker of cardiac stress. "IW-1973 had high oral bioavailability and was extensively distributed to tissues. These results support clinical evaluation of this novel sGC stimulator in cardiovascular diseases, particularly those associated with impaired NO signalling.

***Competing interest***

*Authors are current or former employees and shareholders of Ironwood Pharmaceuticals and are developing sGC stimulators for therapeutic applications.*

